# Rapid Generation of P(V)–F Bonds Through the Use of Sulfone Iminium Fluoride Reagents

**DOI:** 10.1021/acs.orglett.3c00274

**Published:** 2023-03-10

**Authors:** Lucy P. Miller, James A. Vogel, Shiraz Harel, Jenna M. Krussman, Patrick R. Melvin

**Affiliations:** Department of Chemistry, Bryn Mawr College, Bryn Mawr, Pennsylvania 19010, United States

## Abstract

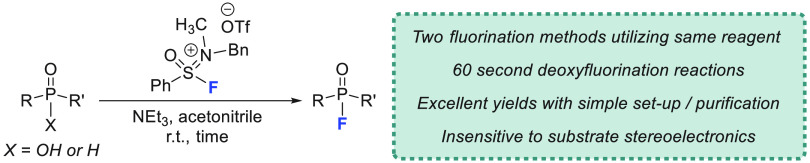

Phosphorus–fluorine bonds have become increasingly relevant in the pharmaceutical industry. To continue their exploration, more efficient synthetic methods are needed. Here, we report the application of sulfone iminium fluoride (SIF) reagents to the synthesis of P(V)–F bonds. The SIF reagents promote the deoxyfluorination of phosphinic acids in just 60 s with excellent yields and scope. The same P(V)–F products can also be synthesized from secondary phosphine oxides using an SIF reagent.

Each year, fluorine continues to gain importance in a variety of areas. Nowhere is this more apparent than in the pharmaceutical industry where the percentage of fluorine-containing compounds has steadily increased since the introduction of fludrocortisone in 1954.^1,2^ This is in large part due to the multitude of beneficial properties that fluorine can instill into target molecules, such as lowering the p*K*_a_ of nearby functional groups, altering conformation, and increasing the metabolic stability of a molecule.^3^ All told, fluorine’s valuable additions have led to its inclusion in 45% of the pharmaceuticals approved by the Food and Drug Administration in 2018 alone.^2^

To maximize the advantages of this powerful element, a variety of fluorine-containing functional groups have been introduced to drug targets. By far, the most common stem from bonds between carbon and fluorine, with aryl and acyl fluorides as well as trifluoromethyl moieties being among the most popular.^2^ However, compounds containing phosphorus–fluorine bonds have also been gaining prominence, particularly those based on P(V). This class of molecule has been used extensively as enzyme inhibitors and mechanistic probes^4^ (Figure 1A), in addition to being organocatalysts in various synthetic techniques.^5^ Furthermore, Sharpless and co-workers have recently utilized P(V)–F compounds to expand on their Nobel Prize winning click chemistry to include phosphorus fluoride exchange (PFEx).^6^

**Figure 1:**
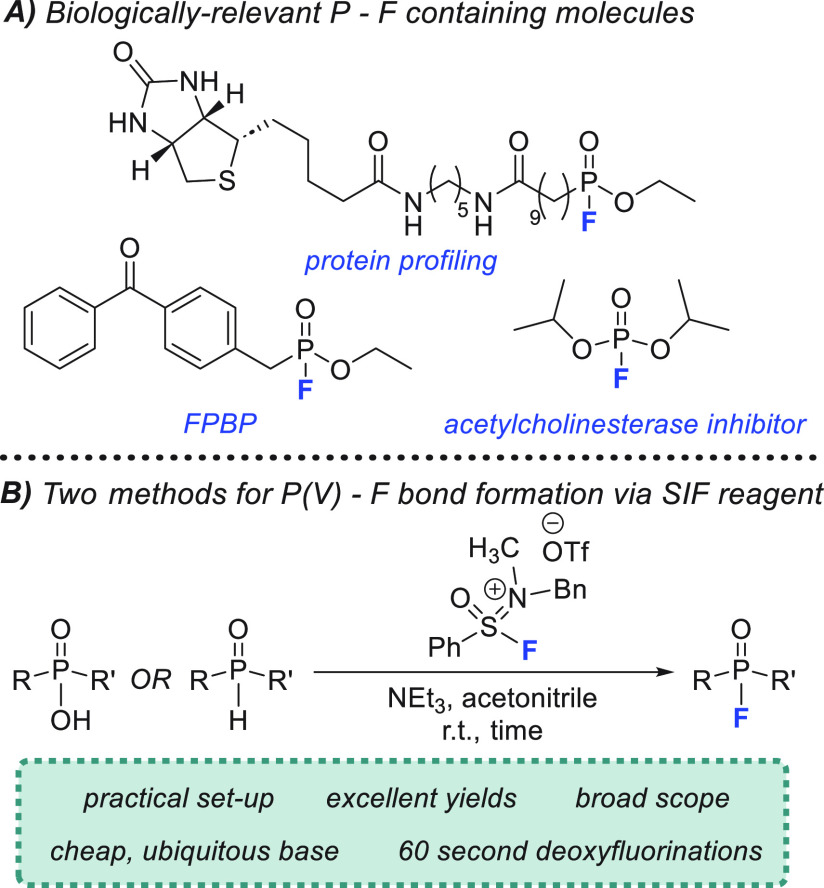
(A) Common organophosphorus compounds containing a P–F bond. (B) Use of SIF reagents for two separate approaches to the synthesis of P(V)–F bonds.

Given their growing importance, protocols are necessary for the formation of key phosphorus–fluorine bonds to facilitate their continued exploration. Among various existing strategies, there are two common pathways which both utilize phosphine oxide substrates: electrophilic fluorination of P(V)–H bonds^7^ and nucleophilic fluorination^8,9^ of P(V)–Cl intermediates. Typically, electrophilic strategies have employed highly reactive compounds such as Selectfluor which can facilitate the conversion of phosphine oxides to the corresponding phosphinic fluorides at room temperature.^7^ The nucleophilic pathway applies an oxidative-fluorination strategy, creating a reactive intermediate that can engage in a substitution reaction with a fluoride source. While various protocols can operate at room temperature, long reaction times and the need for inert atmosphere plague this methodology.^8^ In contrast, only two recent reports have utilized a deoxyfluorination pathway to form phosphinic fluoride products, despite the accessibility of P(V)–OH-containing molecules.^10,11^ This is potentially due to the challenging nature of this transformation, which is demonstrated by the required elevated temperatures (>80 °C), long reaction times (>12 h), and use of inert atmosphere.^10^ Overall, there remains significant room for improvement in the formation of P(V)–F bonds, with a particular need to reduce the time and elevated temperatures required for existing deoxyfluorination strategies.

Recently, our laboratory has developed a new class of S(VI) reagent capable of producing the fastest and most efficient deoxyfluorination reactions of alcohols and carboxylic acids to date.^12^ Sulfone iminium fluorides (SIFs) have demonstrated an incredible propensity toward reactions with hydroxyl functional groups, providing complete conversion to alkyl and acyl fluorides in just 60 s at room temperature. In addition to this impressive reactivity, SIFs are remarkably stable and can be employed under practical conditions without the need for specialized or anhydrous conditions.^12^ However, the SIF reagents had yet to be utilized for more challenging deoxyfluorination substrates, such as with phosphinic acids. To this end, we report the use of an SIF reagent in the synthesis of P(V)–F bonds through two different methodologies, with both strategies characterized by excellent yields and broad substrate scope (Figure 1B). Notably, the deoxyfluorination protocol that has been developed retains the rapid reaction times from our previous study, requiring only 60 s at room temperature to reach completion.

We began our investigation into P(V)–F bond formation using the commercially available diphenylphosphinic acid (**1a**) as the model substrate. First, we tested the previously optimized conditions that had been effective for the deoxyfluorination of alcohols and carboxylic acids (Table 1, entry 1). Gratifyingly, 65% of the fluorinated product was observed by ^19^F and ^31^P NMR spectroscopy after just 60 s at room temperature. Building on this encouraging result, we investigated how both the solvent (entries 1–5) and base (entries 6–9) impacted the conversion to product. With the exception of *N,N*-dimethylformamide and THF (entries 4 and 5), all solvents tested displayed significant quantities of fluorinated product with acetonitrile providing the best conversion when used in conjunction with 1.75 equiv of both SIF and DBU (entry 3: 74%). Continuing with acetonitrile as the solvent, we screened other common nitrogen bases and found that triethylamine (NEt_3_, entry 8: 88%) and proton sponge (entry 9: 85%) provided increased quantities of phosphinic fluoride. Given the drastic difference in cost (NEt_3_: $1.82/mol; DBU: $62.20/mol; proton sponge: $643/mol), we elected to continue with NEt_3_ as the base of choice. With the solvent and base optimized, quantitative conversion to the phosphinic fluoride was observed when the equivalents of both the SIF reagent and NEt_3_ were raised to 2.0 (entry 10: 99%). Finally, we attempted to replicate this reactivity with other S(VI) deoxyfluorination reagents; neither PyFluor nor SulfoxFluor produced any fluorinated product after 24 h when using either our optimized conditions (solvent: acetonitrile; base: NEt_3_) or those previously reported (solvent: toluene; base: DBU).^13^ These results confirm that the increased reactivity of the SIFs over other S(VI) reagents is what enables this transformation to occur, particularly under such mild reaction conditions.

**Table 1:**
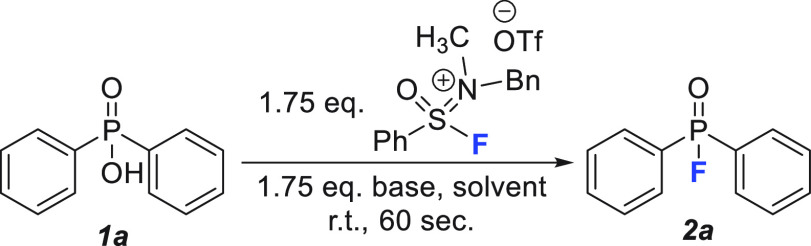
Optimization for the Deoxyfluorination of Diphenylphosphinic Acid Using SIF Reagent

entry	solvent	base	yield^a^
1	DCM	DBU	65
2	Toluene	DBU	57
3	Acetonitrile	DBU	74
4	DMF	DBU	0
5	THF	DBU	6
6	Acetonitrile	Pyridine	63
7	Acetonitrile	2,6-lutidine	36
8	Acetonitrile	NEt_3_	88
9	Acetonitrile	Proton Sponge	85
10^b^	Acetonitrile	NEt_3_	99

a Conditions: diphenylphosphinic acid (0.2 mmol), SIF (0.35 mmol), base (0.35 mmol), solvent (1.0 mL), 22 °C, 60 s. Conversion determined by ^19^F NMR compared to 4-fluoroanisole as internal standard.

b Performed with 2.0 equiv of SIF and NEt_3_.

With an optimized protocol, we next looked to expand this methodology to other symmetric and unsymmetric phosphinic acid substrates (Figure 2). The complete conversion of our model substrate (**2a**) translated to a 96% isolated yield and was amenable to scale-up (2.0 mmol of substrate). Other symmetric phosphinic fluorides with varying substituents (**2b**–**2j**) could be produced in excellent yields with just 60 s of reaction time. Notably, both electron-donating (**2b**, **2c**, **2e**, **2f**) and -withdrawing (**2d**) substituents were well-tolerated, while steric hindrance near the P–OH bond did not impact the high yields of phosphinic fluorides (**2f**, **2g**) produced. A dithienyl substrate was also successful in this methodology, providing the phosphinic fluoride (**2h**) product in 91% conversion. Not limited to just diaryl substrates, dialkyl phosphinic fluorides (**2i**, **2j**) were generated in exceptional yields, in stark contrast to electrophilic fluorination methodologies which struggle to produce phosphinic fluorides with alkyl substituents.^7^

**Figure 2:**
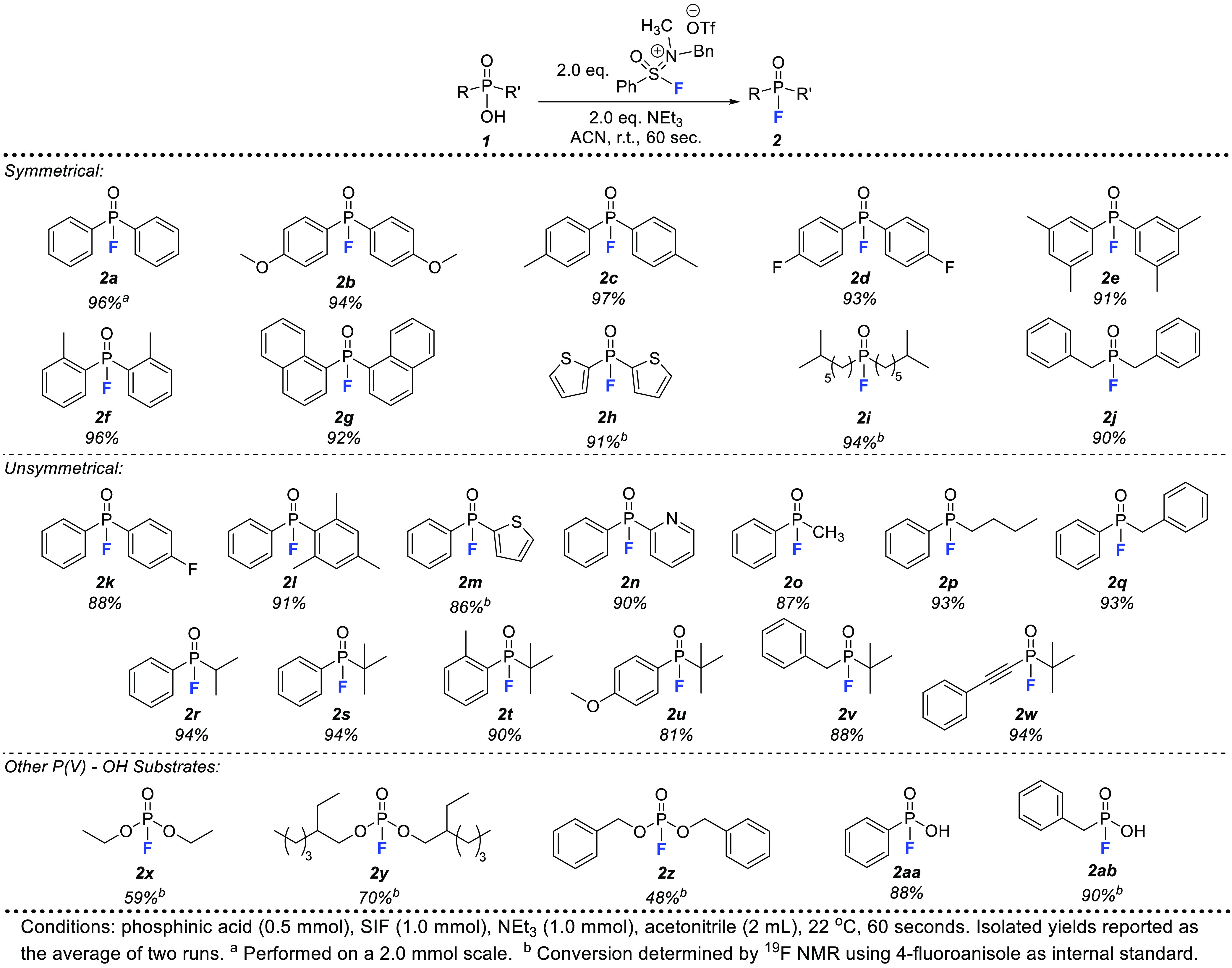
Substrate scope for P(V)–OH deoxyfluorination using SIF reagent.

While previous investigations of phosphinic acid deoxyfluorination have focused mainly on symmetric substrates,^10^ we pursued numerous unsymmetric examples (**2k**–**2w**) to further expand the utility of this SIF-derived method. Again, excellent isolated yields were recorded for all substrates. Notable contributions include several heteroaryl moieties (**2m**, **2n**) as well as a multitude of sterically congested substrates (**2l**, **2r**–**2w**). In particular, *tert*-butyl substituents directly attached to the P(V) could be incorporated with various aryl, alkyl, and alkenyl entities with no decrease in the yields observed. Product **2t** encapsulates this concept best where (*o*-tolyl)(*tert*-butyl) phosphinic fluoride was produced in 90% isolated yield, demonstrating that steric congestion around the P(V)–OH bond has no influence on the success of this methodology.

Finally, we pursued other P(V)–OH substrate classes in phosphates and phosphonic acids. In terms of deoxyfluorination difficulty, the two alkoxy groups of phosphates make for a far more challenging substrate, given the decreased electrophilic nature of the phosphorus. Utilizing three commercially available phosphates, we successfully produced the corresponding fluorophosphate products (**2x**–**2z**) in moderate to good conversions with no changes to the established protocol. Interestingly, while all three aliphatic phosphates shown in Figure 2 yielded significant product, diphenyl phosphate was unsuccessful in this transformation. Next, we pursued the deoxyfluorination of phosphonic acids which contain two P(V)–OH moieties (**2aa**, **2ab**). Initially, it was expected that both hydroxyl groups would be replaced for fluorine; however, phenyl phosphonic acid produces phenyl phosphonofluoridic acid (**2aa**) as the sole product in 88% yield when using the optimized conditions described above. Additional equivalents of SIF and NEt_3_ did not lead to any difluorinated product. This was replicated using benzyl phosphonic acid which also led to a high conversion to benzyl phosphonofluoridic acid (**2ab**) without significant quantities of difluorinated product observed.

During our substrate investigation, phenyl phosphinic acid was screened as a potential valuable entry (Scheme 1). Initially, the desire was for the P–OH moiety to undergo deoxyfluorination while leaving the P–H bond intact. However, the crude mixture of this reaction revealed three fluorinated products in the ^19^F NMR spectrum, with the most prevalent being phenyl phosphinic difluoride (18%, see the Supporting Information). The other two products were monofluorinated species, each coming from a singular substitution of either the hydroxyl group or hydrogen (Scheme 1). Considering SIF reagents were designed primarily with deoxyfluorination in mind, we were compelled to investigate whether the fluorination of the P(V)–H bond in phenyl phosphinic acid could be extended to other substrates, namely secondary phosphine oxides.

**Scheme 1:**
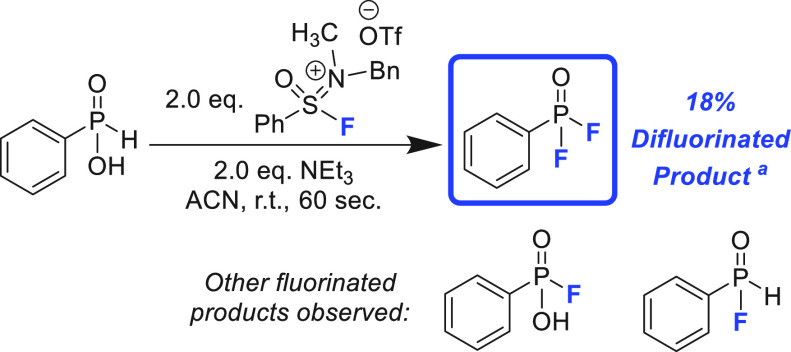
Fluorination of Phenylphosphinic Acid with SIF Reagent Conditions: phenyl phosphinic acid (0.2 mmol), SIF (0.2 mmol), NEt_3_ (0.2 mmol), acetonitrile (1.0 mL) Yield determined by ^19^F NMR with 4-fluoroanisole as internal standard.

Using diphenyl phosphine oxide as a model substrate, we determined that the same optimized conditions for the previous protocol were also ideal for the fluorination of P(V)–H bonds with one notable exception: reaction duration. Unfortunately, the rapid, 60 s reaction times of the deoxyfluorination method did not translate to this protocol; optimal conversion to phosphinic fluoride was achieved after 24 h, albeit still at room temperature. With 2 equiv of SIF and NEt_3_ in acetonitrile for 24 h, diphenyl phosphine oxide could be quantitatively converted to **2a** with an isolated yield of 80% (Figure 3).

**Figure 3:**
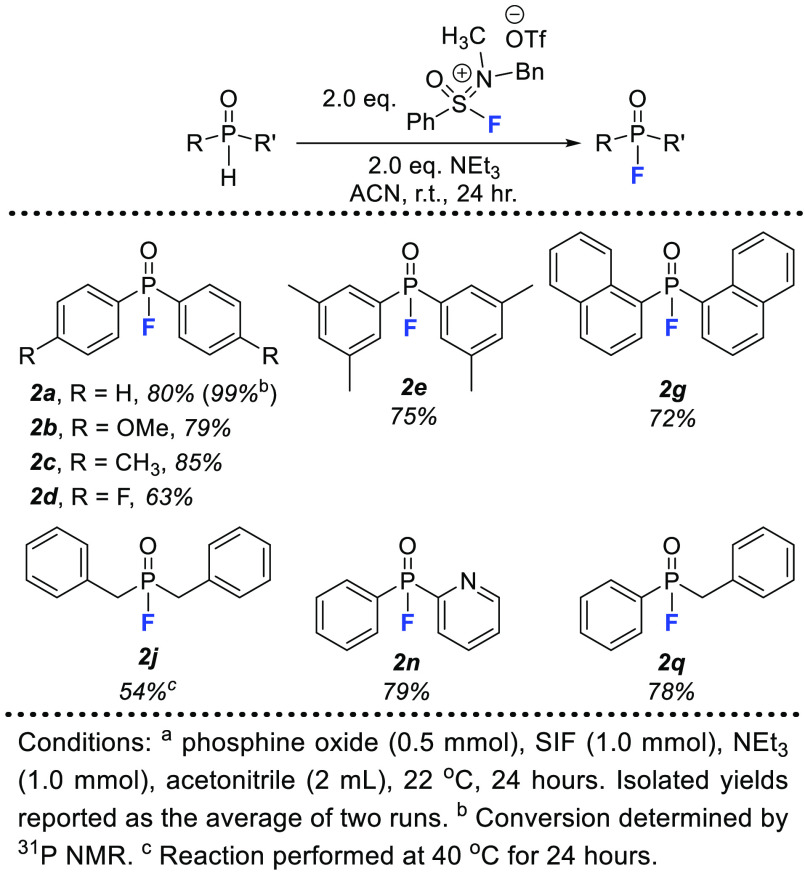
Substrate scope for fluorination of secondary phosphine oxides using SIF reagent.

Using these optimized conditions, we conducted a small substrate scope for the fluorination of secondary phosphine oxides. While the scope is not as extensive as that shown in Figure 2, we successfully transformed both symmetrical and unsymmetrical phosphine oxides into phosphinic fluorides. Once again, both electron-donating (**2b**, **2c**) and -withdrawing (**2d**) substituents were well-tolerated, while substitution at the *ortho* position of the phenyl ring (**2g**) did not diminish the yield of fluorinated product significantly. When a substrate with two alkyl substituents was used (**2j**), the yield was unsatisfactory unless the temperature was raised to 40 °C. Unfortunately, this led to several substrates that were viable for the deoxyfluorination strategy being unsuitable for this pathway. This trend is in agreement with previously established protocols for fluorination of phosphine oxides.^7^

In total, we have established two protocols that create valuable P(V)–F bonds more efficiently. Furthermore, both synthetic strategies are performed by the same sulfone iminium fluoride reagent which enables unprecedented reaction conditions. In particular, deoxyfluorination reactions for over 20 different phosphinic acids proceed with excellent conversions in just 60 s at room temperature, and this protocol was also successfully extended to phosphonic acids and phosphates. To further exemplify the privileged nature of the SIF reagents, other common sulfur(VI)–fluorine reagents failed to engage in these reactions. Secondary phosphine oxides could also be converted to the same phosphinic fluoride products in good to excellent yields, albeit without the rapid reactivity that the SIF reagents demonstrate for the deoxyfluorination pathway. Overall, we have shown that the highly reactive SIFs can provide efficient and practical methods for the installation of critical P(V)–F bonds.

## Data Availability

The data underlying this study are available in the published article and its Supporting Information.

## Supporting Information Available

Experimental procedures, characterization data, and NMR spectra (PDF)
