# Supplementary material for: Rapid Generation of P(V)–F Bonds Through the Use of Sulfone Iminium Fluoride Reagents
Source: Org Lett. 2023 Mar 10;25(11):1834–8. doi: 10.1021/acs.orglett.3c00274 (PMC10043933; doi:10.1021/acs.orglett.3c00274)
Supplement: Supplementary file 1 — ol3c00274_si_001.pdf [file ol3c00274_si_001.pdf]

Supporting Information for:

Rapid Generation of P(V) – F Bonds through the Use of Sulfone Iminium Fluoride Reagents

Lucy P. Miller[ǂ], James A. Vogel[ǂ], Shiraz Harel[ǂ], Jenna Krussman[ǂ], Patrick R. Melvin*[ǂ]

[ǂ] Department of Chemistry, Bryn Mawr College, Bryn Mawr, Pennsylvania 19010

Corresponding Author Email: prmelvin@brynmawr.edu

Table of Contents:

1. General Information – S3
2. Methods and Materials – S3
3. Synthesis of sulfone iminium fluoride reagent – S4
4. Synthesis of phosphine oxide and phosphinic acid substrates – S6
5. Optimization of conditions for the deoxyfluorination of diphenylphosphinic acid – S11
6. Screening of other S(VI) reagents for phosphinic acid deoxyfluorination – S18
7. Deoxyfluorination of phosphinic acids, phosphates and phosphonic acids – S20
8. Reaction of phenylphosphinic acid and SIF reagent – S30
9. Fluorination of secondary phosphine oxides – S31
10. ^{1}H, ^{13}C, ^{19}F and ^{31}P NMR spectra of fluorination products – S34
11. References – S83

1. General Information

NMR spectra were obtained on a Bruker 400 MHz (400.52 MHz for ^{1}H; 376.87 MHz for ^{19}F; 100.71 MHz for ^{13}C). ^{1}H and ^{13}C chemical shifts are reported in parts per million (ppm) relative the residual solvent peak ($CDCl_3$: ^{1}H: δ = 7.26 ppm, ^{13}C: δ = 77.16 ppm. ^{19}F NMR spectra are referenced based on the internal standard 4-fluoroanisole. ^{1}H and ^{19}F multiplicities are reported as follows: singlet (s), broad singlet (br), doublet (d), triplet (t), quartet (q), pentet (pent) multiplet (m), sextet (sext), septet (sept), doublet of doublets (dd), doublet of triplets (dt), triplet of doublets (td), doublet of doublet of doublets (ddd), doublet of doublet of triplets (ddt), triplet of quartet of doublets (tqd). Coupling constants (J) are reported in Hz.

2. Methods and Materials

All reactions reported herein were performed without the exclusion of moisture or air unless otherwise noted. No dry solvents were used in any reactions for the synthesis of the sulfone iminium fluoride reagent or in the fluorination reactions using SIFs.

Commercial reagents and solvents were used as received unless otherwise noted. Thiophenol, sulfuryl chloride, acetic acid, benzylamine, methyl trifluoromethanesulfonate, diphenylphosphinic acid, bis(4-methoxyphenyl)phosphinic acid, diethyl phosphate, bis(2-ethylhexyl)phosphate, dibenzyl phosphate, phenyl phosphonic acid and benzyl phosphonic acid, diisooctylphosphinic acid, 2-bromothiophene, *n*-butyl lithium, 4-fluorophenyl magnesium bromide, benzyl magnesium bromide, 2-mesityl magnesium bromide, dichlorophenylphosphine, *iso*-propylmagnesium bromide, 2-methylphenyl magnesium bromide, dichloro *tert*-butyl phosphine and phenylethynyl magnesium bromide were purchased from Sigma Aldrich. 4-bromoanisole, 1-bromonaphthalene, 2-bromopyridine, 4-bromotoluene, 3,5-dimethyl-1-bromobenzene, triethylamine, proton sponge, pyridine, 2,6-lutidine and DBU were purchased from Acros Organics. $CDCl_3$ was purchased from Cambridge Isotope Laboratories and used as is. Solvents were purchased Pharmco and used as is.

3. Synthesis of sulfone iminium fluoride reagent

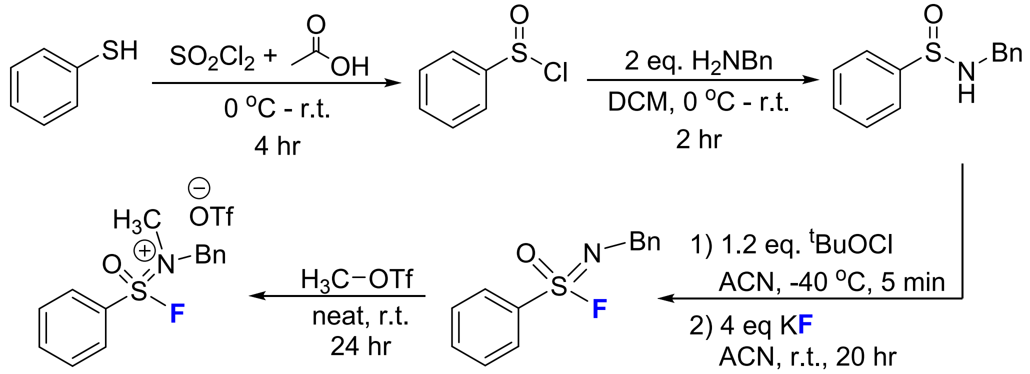

The following synthesis is modified from *Org. Lett.* **2022**, *24*, 5962.[1]

To a 100 mL round bottom flask, thiophenol (10.25 mL, 100 mmol) and acetic acid (5.75 mL, 100 mmol) were added with a stir bar and placed in an ice bath. Sulfuryl chloride (17.85 ml, 220 mmol) was added dropwise to the reaction at 0 °C. Vigorous bubbling was observed as well as a color change to orange-red. After the addition was complete, the reaction was stirred at room temperature for 4 hours, during which time bubbling continued. After 4 hours, acetyl chloride and excess sulfuryl chloride were removed under reduced pressure in a bath no higher than 10 °C. The product was isolated as a bright orange liquid and used without further purification.

To a 1000 mL round bottom flask, sulfinyl chloride (16.0 g, 0.10 mol) was added with a stir bar. DCM (400 mL) was added and the flask was placed in an ice bath. Once cooled, benzylamine (21.4 g, 0.20 mol) dissolved in DCM (100 mL) was added slowly to the reaction. An immediate white precipitate appeared along with a change to a colorless solution. The reaction was then stirred at room temperature for 2 hours. At this time, the reaction was filtered through a pad of celite followed by washing with H_2O (300 mL X 2). The organic layer was dried over Na_2SO_4, filtered and the solvent was removed under reduced pressure to yield a white solid (21.7 g, 94% yield). The sulfinamide product was used in subsequent reactions without any further purification.

To a 250 mL round bottom flask, *N*-benzyl benzenesulfinamide (6.94 g, 30 mmol) was added with acetonitrile (100 mL). The flask was then set in a dry ice / acetonitrile bath (-40 °C) and allowed to cool for 5 minutes. *Tert*-butyl hypochlorite (3.89 g, 36 mmol, 1.2 equivalents) was dissolved in acetonitrile (10 mL) and added to the reaction flask slowly over the course of 10 minutes. The reaction was allowed to slowly warm to room temperature at which time potassium fluoride (6.96 g, 120 mmol, 4 equivalents) was added to the reaction. The reaction was then stirred for 20 hours at room temperature. At this time, the reaction was filtered through a pad of celite and the solvent was removed under reduced pressure. The crude sulfonimidoyl fluoride product was purified by silica gel column chromatography using hexane / ethyl acetate (0% → 20% gradient, 40 g silica gel column) as eluent. Following purification, the *N*-benzyl benzenesulfonimidoyl fluoride was isolated as a pale yellow liquid (5.38 g, 72%).

In a well-ventilated fume hood under normal atmospheric conditions, *N*-benzyl benzenesulfonimidoyl fluoride (3.74 g, 15.0 mmol) was added with a flea stir bar to a 20 mL scintillation vial. Methyl trifluoromethanesulfonate (1.80 mL, 16.5 mmol. 1.1 equivalents) was added to the reaction at room temperature. The reaction was then stirred for 24 hours at room temperature. After this time, the crude reaction mixture was washed with hexane (10 mL, 5 times) in order to remove excess methyl trifluoromethanesulfonate. The sulfone iminium fluoride product (5.89 g, 98%) was then used without further purification.

Handling of SIF Reagent

Once synthesized, the SIF reagent is stored in a freezer at -10 °C for long term stability. For all reactions, the SIF reagent is weighed out in a well-ventilated fume hood where the reaction is then conducted as well.

4. Synthesis of phosphine oxide and phosphinic acid substrates

General procedure for the synthesis of symmetrical phosphine oxide:

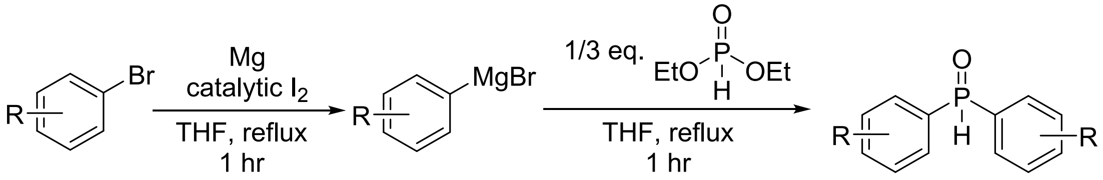

The following is a modified procedure from *Org. Lett.* **2022**, *24*, 6083.[2]

To a 100 mL round bottom flask, aryl bromide (30 mmol, 3 equivalents) was added along with Mg turnings (780 mg, 32 mmol), I_2 (catalytic) and THF (10 mL). The reaction was heated to reflux for 1 hour at which time, the reaction was cooled to room temperature and diethyl phosphite (1.38 g, 10 mmol, 1 equivalent) was added with THF (5 mL). The reaction was once again heated to reflux for one hour. After this time, the mixture was cooled to 0 °C and NH_4Cl (5 mL) was added to quench the reaction. The crude mixture was then extracted with chloroform and washed with water (3 x 100 mL). The organic layer was dried over Na_2SO_4 and the solvent was removed under reduced pressure. Purification was performed via silica gel chromatography using hexane / ethyl acetate as the eluent.

The following secondary phosphine oxides were synthesized through the above procedure. Spectroscopic data (^{1}H and ^{31}P NMR) matched those reported in the literature.[2]

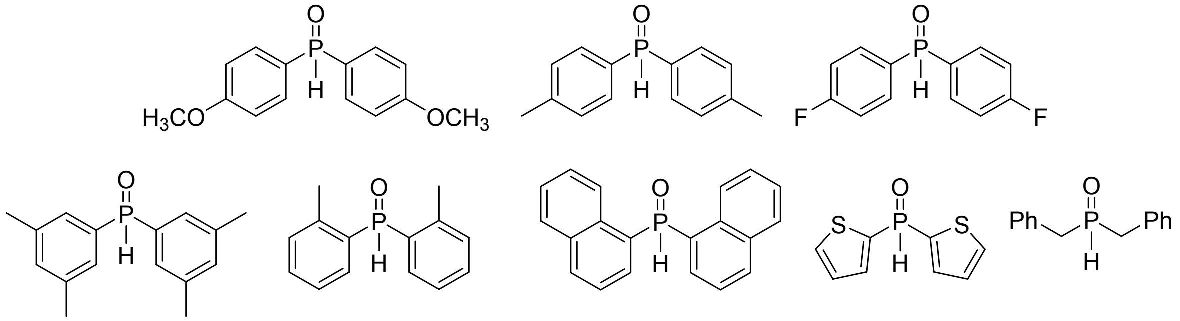

General procedure for the synthesis of unsymmetrical phosphine oxides:

Procedure A

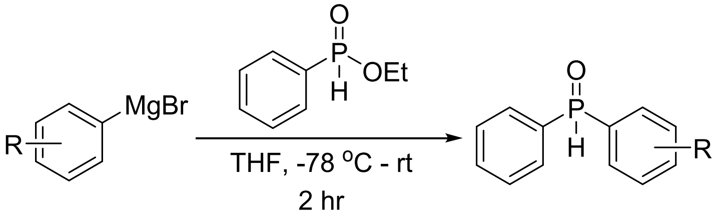

The following procedure is modified from *J. Am. Chem. Soc.* 2019, 141, 36, 14098–14103.[3]

To a 100 mL Schlenk flask, the appropriate Grignard (22.0 mmol, 2.2 equivalents) was added under an atmosphere of dinitrogen. The flask was then placed in a dry ice / acetone (-78 °C) bath. Ethyl phenylphosphinate (1.70 g, 10.0 mmol) was then dissolved in THF (5 mL) and added to the reaction flask slowly. The reaction was then warmed slowly to room temperature and stirred for 2 hours. After this time, the mixture was cooled to 0 °C and NH_4Cl (5 mL) was added to quench the reaction. The crude mixture was then extracted with chloroform and dried over Na_2SO_4 followed by purification via silica gel chromatography using hexane / ethyl acetate as the eluent.

The following secondary phosphine oxides were synthesized through the above procedure. Spectroscopic data (^1H and ^{31}P NMR) matched those reported in the literature.[3, 4]

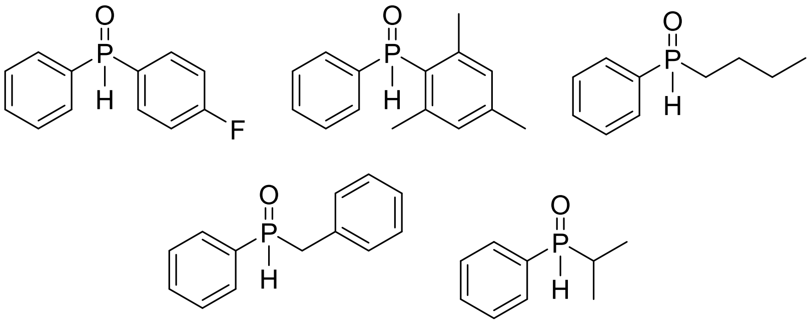

Procedure B

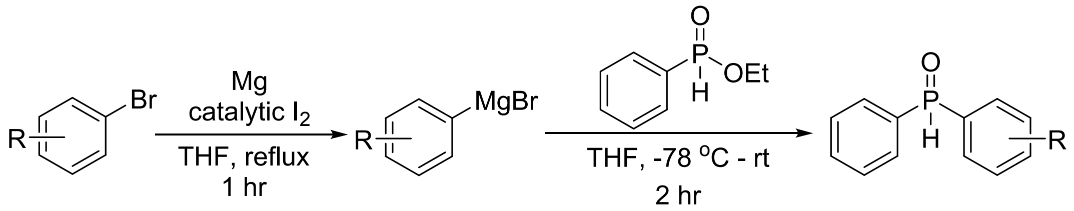

To a 100 mL round bottom flask, aryl bromide (22.0 mmol, 2.2 equivalents) was added along with Mg turnings (610 mg, 25 mmol), I_2 (catalytic) and THF (10 mL). The reaction was heated to reflux for 1 hour at which time, the reaction was cooled to room temperature and ethyl phenylphosphinate (1.70 g, 10 mmol) was added with THF (5 mL). The reaction was once again heated to reflux for one hour. After this time, the mixture was cooled to 0 ^{o}C and NH_4Cl (5 mL) was added to quench the reaction. The crude mixture was then extracted with chloroform and dried over Na_2SO_4 followed by purification via silica gel chromatography using hexane / ethyl acetate as the eluent.

The following secondary phosphine oxides were synthesized through the above procedure. Spectroscopic data (^{1}H and ^{31}P NMR) matched those reported in the literature.[5]

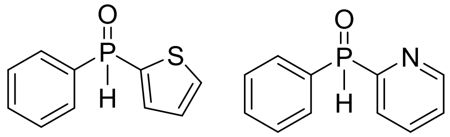

Procedure C

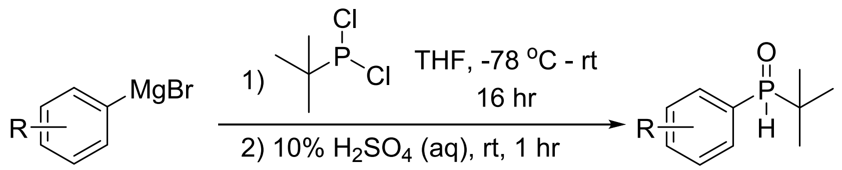

The following procedure is modified from *J. Am. Chem. Soc.* 2019, 141, 36, 14098–14103.[3]

A 100 mL Schlenk flask was charged with *tert*-butyldichlorophosphine (1.59 g, 10.0 mmol) and THF (10 mL). The solution was cooled to -78 °C and the appropriate Grignard reagent (10.0 mmol, 1 equiv.) was added slowly over a period of 15 minutes. The reaction was stirred at room temperature for 24 hours. The mixture was then cooled to 0 °C and quenched with 10% H_2SO_4 (aq) solution and then stirred for 1 hour at room temperature. The reaction was then diluted with water (50 mL) and extracted with $CHCl_3$ (3 x 50 mL). The combined organic layers were dried over Na_2SO_4 and the solvent was removed under reduced pressure. The secondary phosphine oxide products were used without any further purification.

The following secondary phosphine oxides were synthesized through the above procedure. Spectroscopic data (^{1}H and ^{31}P NMR) matched those reported in the literature.[3, 6]

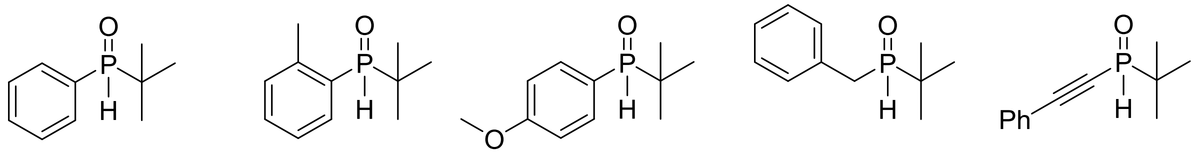

General procedure for the synthesis of phosphinic acid substrates:

The following is a modified procedure from *J. Am. Chem. Soc.* 2019, 141, 36, 14098–14103.[3]

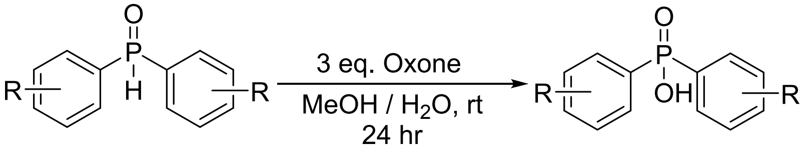

To a 100 mL round bottom flask, the appropriate phosphine oxide (10.0 mmol, 1 equivalent) was added along with methanol (20 mL). Oxone was dissolved in H_2O (20 mL) and then added to the flask. The slurry was then stirred for 24 hours at room temperature. At this time, the reaction mixture was placed in a separatory funnel and extracted into chloroform (50 mL x 3). The organic layer was washed with 1M NaOH (50 mL x 3) which was then acidified using concentrated HCl. The subsequent precipitate was extracted into chloroform (50 mL x 3) and the organic layer was dried over Na_2SO_4, filtered and the solvent was removed under reduced pressure. The phosphinic acid products were used without any further purification. Spectroscopic data (^{1}H and ^{31}P NMR) for phosphinic acid substrates matched with the literature.[7]

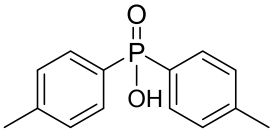
1c

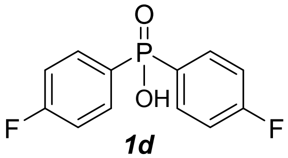
1d

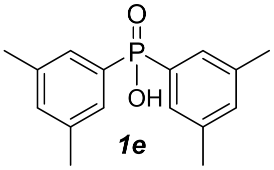
1e

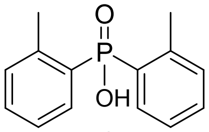
1f

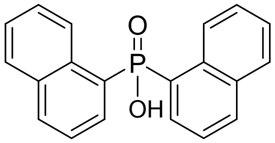
1g

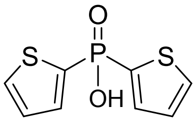
1h

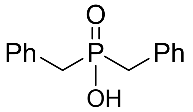
1j

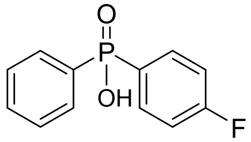
1k

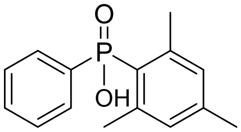
1l

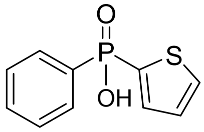
1m

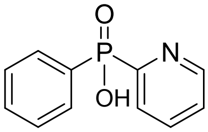
1n

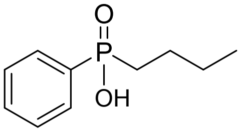
1p

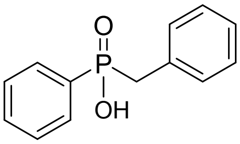
1q

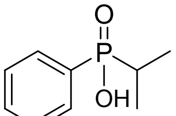
1r

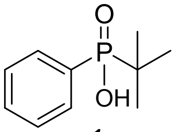
1s

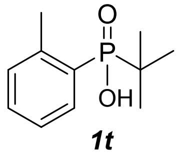
1t

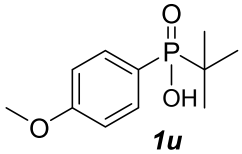
1u

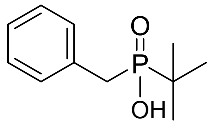
1v

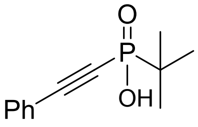
1w

5. Optimization of conditions for the deoxyfluorination of diphenylphosphinic acid

All reactions were performed on the benchtop without the exclusion of air or moisture. Reagents and solvents were used as received and without the need for drying.

Optimization of reaction solvent (Table 1, Entries 1 – 5)

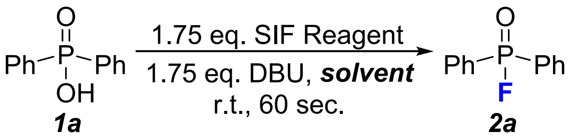

To an 8 mL vial, diphenylphosphinic acid (44.0 mg, 0.20 mmol), DBU (53.2 mg, 0.35 mmol) and 4-fluoroanisole (25.2 mg, 0.2 mmol) were added along with the appropriate solvent (0.75 mL). SIF reagent (140 mg, 0.35 mmol) was dissolved in the chosen solvent (0.25 mL) and added to the reaction vial at room temperature. The reaction was stirred for 60 seconds when it was transferred to an NMR tube and the yield was determined by ^{19}F NMR by comparison to the internal standard. The ^{19}F NMR spectra for Entries 1 – 5 from Table 1 are shown below.

Table 1, Entry 1: DCM (65% yield)

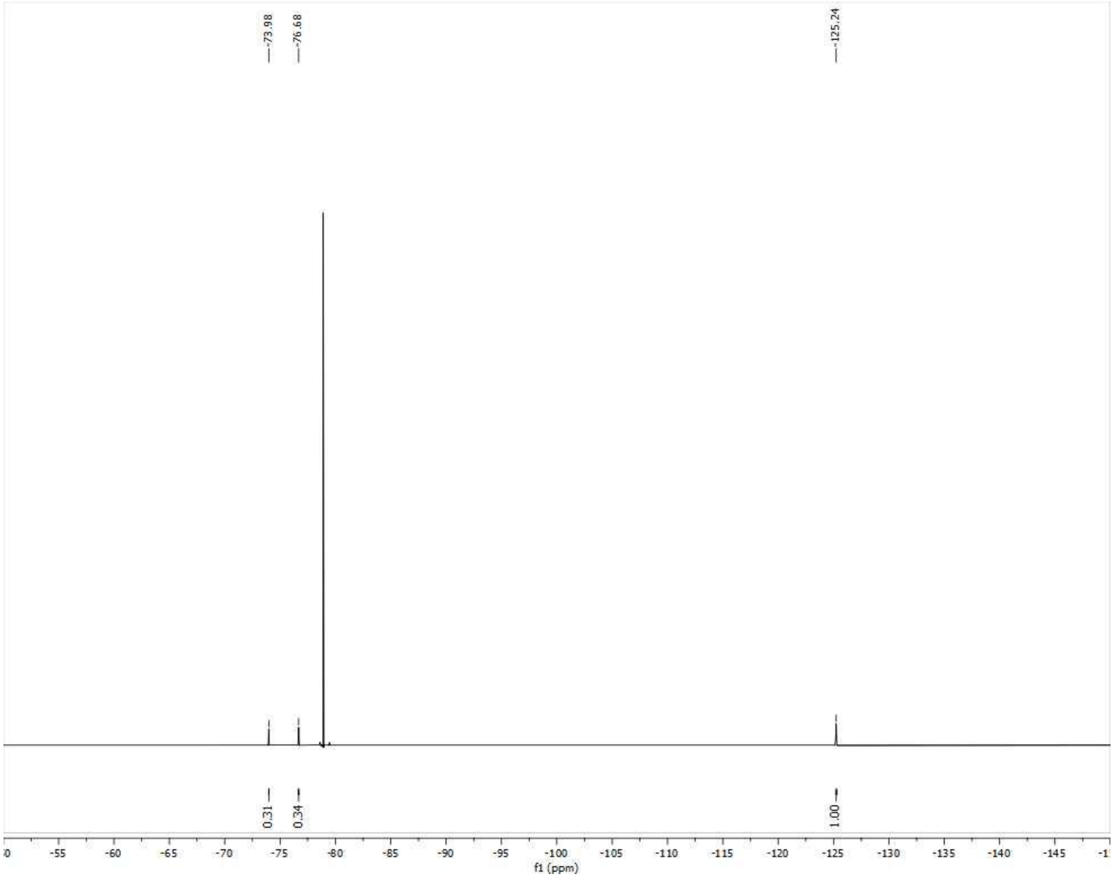

Table 1, Entry 2: Toluene (57% yield)

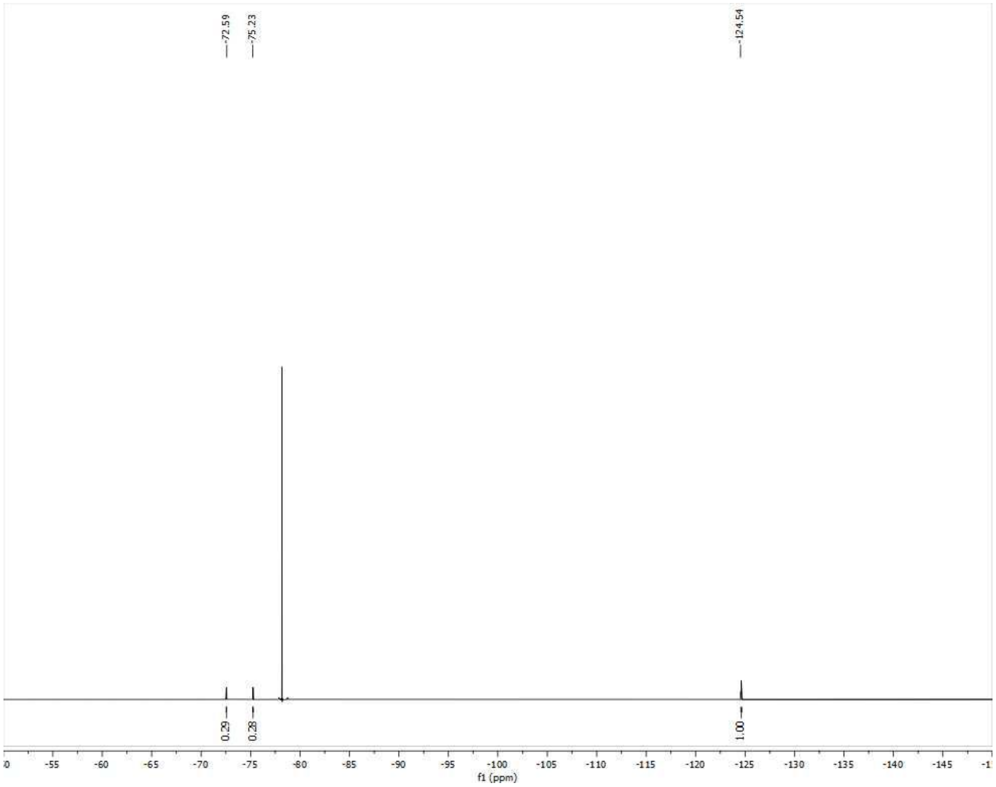

Table 1, Entry 3: Acetonitrile (74% yield)

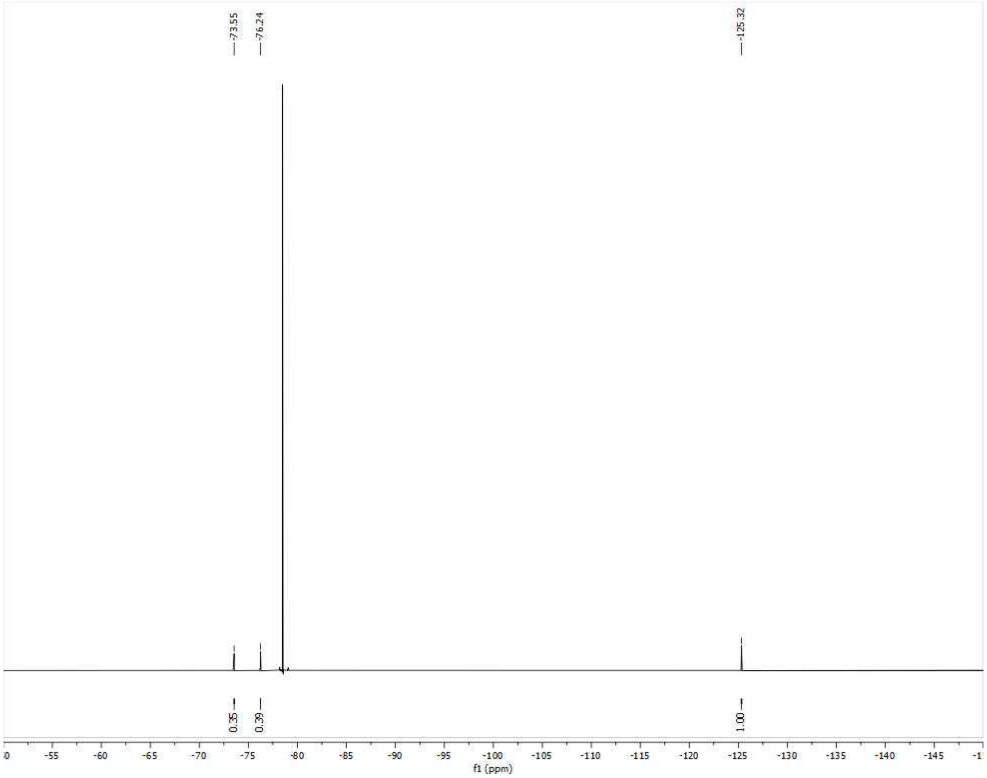

Table 1, Entry 4: DMF (0% yield)

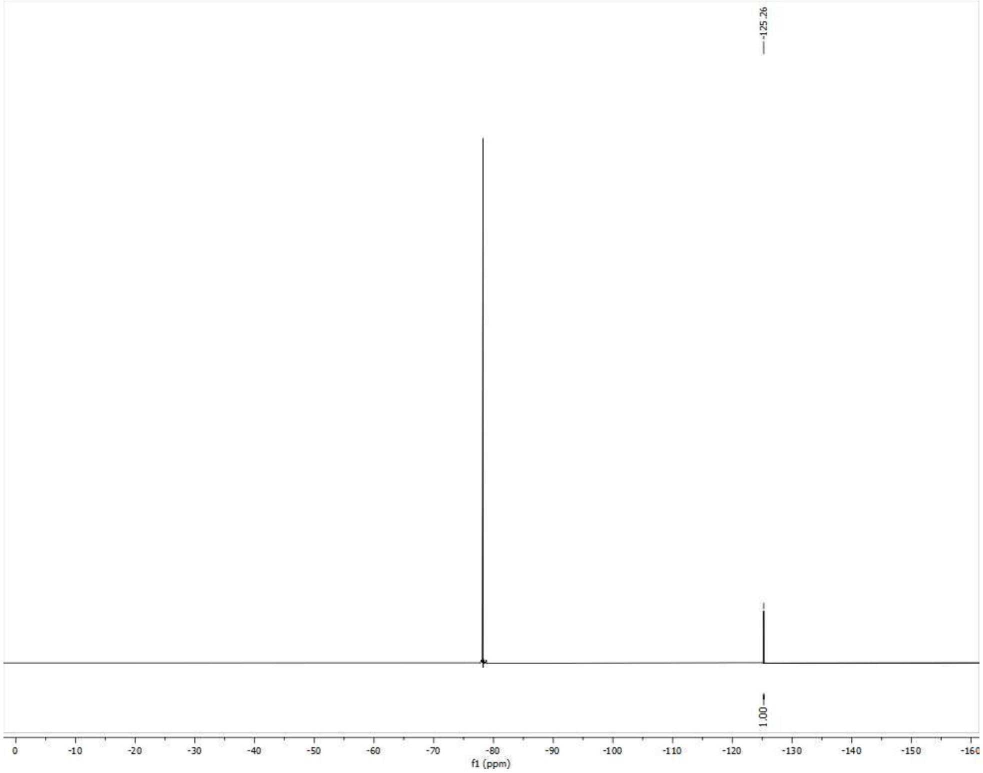

Table 1, Entry 5: THF (6% yield)

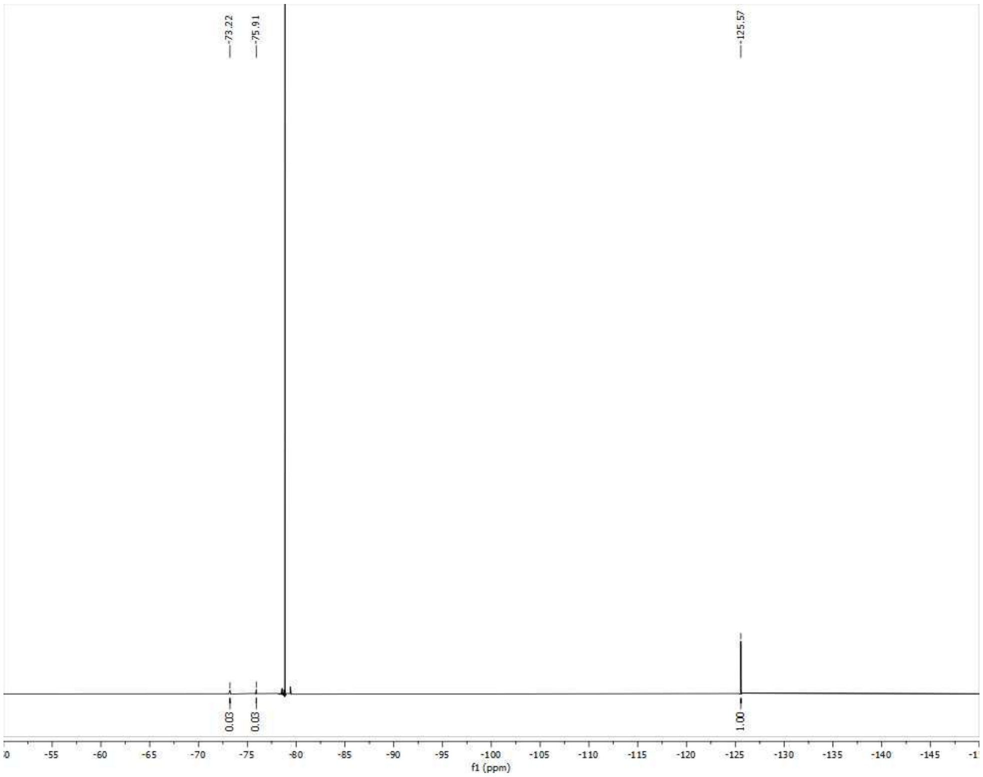

Optimization of base (Table 1, Entries 6 – 9)

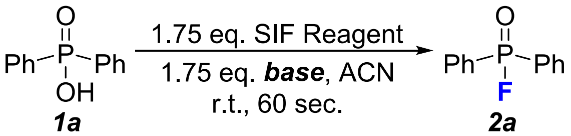

To an 8 mL vial, diphenylphosphinic acid (44.0 mg, 0.20 mmol), base (0.35 mmol) and 4-fluoroanisole (25.2 mg, 0.2 mmol) were added along with acetonitrile (0.75 mL). SIF reagent (140 mg, 0.35 mmol) was dissolved (0.25 mL) and added to the reaction vial at room temperature. The reaction was stirred for 60 seconds when it was transferred to an NMR tube and the yield was determined by ^{19}F NMR by comparison to the internal standard. The ^{19}F NMR spectra for Entries 6 – 9 from Table 1 are shown below.

Table 1, Entry 6: pyridine (63% yield)

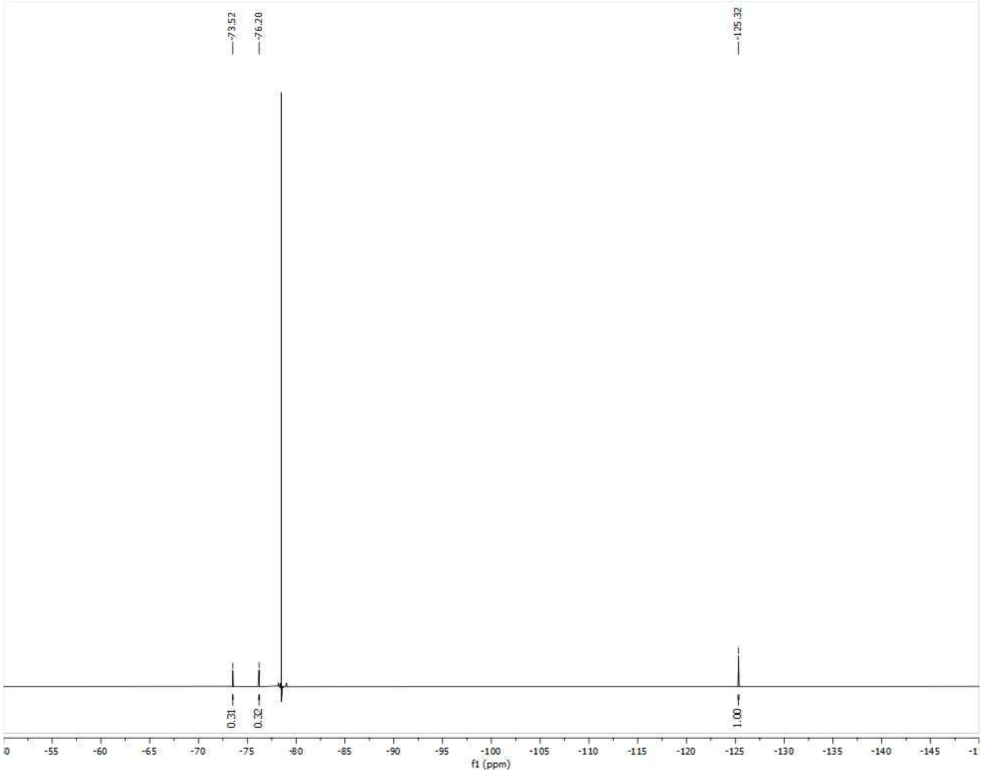

Table 1, Entry 7: 2,6-lutidine (36% yield)

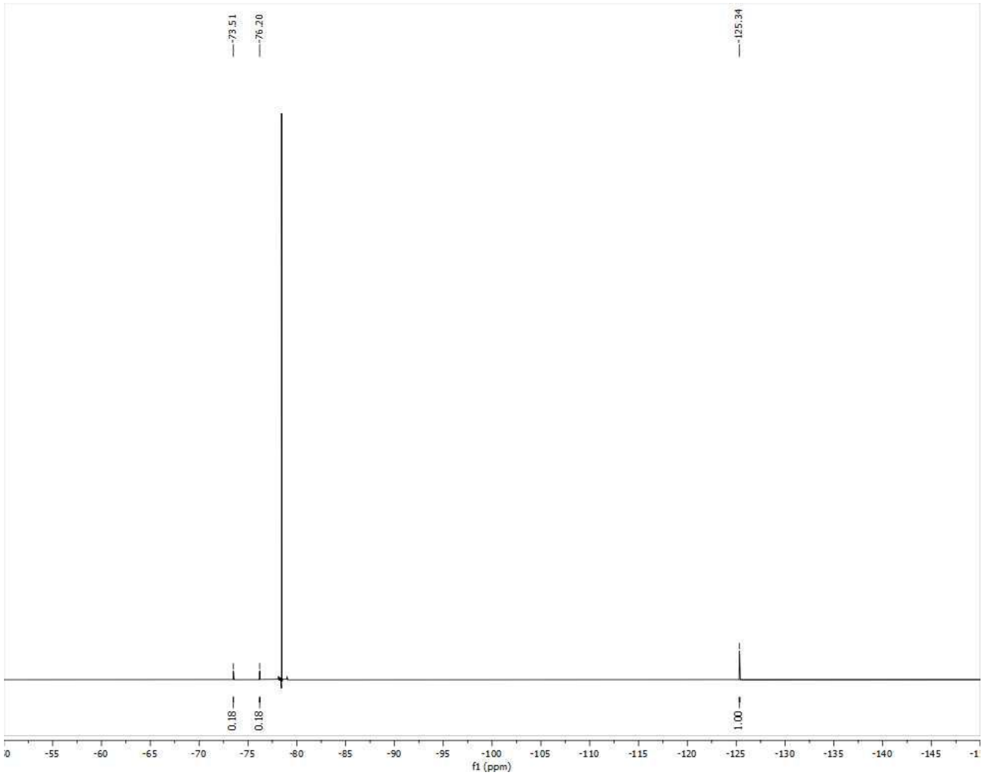

Table 1, Entry 8: triethylamine (88% yield)

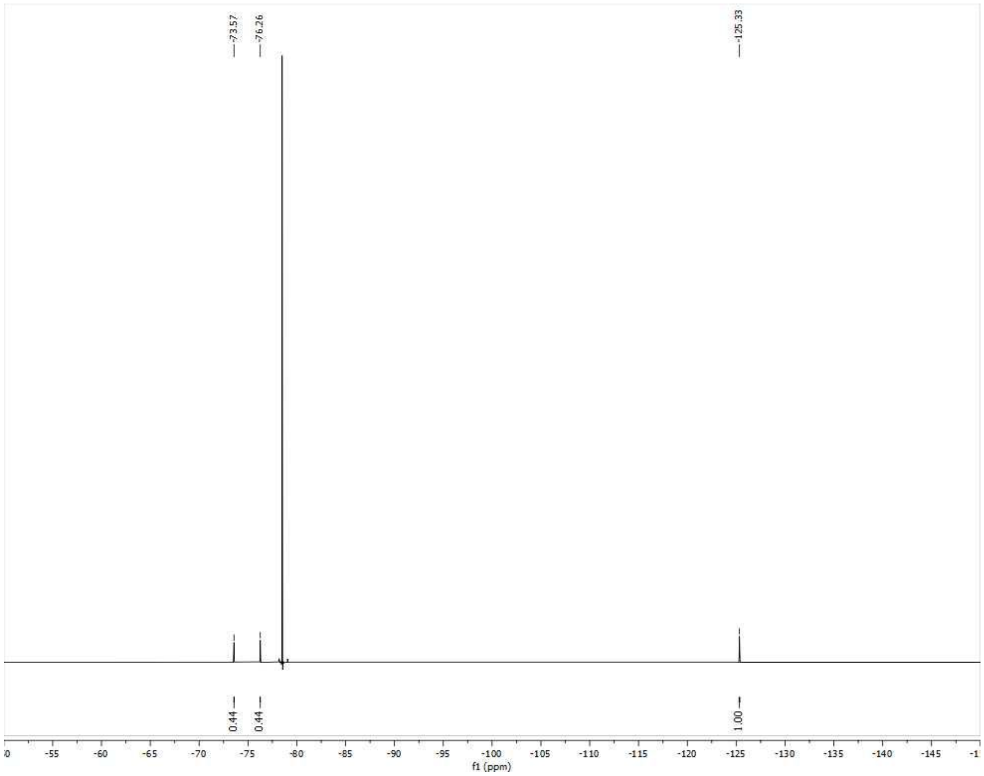

Table 1, Entry 9: proton sponge (85% yield)

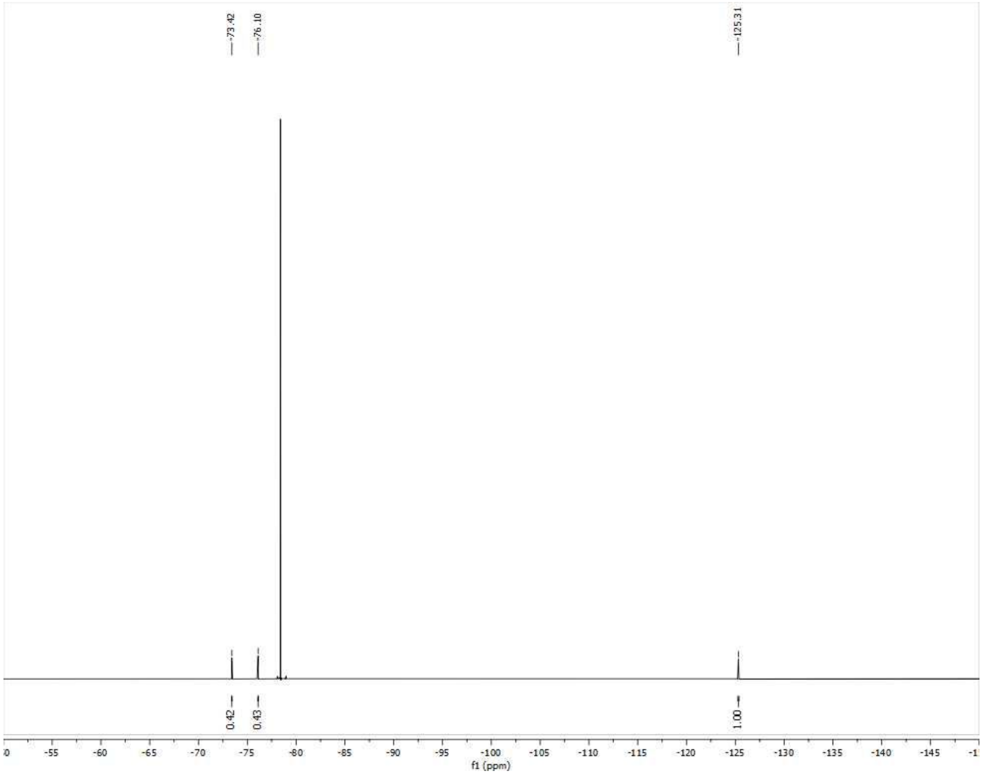

Optimization of SIF and base equivalents (Table 1, Entry 10)

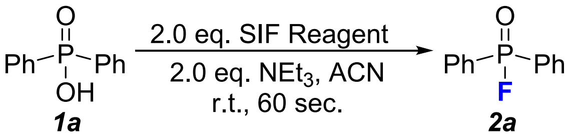

To an 8 mL vial, diphenylphosphinic acid (44.0 mg, 0.20 mmol), NEt_3 (40.4 mg 0.40 mmol) and 4-fluoroanisole (25.2 mg, 0.2 mmol) were added along with acetonitrile (0.75 mL). SIF reagent (160 mg, 0.40 mmol) was dissolved (0.25 mL) and added to the reaction vial at room temperature. The reaction was stirred for 60 seconds when it was transferred to an NMR tube and the yield was determined by ^{19}F NMR by comparison to the internal standard. The ^{19}F NMR spectrum for Entry 10 from Table 1 is shown below.

Table 1, Entry 10: 2 equivalents of SIF and NEt_3 (99% yield)

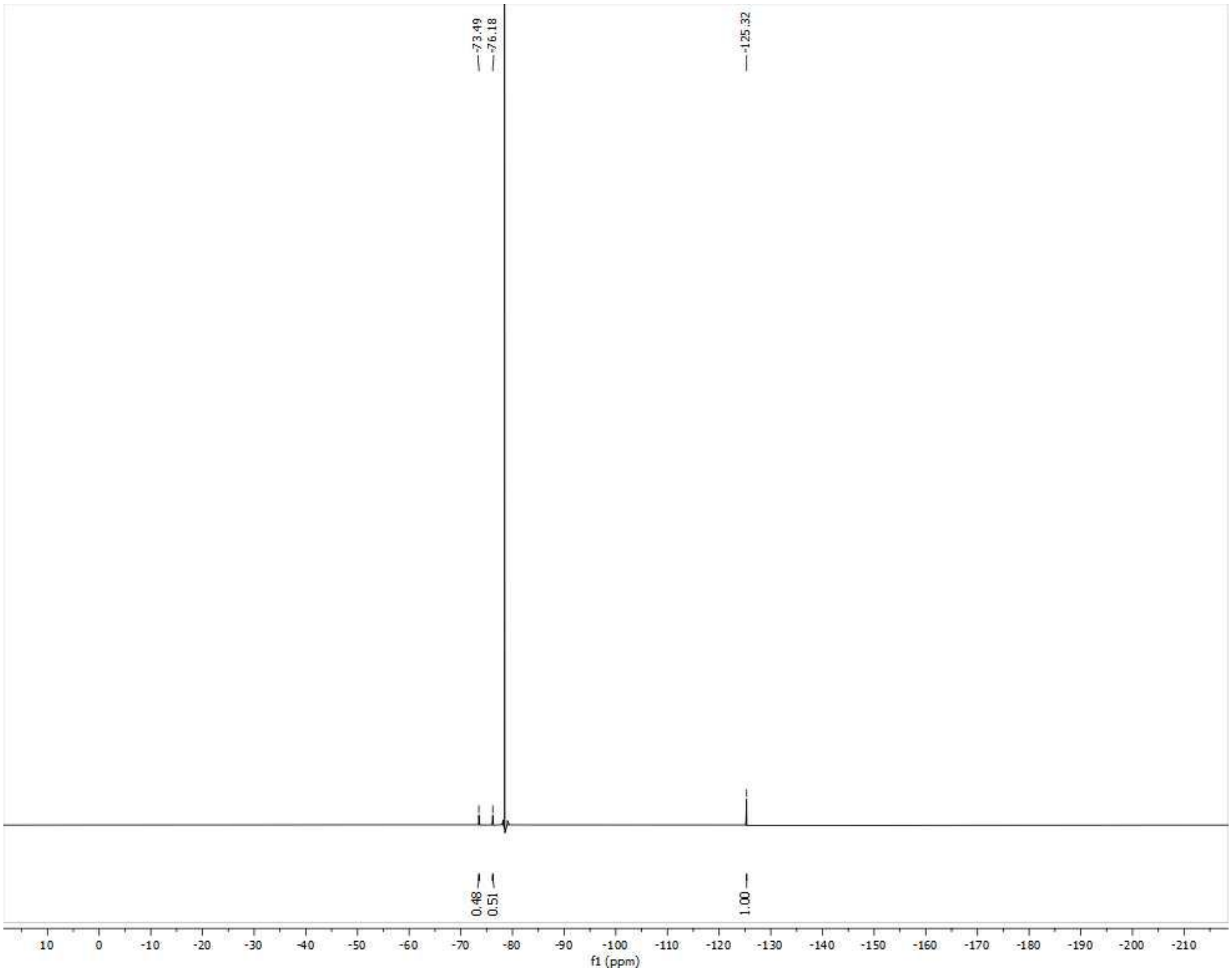

6. Screening of other S(VI) reagents for phosphinic acid deoxyfluorination

PyFluor and SulfoxFluor with our optimized conditions

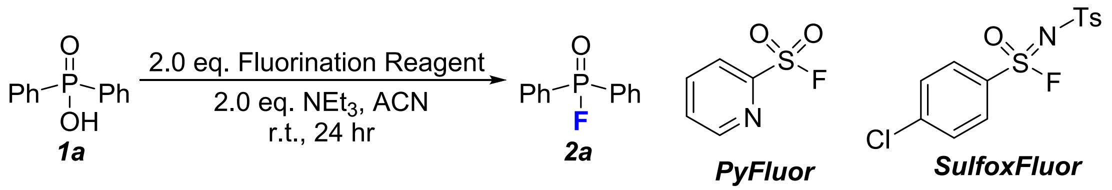

To an 8 mL vial, diphenylphosphinic acid (44.0 mg, 0.20 mmol), NEt_3 (40.4 mg 0.40 mmol) and 4-fluoroanisole (25.2 mg, 0.2 mmol) were added along with acetonitrile (0.75 mL). PyFluor (64.5 mg, 0.40 mmol) or SulfoxFluor (139 mg, 0.40 mmol) was dissolved (0.25 mL) and added to the reaction vial at room temperature. The reaction was stirred for 24 hours when it was transferred to an NMR tube and the yield was determined by ^{19}F and ^{31}P NMR. No product peak was observed in the ^{19}F or ^{31}P NMR spectra in either case. The ^{31}P NMR spectrum of each reaction is shown below which demonstrates only starting material observed and no product (doublet at 45 ppm).

Reaction with PyFluor and fluorination reagent:

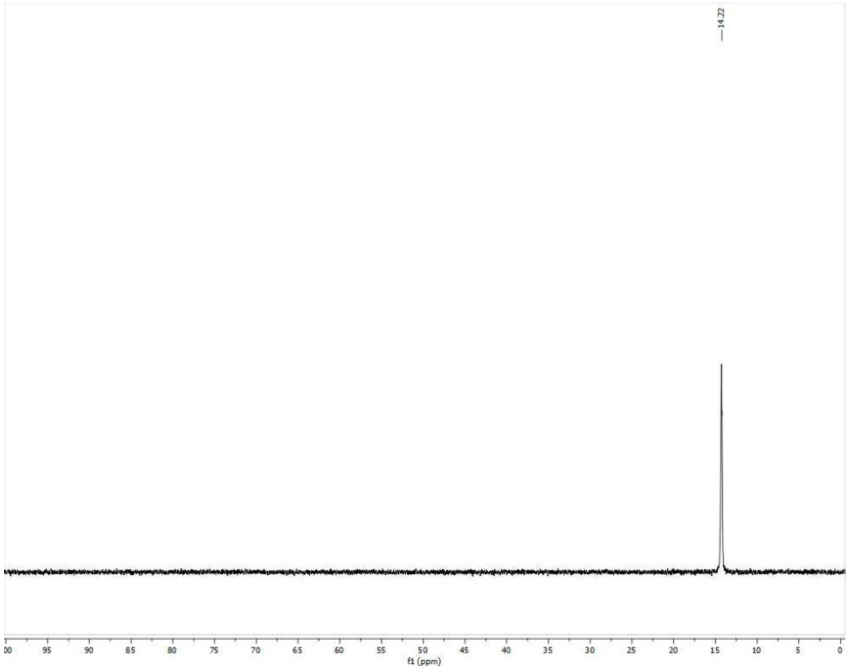

Reaction with SulfoxFluor as fluorinating reagent

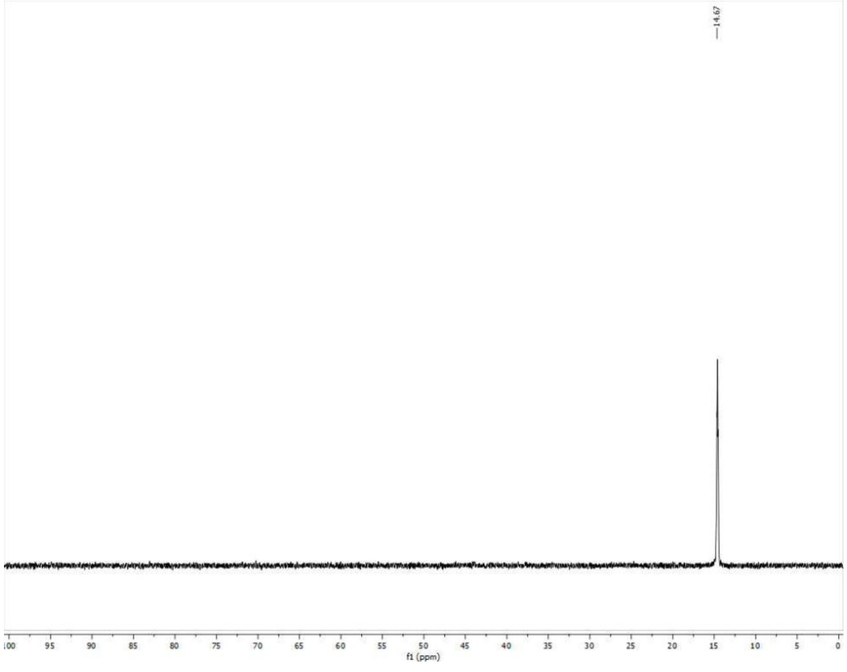

PyFluor and SulfoxFluor with previously optimized conditions[8]

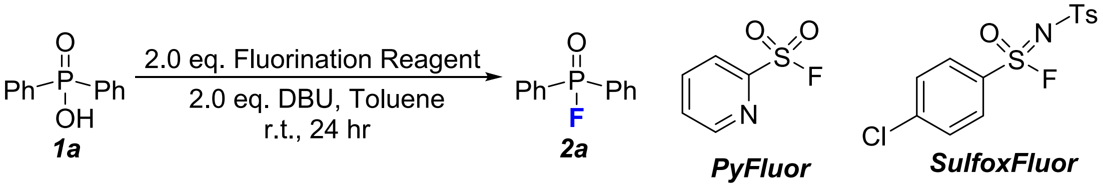

To an 8 mL vial, diphenylphosphinic acid (44.0 mg, 0.20 mmol), DBU (60.9 mg 0.40 mmol) and 4-fluoroanisole (25.2 mg, 0.2 mmol) were added along with toluene (0.75 mL). PyFluor (64.5 mg, 0.40 mmol) or SulfoxFluor (139 mg, 0.40 mmol) was dissolved (0.25 mL) and added to the reaction vial at room temperature. The reaction was stirred for 24 hours when it was transferred to an NMR tube and the yield was determined by ^{19}F and ^{31}P NMR. No product peak was observed in the ^{19}F or ^{31}P NMR spectra in either case.

7. Deoxyfluorination of phosphinic acids, phosphates and phosphonic acids

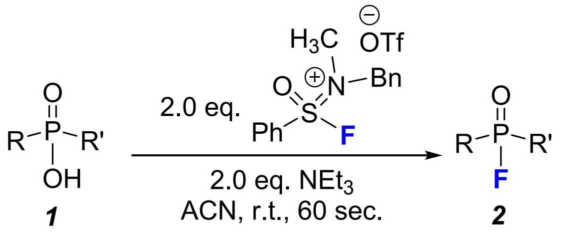

All reactions were performed on the benchtop without the exclusion of air or moisture. Reagents and solvents were used as received and without the need for drying.

To an 8 mL vial, phosphinic acid (0.50 mmol) and NEt_3 (101 mg 1.0 mmol) were added along with acetonitrile (1.5 mL). SIF reagent (401 mg, 1.0 mmol) was dissolved (0.5 mL) and added to the reaction vial at room temperature. The reaction was stirred for 60 seconds at which time the solvent was removed under reduced pressure. The crude reaction mixture was then purified via silica gel chromatography using hexane and ethyl acetate as eluent (0 → 50% gradient, 12-gram silica gel column).

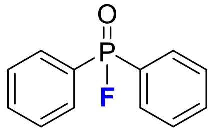

Diphenylphosphinic fluoride (2a). The reaction was performed using the standard conditions described above with diphenylphosphinic acid (436.4 mg, 2.0 mmol). Following silica gel chromatography, product **2a** was obtained as a colorless oil (422.8 mg, 96%). The isolated yield reported in the manuscript is the average of two runs (97% and 96% yield). The ^{1}H, ^{13}C, ^{19}F and ^{31}P NMR spectra were consistent with those published in the literature.[7]

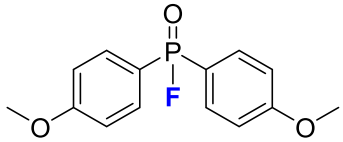

Bis(4-methoxyphenyl)phosphinic fluoride (2b). The reaction was performed using the standard conditions described above with bis(4-methoxyphenyl)phosphinic acid (139.1 mg, 0.5 mmol). Following silica gel chromatography, product **2b** was obtained as a white solid (134.0 mg, 95%). The isolated yield reported in the manuscript is the average of two runs (95% and 93% yield). The ^{1}H, ^{13}C, ^{19}F and ^{31}P NMR spectra were consistent with those published in the literature.[7]

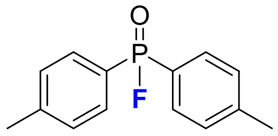

Bis(4-methylphenyl)phosphinic fluoride (2c). The reaction was performed using the standard conditions described above with bis(4-methylphenyl)phosphinic acid (123.1 mg, 0.5 mmol). Following silica gel chromatography, product **2c** was obtained as a white solid (121.3 mg, 97%). The isolated yield reported in

the manuscript is the average of two runs (97% and 97% yield). The ^{1}H, ^{13}C, ^{19}F and ^{31}P NMR spectra were consistent with those published in the literature.[7]

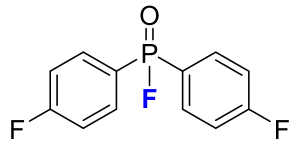

Bis(4-fluorophenyl)phosphinic fluoride (2d). The reaction was performed using the standard conditions described above with bis(4-fluorophenyl)phosphinic acid (127.1 mg, 0.5 mmol). Following silica gel chromatography, product **2d** was obtained as a colorless oil (122.6 mg, 95%). The isolated yield reported in the manuscript is the average of two runs (95% and 91% yield). The ^{1}H, ^{13}C, ^{19}F and ^{31}P NMR spectra were consistent with those published in the literature.[7]

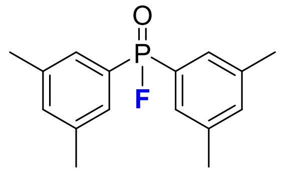

Bis(3,5-dimethylphenyl)phosphinic fluoride (2e). The reaction was performed using the standard conditions described above with bis(3,5-dimethylphenyl)phosphinic acid (137.1 mg, 0.5 mmol). Following silica gel chromatography, product **2e** was obtained as a white solid (128.0 mg, 92%). The isolated yield reported in the manuscript is the average of two runs (92% and 90% yield). The ^{1}H, ^{13}C, ^{19}F and ^{31}P NMR spectra were consistent with those published in the literature.[7]

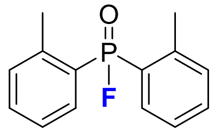

Bis(2-methylphenyl)phosphinic fluoride (2f). The reaction was performed using the standard conditions described above with bis(2-methylphenyl)phosphinic acid (123.1 mg, 0.5 mmol). Following silica gel chromatography, product **2f** was obtained as a colorless oil (120.1 mg, 96%). The isolated yield reported in the manuscript is the average of two runs (96% and 96% yield). The ^{1}H, ^{13}C, ^{19}F and ^{31}P NMR spectra were consistent with those published in the literature.[9]

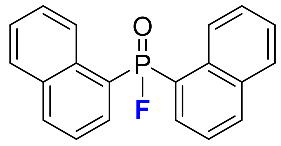

Bis(2-naphthyl)phosphinic fluoride (2g). The reaction was performed using the standard conditions described above with bis(2-naphthyl)phosphinic acid (159.1 mg, 0.5 mmol). Following silica gel

chromatography, product **2g** was obtained as a white solid (151.5 mg, 94%). The isolated yield reported in the manuscript is the average of two runs (94% and 91% yield). The ^{1}H, ^{13}C, ^{19}F and ^{31}P NMR spectra were consistent with those published in the literature.[7]

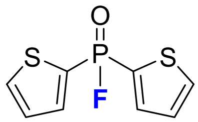

Bis(2-thienyl)phosphinic fluoride (2h). The reaction was performed using the standard conditions described above with bis(2-thienyl)phosphinic acid (115.1 mg, 0.5 mmol). Following the completion of the 60 second reaction time, 4-fluoroanisole (63.0 mg, 0.5 mmol, 1.0 eq.) was added to the reaction. The crude product yield was then determined by ^{19}F NMR spectroscopy by comparison of the internal standard signal to the product signal. The ^{19}F NMR yield reported in the manuscript is the average of two runs (93% and 89%).

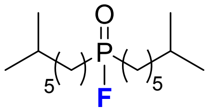

Bis(isooctyl)phosphinic fluoride (2i). The reaction was performed using the standard conditions described above with diisooctylphosphinic acid (117.2 mg, 0.5 mmol). Following the completion of the 60 second reaction time, 4-fluoroanisole (63.0 mg, 0.5 mmol, 1.0 eq.) was added to the reaction. The crude product yield was then determined by ^{19}F NMR spectroscopy by comparison of the internal standard signal to the product signal. The ^{19}F NMR yield reported in the manuscript is the average of two runs (96% and 92%). The ^{19}F and ^{31}P NMR signals were consistent with that published in the literature.[7]

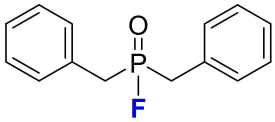

Bis(benzyl)phosphinic fluoride (2j). The reaction was performed using the standard conditions described above with dibenzylphosphinic acid (123.1 mg, 0.5 mmol). Following silica gel chromatography, product **2j** was obtained as a white solid (113.9 mg, 91%). The isolated yield reported in the manuscript is the average of two runs (91% and 89% yield). The ^{1}H, ^{13}C, ^{19}F and ^{31}P NMR spectra were consistent with those published in the literature.[7]

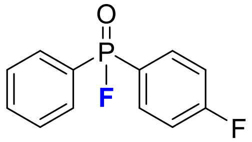

(4-fluorophenyl)(phenyl)phosphinic fluoride (2k). The reaction was performed using the standard conditions described above with (4-fluorophenyl)(phenyl)phosphinic acid (118.1 mg, 0.5 mmol). Following silica gel chromatography, product **2k** was obtained as a colorless oil (106.9 mg, 89%). The isolated yield reported in the manuscript is the average of two runs (89% and 86% yield).

^{1}H NMR (400 MHz, CDCl$_3$): 7.77 – 7.87 (m, 4H), 7.61 (t, J = 7.5 Hz, 1H), 7.48 – 7.53 (m, 2H), 7.16 – 7.21 (m, 2H) ppm.

^{13}C NMR (101 MHz, CDCl$_3$): 166.0 (dd, J = 255.7, 3.6 Hz), 134.3 (ddd, J = 13.0, 9.2, 1.9 Hz), 133.7 (d, J = 2.9 Hz), 131.5 (dd, J = 11.4, 2.1 Hz), 129.1 (d, J = 14.1 Hz), 128.6 (d, J = 120.4, 22.5 Hz), 124.9 (ddd, J = 145.0, 23.3, 3.5 Hz), 116.5 (dd, J = 21.7, 15.3 Hz) ppm.

^{31}P NMR (161 MHz, CDCl$_3$): 39.84 (d, $J_{F\text{-}P}$ = 1019.2 Hz) ppm.

^{19}F NMR (376 MHz, CDCl$_3$): -74.04 (d, $J_{P\text{-}F}$ = 1019.0 Hz, 1F), -103.63 (m, 1F) ppm.

HRMS ESI (m/z): [M + H] cald for $C_{12}H_{10}F_2OP$: 239.0437; found 239.0443

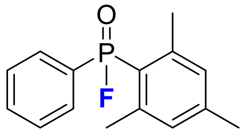

(2,4,6-trimethylphenyl)(phenyl)phosphinic fluoride (2l). The reaction was performed using the standard conditions described above with (2,4,6-trimethylphenyl)(phenyl)phosphinic acid (130.1 mg, 0.5 mmol). Following silica gel chromatography, product **2l** was obtained as a colorless oil (120.2 mg, 91%). The isolated yield reported in the manuscript is the average of two runs (91% and 91% yield).

^{1}H NMR (400 MHz, CDCl$_3$): 7.70 (ddt, J = 13.8, 6.9, 1.4 Hz, 2H), 7.58 (t, J = 9.1 Hz, 1H), 7.44 – 7.48 (m, 2H), 6.94 (d, J = 4.5 Hz, 2H), 2.48 (d, J = 4.1 Hz, 6H), 2.32 (s, 3H). ppm.

^{13}C NMR (101 MHz, CDCl$_3$): 143.6 (d, J = 2.9 Hz), 143.4 (d, J = 12.2 Hz), 133.0 (d, J = 2.9 Hz), 131.7 (dd, J = 115.0, 21.6 Hz), 130.9 (dd, 12.3, 0.6 Hz), 130.6 (dd, J = 12.2, 1.5 Hz), 128.9 (d, J = 14.0 Hz), 122.0 (dd, J = 137.0, 16.6 Hz), 23.2 (dd, J = 5.4, 4.0 Hz), 21.4 (d, J = 1.6 Hz). ppm.

^{31}P NMR (161 MHz, CDCl$_3$): 45.07 (d, $J_{F\text{-}P}$ = 1021.8 Hz) ppm.

^{19}F NMR (376 MHz, CDCl$_3$): -61.78 (d, $J_{P\text{-}F}$ = 1021.8 Hz) ppm.

HRMS ESI (m/z): [M + H] cald for $C_{15}H_{17}FOP$: 263.1001; found 263.0988

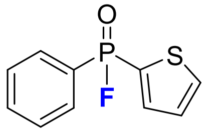

(2-thienyl)(phenyl)phosphinic fluoride (2m). The reaction was performed using the standard conditions described above with (2-thienyl)(phenyl)phosphinic acid (112.1 mg, 0.5 mmol). Following the completion of the 60 second reaction time, 4-fluoroanisole (63.0 mg, 0.5 mmol, 1.0 eq.) was added to the reaction. The crude product yield was then determined by ^{19}F NMR spectroscopy by comparison of the internal standard signal to the product signal. The ^{19}F NMR yield reported in the manuscript is the average of two runs (89% and 83%).

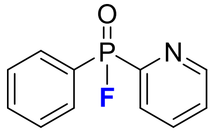

(2-pyridyl)(phenyl)phosphinic fluoride (2n). The reaction was performed using the standard conditions described above with (2-pyridyl)(phenyl)phosphinic acid (109.6 mg, 0.5 mmol). Following silica gel chromatography, product **2n** was obtained as a pale yellow oil (99.5 mg, 90%). The isolated yield reported in the manuscript is the average of two runs (90% and 89% yield).

^{1}H NMR (400 MHz, CDCl$_3$): 8.74 (d, *J* = Hz, 1H), 8.09 (t, *J* = 7.7 Hz, 1H), 7.97 (dd, *J* = 12.8, 8.2 Hz, 2H), 7.80 (q, *J* = 7.2 Hz, 1H), 7.56 (t, *J* = 7.5 Hz, 1H), 7.38 – 7.48 (m, 3H) ppm.

^{13}C NMR (101 MHz, CDCl$_3$): 152.4 (dd, *J* = 177.0, 25.7 Hz), 150.9 (d, *J* = 22.1 Hz), 136.5 (d, *J* = 11.1 Hz), 133.8 (d, *J* = 3.0 Hz), 132.4 (dd, *J* = 11.0, 1.9 Hz), 128.8 (d, *J* = 14.1 Hz), 128.3 (d, *J* = 1.8 Hz), 128.05 (d, *J* = 1.7 Hz), 126.9 (d, *J* = 3.8 Hz) ppm.

^{31}P NMR (161 MHz, CDCl$_3$): 32.66 (d, $J_{F\text{-}P}$ = 1040.4 Hz) ppm.

^{19}F NMR (376 MHz, CDCl$_3$): -76.74 (d, $J_{P\text{-}F}$ = 1039.9 Hz) ppm.

HRMS ESI (m/z): [M + H] cald for $C_{11}H_{10}FNOP$: 222.0484; found 222.0477

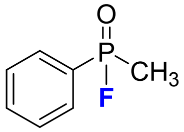

(methyl)(phenyl)phosphinic fluoride (2o). The reaction was performed using the standard conditions described above with (methyl)(phenyl)phosphinic acid (78.0 mg, 0.5 mmol). Following silica gel chromatography, product **2o** was obtained as a colorless oil (70.3 mg, 89%). The isolated yield reported in the manuscript is the average of two runs (89% and 85% yield). The ^{1}H, ^{13}C, ^{19}F and ^{31}P NMR spectra were consistent with those published in the literature.[10]

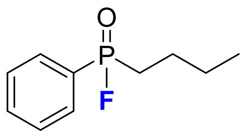

(*n*-butyl)(phenyl)phosphinic fluoride (2p). The reaction was performed using the standard conditions described above with (*n*-butyl)(phenyl)phosphinic acid (99.1 mg, 0.5 mmol). Following silica gel chromatography, product **2p** was obtained as a colorless oil (94.1 mg, 94%). The isolated yield reported in the manuscript is the average of two runs (94% and 92% yield). The ^{1}H, ^{13}C, ^{19}F and ^{31}P NMR spectra were consistent with those published in the literature.[9]

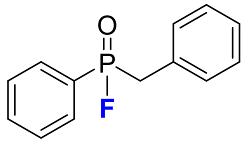

(benzyl)(phenyl)phosphinic fluoride (2q). The reaction was performed using the standard conditions described above with (benzyl)(phenyl)phosphinic acid (116.1 mg, 0.5 mmol). Following silica gel chromatography, product **2q** was obtained as a white solid (109.0 mg, 93%). The isolated yield reported in the manuscript is the average of two runs (93% and 92% yield). The ^{1}H, ^{13}C, ^{19}F and ^{31}P NMR spectra were consistent with those published in the literature.[11]

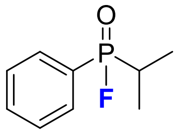

(*iso*-propyl)(phenyl)phosphinic fluoride (2r). The reaction was performed using the standard conditions described above with (*iso*-propyl)(phenyl)phosphinic acid (92.1 mg, 0.5 mmol). Following silica gel chromatography, product **2r** was obtained as a colorless oil (89.4 mg, 96%). The isolated yield reported in the manuscript is the average of two runs (96% and 92% yield).

^{1}H NMR (400 MHz, CDCl$_3$): 7.77 – 7.81 (m, 2H), 7.63 (t, J = 5.9 Hz, 1H), 7.50 – 7.55 (m, 2H), 2.24 (tqd, J = 14.3, 7.1, 2.7 Hz, 1H), 1.26 (dd, J = 17.9, 7.2 Hz, 3H), 1.16 (dd, J = 19.0, 7.2 Hz, 3H) ppm.

^{13}C NMR (101 MHz, CDCl$_3$): 133.48 (d, J = 2.8 Hz), 131.79 (dd, J = 10.4, 2.3 Hz), 128.91 (d, J = 12.9 Hz), 127.15 (dd, J = 126.2, 19.1 Hz), 28.27 (dd, J = 97.0, 16.6 Hz), 15.02 (d, J = 3.8 Hz) ppm.

^{31}P NMR (161 MHz, CDCl$_3$): 59.25 (d, $J_{F\text{-}P}$ = 1036.1 Hz) ppm.

^{19}F NMR (376 MHz, CDCl$_3$): -85.83 (d, $J_{P\text{-}F}$ = 1036.2 Hz) ppm.

HRMS ESI (m/z): [M + H] cald for $C_9H_{13}FOP$: 187.0688; found 187.0695

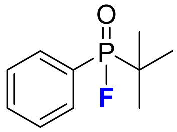

(*tert*-butyl)(phenyl)phosphinic fluoride (2s). The reaction was performed using the standard conditions described above with (*tert*-butyl)(phenyl)phosphinic acid (99.1 mg, 0.5 mmol). Following silica gel chromatography, product **2s** was obtained as a colorless oil (94.1 mg, 94%). The isolated yield reported in the manuscript is the average of two runs (94% and 93% yield). The ^{1}H, ^{13}C, ^{19}F and ^{31}P NMR spectra were consistent with those published in the literature.[12]

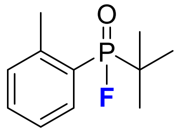

(*tert*-butyl)(2-methylphenyl)phosphinic fluoride (2t). The reaction was performed using the standard conditions described above with (*tert*-butyl)(2-methylphenyl)phosphinic acid (106.1 mg, 0.5 mmol). Following silica gel chromatography, product **2t** was obtained as a colorless oil (99.6 mg, 93%). The isolated yield reported in the manuscript is the average of two runs (93% and 87% yield).

^{1}H NMR (400 MHz, CDCl$_3$): 7.62 – 7.66 (m, 1H), 7.47 (t, *J* = 7.5 Hz, 1H), 7.27 – 7.32 (m, 2H), 2.68 (s, 3H), 1.21 (dd, *J* = 16.7, 1.0 Hz, 9H) ppm.

^{13}C NMR (101 MHz, CDCl$_3$): 143.4 (dd, *J* = 11.9, 2.6 Hz), 133.8 (dd, *J* = 8.9, 5.0 Hz), 133.1 (d, *J* = 2.8 Hz), 132.0 (dd, *J* = 13.2, 1.9 Hz), 126.8 – 129.3 (m), 125.4 (d, *J* = 11.8 Hz), 124.3 (dd, *J* = 117.9, 15.6 Hz), 34.2 (dd, *J* = 95.0, 15.9 Hz), 24.1 ppm.

^{31}P NMR (161 MHz, CDCl$_3$): 62.24 (d, $J_{F\text{-}P}$ = 1052.6 Hz) ppm.

^{19}F NMR (376 MHz, CDCl$_3$): -88.77 (d, $J_{P\text{-}F}$ = 1051.9 Hz) ppm.

HRMS ESI (m/z): [M + H] cald for $C_{11}H_{17}FOP$: 215.1001; found 215.0993

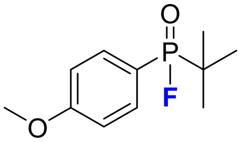

(*tert*-butyl)(4-methoxyphenyl)phosphinic fluoride (2u). The reaction was performed using the standard conditions described above with (*tert*-butyl)(4-methoxyphenyl)phosphinic acid (114.1 mg, 0.5 mmol). Following silica gel chromatography, product **2u** was obtained as a colorless oil (95.5 mg, 83%). The isolated yield reported in the manuscript is the average of two runs (83% and 79% yield).

^{1}H NMR (400 MHz, CDCl$_3$): 7.72 (dd, *J* = 10.5, 8.7 Hz, 2H), 7.01 (dd, *J* = 6.0, 3.1 Hz, 2H), 3.87 (s, 3H), 1.21 (d, *J* = 16.6 Hz, 9H) ppm.

^{13}C NMR (101 MHz, CDCl$_3$): 163.5, 134.5 (dd, *J* = 11.2, 2.6 Hz), 117.9 (dd, *J* = 126.2, 19.1 Hz), 114.1 (d, *J* = 13.5 Hz), 55.4, 33.1 (dd, *J* = 97.4, 16.0 Hz), 24.0 ppm.

^{31}P NMR (161 MHz, CDCl$_3$): 61.87 (d, $J_{F\text{-}P}$ = 1046.8 Hz) ppm.

^{19}F NMR (376 MHz, CDCl$_3$): -93.76 (d, $J_{P\text{-}F}$ = 1047.1 Hz) ppm.

HRMS ESI (m/z): [M + H] cald for $C_{11}H_{17}FO_2P$: 231.0950; found 231.0940

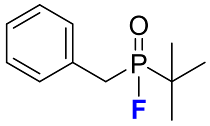

(*tert*-butyl)(benzyl)phosphinic fluoride (2v). The reaction was performed using the standard conditions described above with (*tert*-butyl)(benzyl)phosphinic acid (106.1 mg, 0.5 mmol). Following silica gel chromatography, product **2v** was obtained as a colorless oil (97.5 mg, 91%). The isolated yield reported in the manuscript is the average of two runs (91% and 86% yield).

^{1}H NMR (400 MHz, CDCl$_3$): 7.27 – 7.37 (m, 5H), 3.27 – 3.33 (m, 2H), 1.20 (d, J = 16.4 Hz, 9H) ppm.

^{13}C NMR (101 MHz, CDCl$_3$): 130.0 (d, J = 5.8 Hz), 129.9 (dd, J = 7.9, 1.8 Hz), 129.0 (d, J = 2.5 Hz), 127.5 (d, J = 2.9 Hz), 33.5 (dd, J = 88.4, 12.2 Hz), 31.6 (dd, J = 77.8, 15.4 Hz), 24.3 ppm.

^{31}P NMR (161 MHz, CDCl$_3$): 68.60 (d, $J_{F\text{-}P}$ = 1052.4 Hz) ppm.

^{19}F NMR (376 MHz, CDCl$_3$): -90.59 (d, $J_{P\text{-}F}$ = 1046.7 Hz) ppm.

HRMS ESI (m/z): [M + H] cald for $C_{11}H_{17}FOP$: 215.1001; found 215.0993

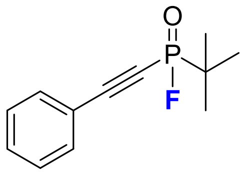

(*tert*-butyl)([2-phenyl]ethenyl)phosphinic fluoride (2w). The reaction was performed using the standard conditions described above with (*tert*-butyl)(2-phenylethynyl)phosphinic acid (111.1 mg, 0.5 mmol). Following silica gel chromatography, product **2w** was obtained as a colorless oil (106.5 mg, 95%). The isolated yield reported in the manuscript is the average of two runs (95% and 93% yield).

^{1}H NMR (400 MHz, CDCl$_3$): 7.59 (d, J = 7.1 Hz, 2H), 7.45 – 7.55 (m, 1H), 7.40 (t, J = 7.5 Hz, 2H), 1.34 (d, J = 19.0 Hz, 9H). ppm.

^{13}C NMR (101 MHz, CDCl$_3$): 132.9 (d, J = 2.0 Hz), 131.4, 128.8, 118.9 (d, J = 3.5 Hz), 103.3 (dd, J = 37.8, 5.9 Hz), 77.0 (dd, J = 170.2, 38.0 Hz), 33.3 (dd, J = 115.4, 14.9 Hz), 23.5. ppm.

^{31}P NMR (161 MHz, CDCl$_3$): 39.36 (d, $J_{F\text{-}P}$ = 1029.1 Hz) ppm.

^{19}F NMR (376 MHz, CDCl$_3$): -79.24 (d, $J_{P\text{-}F}$ = 1029.3 Hz) ppm.

HRMS ESI (m/z): [M + H] cald for $C_{12}H_{15}FOP$: 225.0844; found 225.0836

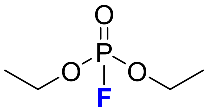

Bis(ethyl)fluorophosphate (2x). The reaction was performed using the standard conditions described above with bis(ethyl)phosphate (77.0 mg, 0.5 mmol). Following the completion of the 60 second reaction time, 4-fluoroanisole (63.0 mg, 0.5 mmol, 1.0 eq.) was added to the reaction. The crude product yield was then determined by ^{19}F NMR spectroscopy by comparison of the internal standard signal to the product signal. The ^{19}F NMR yield reported in the manuscript is the average of two runs (60% and 58%). The ^{19}F and ^{31}P NMR signals were consistent with that published in the literature.[11]

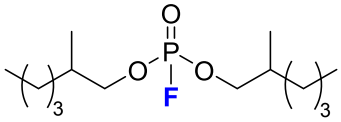

Bis(2-ethylhexyl)fluorophosphate (2y). The reaction was performed using the standard conditions described above with bis(2-ethylhexyl)phosphate (147.2 mg, 0.5 mmol). Following the completion of the 60 second reaction time, 4-fluoroanisole (63.0 mg, 0.5 mmol, 1.0 eq.) was added to the reaction. The crude product yield was then determined by ^{19}F NMR spectroscopy by comparison of the internal standard signal to the product signal. The ^{19}F NMR yield reported in the manuscript is the average of two runs (70% and 69%). The ^{19}F and ^{31}P NMR signals were consistent with that published in the literature.[11]

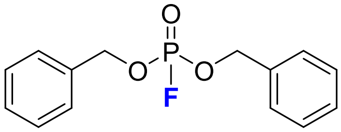

Bis(benzyl)fluorophosphate (2z). The reaction was performed using the standard conditions described above with dibenzylphosphate (139.1 mg, 0.5 mmol). Following the completion of the 60 second reaction time, 4-fluoroanisole (63.0 mg, 0.5 mmol, 1.0 eq.) was added to the reaction. The crude product yield was then determined by ^{19}F NMR spectroscopy by comparison of the internal standard signal to the product signal. The ^{19}F NMR yield reported in the manuscript is the average of two runs (49% and 47%). The ^{19}F and ^{31}P NMR signals were consistent with that published in the literature.[11]

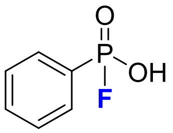

Phenyl phosphonofluoridic acid (2aa). The reaction was performed using the standard conditions described above with phenylphosphonic acid (79.1 mg, 0.5 mmol). Following the completion of the 60 seconds, the reaction was quenched with concentrated HCl. Purification by silica gel chromatography

provided product **2aa** as a white solid (72.0 mg, 90%). The isolated yield reported in the manuscript is the average of two runs (90% and 89% yield).

^{1}H NMR (400 MHz, CDCl$_3$): 10.38 (br, 1H), 7.83 – 7.88 (m, 2H), 7.63 (t, *J* = 7.6 Hz), 7.47 – 7.54 (m, 2H) ppm.

^{13}C NMR (101 MHz, CDCl$_3$): 133.8, 131.7 (d, *J* = 10.6 Hz), 128.8 (d, *J* = 16.2 Hz), 128.1 (dd, *J* = 98.4, 70.1 Hz) ppm.

^{31}P NMR (161 MHz, CDCl$_3$): 15.41 (d, $J_{F\text{-}P}$ = 1007.3 Hz) ppm.

^{19}F NMR (376 MHz, CDCl$_3$): -60.28 (d, $J_{P\text{-}F}$ = 1007.0 Hz) ppm.

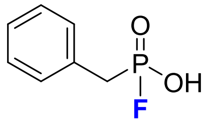

Benzyl phosphonofluoridic acid (2ab). The reaction was performed using the standard conditions described above with phenylphosphonic acid (79.1 mg, 0.5 mmol). Following the completion of the 60 seconds, the reaction was quenched with concentrated HCl. 4-fluoroanisole (63.0 mg, 0.5 mmol, 1.0 eq.) was added to the reaction and the crude product yield was determined by ^{19}F NMR spectroscopy by comparison of the internal standard signal to the product signal. The ^{19}F NMR yield reported in the manuscript is the average of two runs (91% and 88%). The ^{19}F and ^{31}P NMR signals were consistent with that published in the literature.[13]

8. Reaction of phenylphosphinic acid and SIF reagent

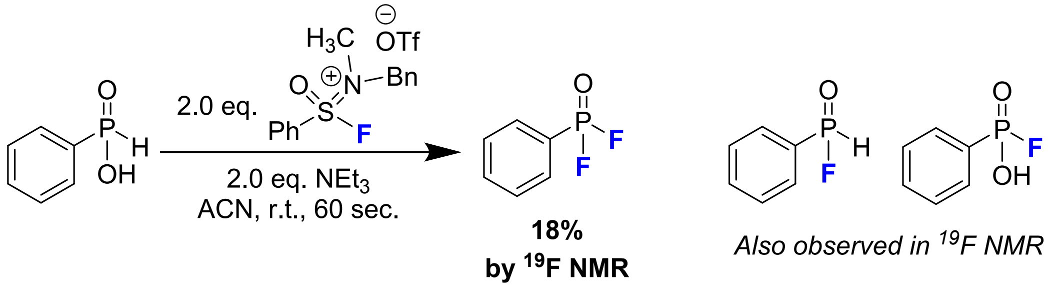

To an 8 mL vial, phenylphosphinic acid (28.2 mg, 0.20 mmol) and NEt_3 (40.4 mg 0.4 mmol) were added along with acetonitrile (0.75 mL). SIF reagent (160 mg, 0.4 mmol) was dissolved in acetonitrile (0.25 mL) and added to the reaction vial at room temperature. The reaction was stirred for 60 seconds at which time 4-fluoroanisole (25.2 mg, 0.2 mmol) was added as an internal standard. The ^{19}F NMR spectrum was recorded and is shown below.

4-fluoranisole displays at -125.3 ppm and is integrated as 1.00 for one equivalent. Three fluorinated products are present in the region between -50 – -70 ppm. Phenyl phosphinic difluoride displays as a doublet at -65.39 ppm and integrates to 18%. The other two products are monofluorinated: phenyl phosphinic fluoride (doublet of doublets at -57.23 ppm) and phenyl phosphonofluoridic acid (doublet at -56.35 ppm).

^{19}F NMR spectra of crude reaction mixture following 60 seconds of reaction time:

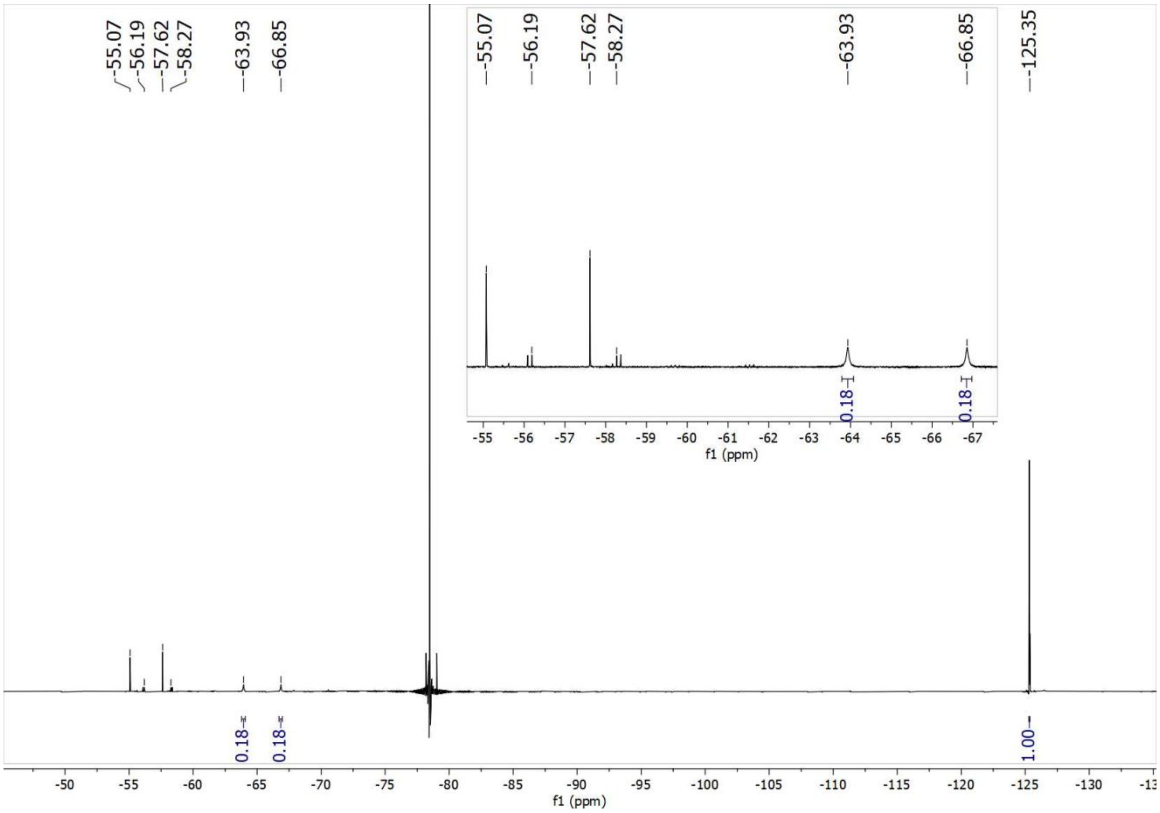

Fluorination of secondary phosphine oxides

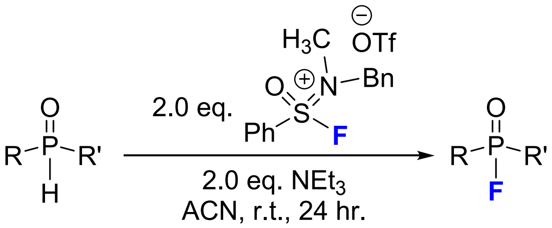

All reactions were performed on the benchtop without the exclusion of air or moisture. Reagents and solvents were used as received and without the need for drying.

To an 8 mL vial, secondary phosphine oxide (0.50 mmol) and NEt_3 (101 mg 1.0 mmol) were added along with acetonitrile (1.5 mL). SIF reagent (401 mg, 1.0 mmol) was dissolved (0.5 mL) and added to the reaction vial at room temperature. The reaction was stirred for 24 hours at which time the solvent was removed under reduced pressure. The crude reaction mixture was then purified via silica gel chromatography using hexane and ethyl acetate as eluent (0 → 50% gradient, 12 gram silica gel column).

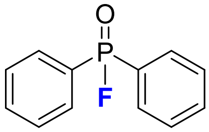

Diphenylphosphinic fluoride (2a). The reaction was performed using the standard conditions described above with diphenylphosphine oxide (101.1 mg, 0.5 mmol). Following silica gel chromatography, product **2a** was obtained as a colorless oil (89.2 mg, 81%). The isolated yield reported in the manuscript is the average of two runs (81% and 79% yield). The ^{1}H, ^{13}C, ^{19}F and ^{31}P NMR spectra were consistent with those published in the literature.[7]

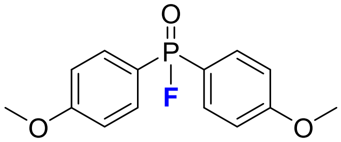

Bis(4-methoxyphenyl)phosphinic fluoride (2b). The reaction was performed using the standard conditions described above with bis(4-methoxyphenyl)phosphine oxide (131.1 mg, 0.5 mmol). Following silica gel chromatography, product **2b** was obtained as a white solid (110.8 mg, 79%). The isolated yield reported in the manuscript is the average of two runs (79% and 78% yield). The ^{1}H, ^{13}C, ^{19}F and ^{31}P NMR spectra were consistent with those published in the literature.[7]

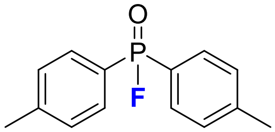

Bis(4-methylphenyl)phosphinic fluoride (2c). The reaction was performed using the standard conditions described above with bis(4-methylphenyl)phosphine oxide (115.1 mg, 0.5 mmol). Following silica gel chromatography, product **2c** was obtained as a white solid (106.7 mg, 86%). The isolated yield reported in

the manuscript is the average of two runs (86% and 84% yield). The ^{1}H, ^{13}C, ^{19}F and ^{31}P NMR spectra were consistent with those published in the literature.[7]

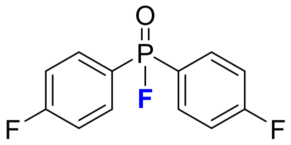

Bis(4-fluorophenyl)phosphinic fluoride (2d). The reaction was performed using the standard conditions described above with bis(4-fluorophenyl)phosphine oxide (119.1 mg, 0.5 mmol). Following silica gel chromatography, product **2d** was obtained as a colorless oil (84.5 mg, 66%). The isolated yield reported in the manuscript is the average of two runs (66% and 60% yield). The ^{1}H, ^{13}C, ^{19}F and ^{31}P NMR spectra were consistent with those published in the literature.[7]

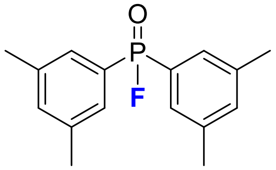

Bis(3,5-dimethylphenyl)phosphinic fluoride (2e). The reaction was performed using the standard conditions described above with bis(3,5-dimethylphenyl)phosphine oxide (129.2 mg, 0.5 mmol). Following silica gel chromatography, product **2e** was obtained as a white solid (107.8 mg, 79%). The isolated yield reported in the manuscript is the average of two runs (79% and 72% yield). The ^{1}H, ^{13}C, ^{19}F and ^{31}P NMR spectra were consistent with those published in the literature.[7]

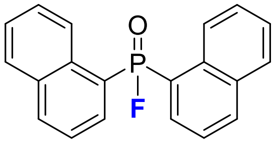

Bis(2-naphthyl)phosphinic fluoride (2g). The reaction was performed using the standard conditions described above with bis(2-naphthyl)phosphine oxide (151.2 mg, 0.5 mmol). Following silica gel chromatography, product **2g** was obtained as a white solid (115.3 mg, 72%). The isolated yield reported in the manuscript is the average of two runs (72% and 71% yield). The ^{1}H, ^{13}C, ^{19}F and ^{31}P NMR spectra were consistent with those published in the literature.[7]

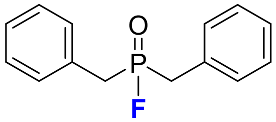

Bis(benzyl)phosphinic fluoride (2j). The reaction was performed using the standard conditions described above with dibenzylphosphine oxide (115.1 mg, 0.5 mmol) and the reaction was performed at 40 °C instead

of room temperature. Following silica gel chromatography, product **2g** was obtained as a white solid (72.0 mg, 58%). The isolated yield reported in the manuscript is the average of two runs (58% and 50% yield). The ^{1}H, ^{13}C, ^{19}F and ^{31}P NMR spectra were consistent with those published in the literature.[7]

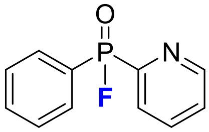

(2-pyridyl)(phenyl)phosphinic fluoride (2n). The reaction was performed using the standard conditions described above with (2-pyridyl)(phenyl)phosphine oxide (101.1 mg, 0.5 mmol). Following silica gel chromatography, product **2n** was obtained as a pale yellow oil (88.5 mg, 80%). The isolated yield reported in the manuscript is the average of two runs (80% and 78% yield). The spectroscopic data matched that reported above in Section 7.

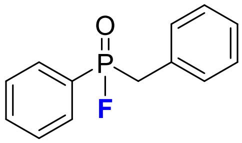

(benzyl)(phenyl)phosphinic fluoride (2q). The reaction was performed using the standard conditions described above with (benzyl)(phenyl)phosphine oxide (108.1 mg, 0.5 mmol). Following silica gel chromatography, product **2q** was obtained as a white solid (94.8 mg, 81%). The isolated yield reported in the manuscript is the average of two runs (81% and 76% yield). The spectroscopic data matched that reported above in Section 7.

9. ^{1}H, ^{13}C, ^{19}F and ^{31}P NMR spectra of fluorination products

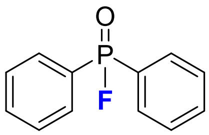

Diphenylphosphinic fluoride (2a).

^{1}H NMR: 400 MHz in $CDCl_3$

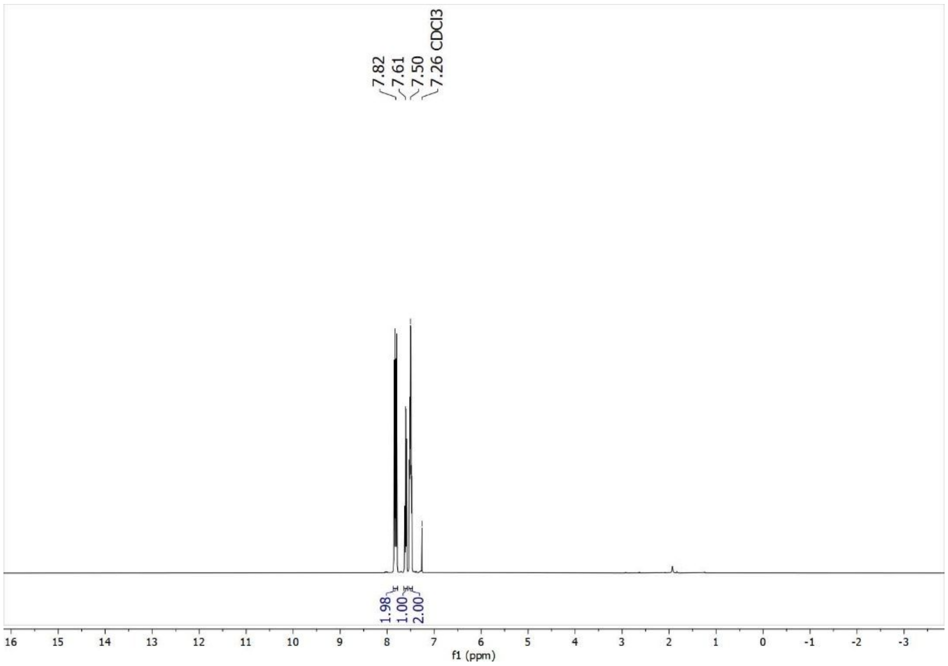

^{13}C NMR: 101 MHz in $CDCl_3$

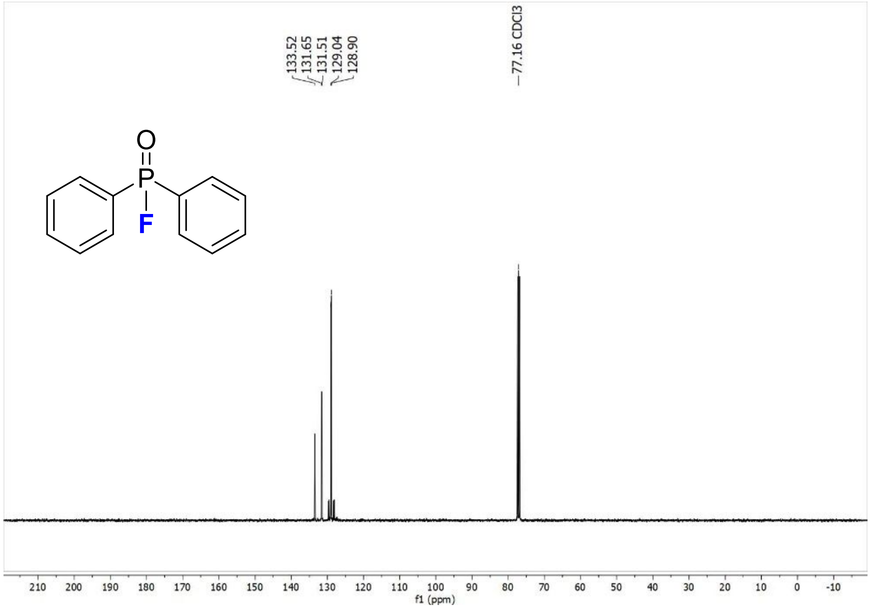

^{19}F NMR: 376 MHz in $CDCl_3$

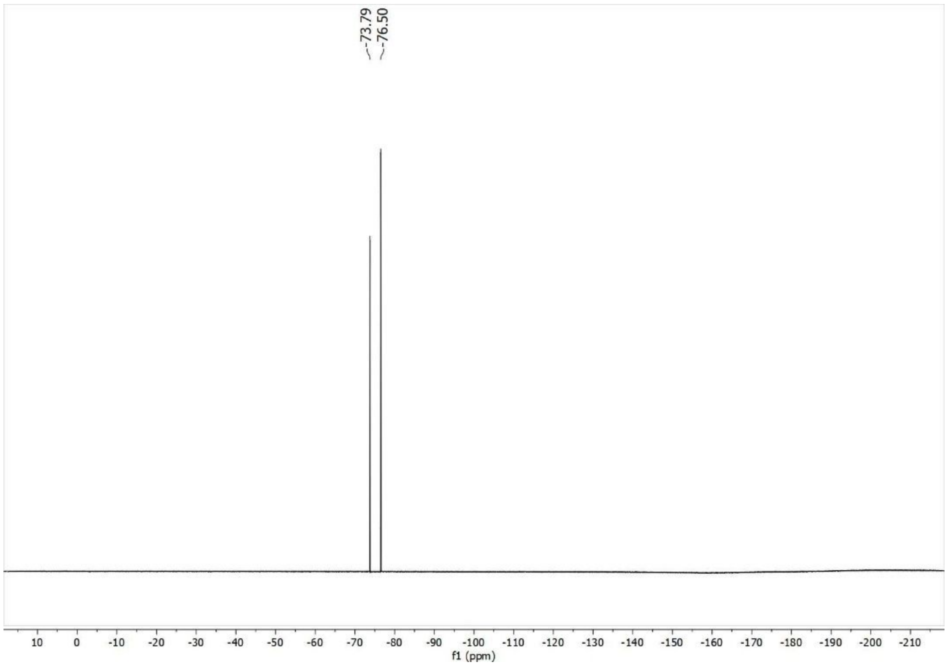

^{31}P NMR: 161 MHz in $CDCl_3$

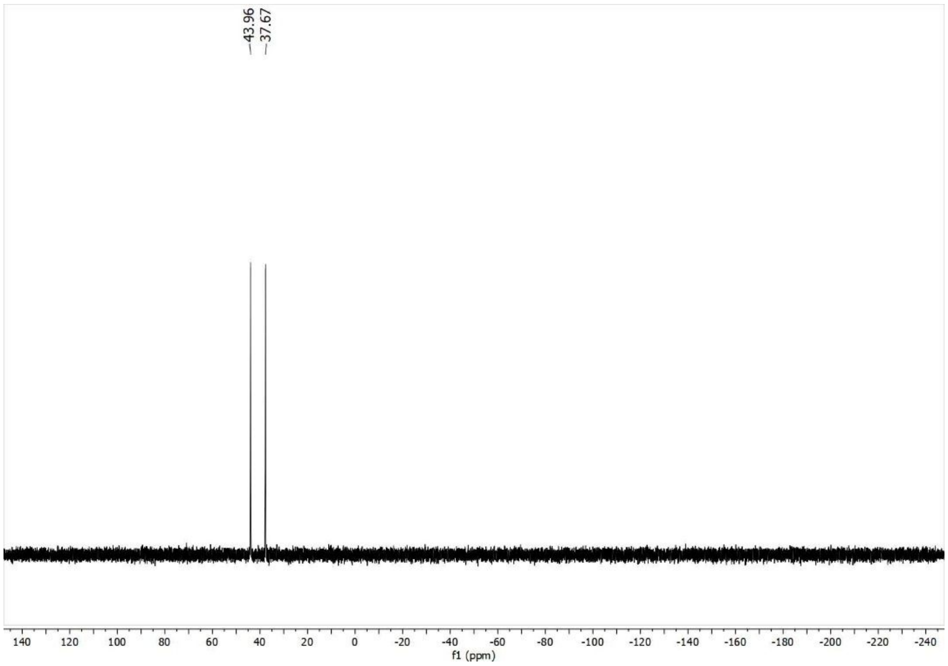

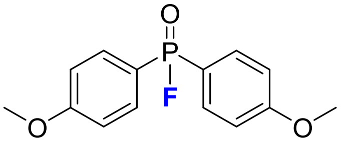

Bis(4-methoxyphenyl)phosphinic fluoride (2b).

^{1}H NMR: 400 MHz in $CDCl_3$

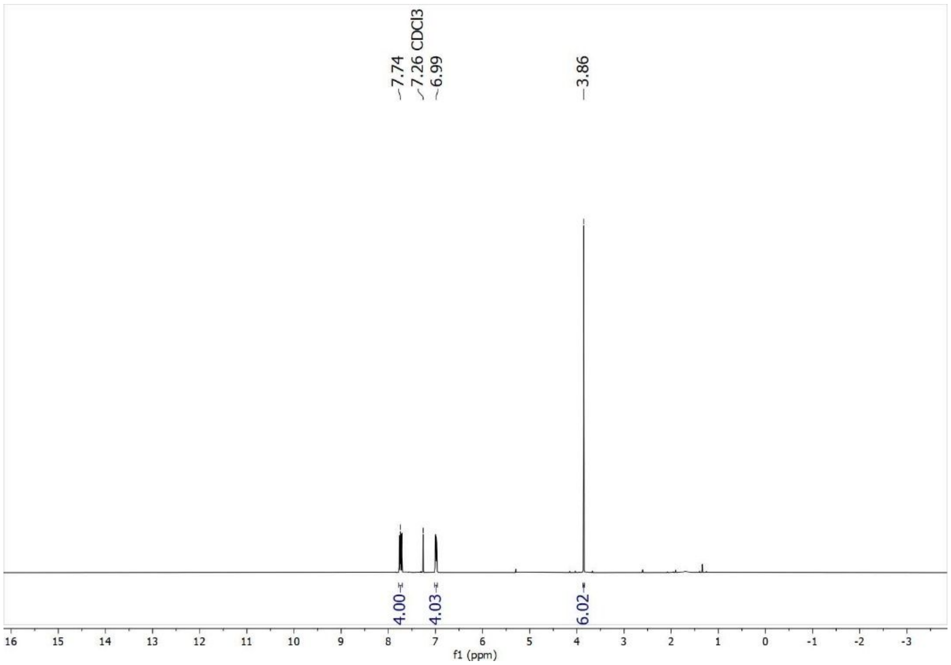

^{13}C NMR: 101 MHz in $CDCl_3$

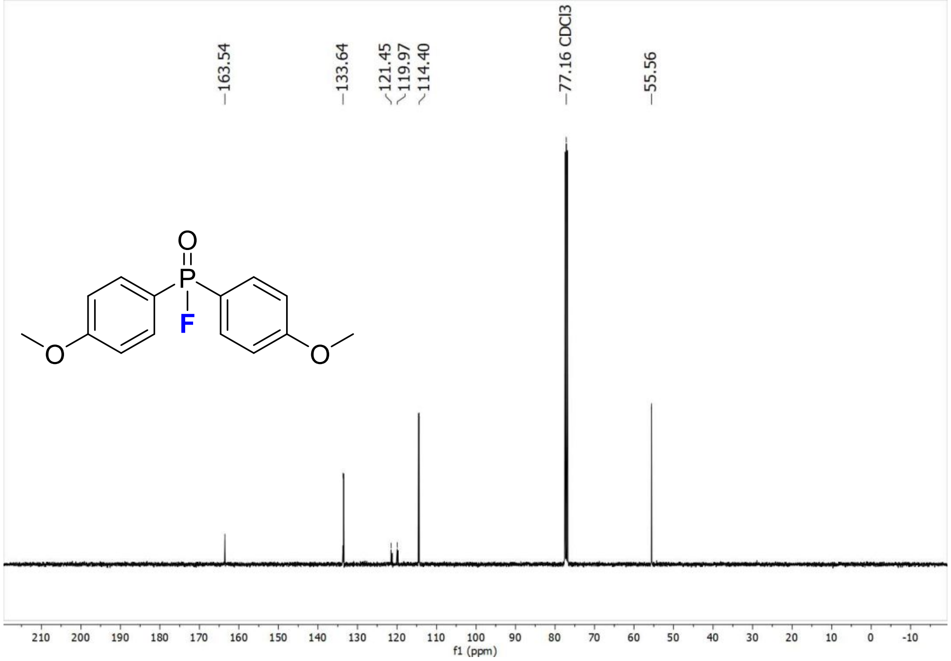

^{19}F NMR: 376 MHz in $CDCl_3$

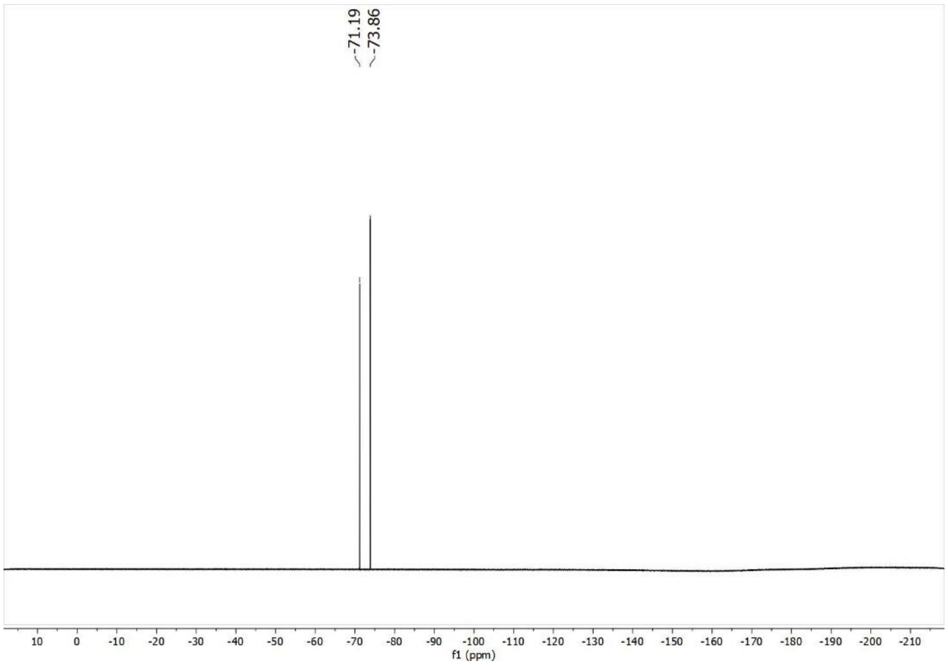

^{31}P NMR: 161 MHz in $CDCl_3$

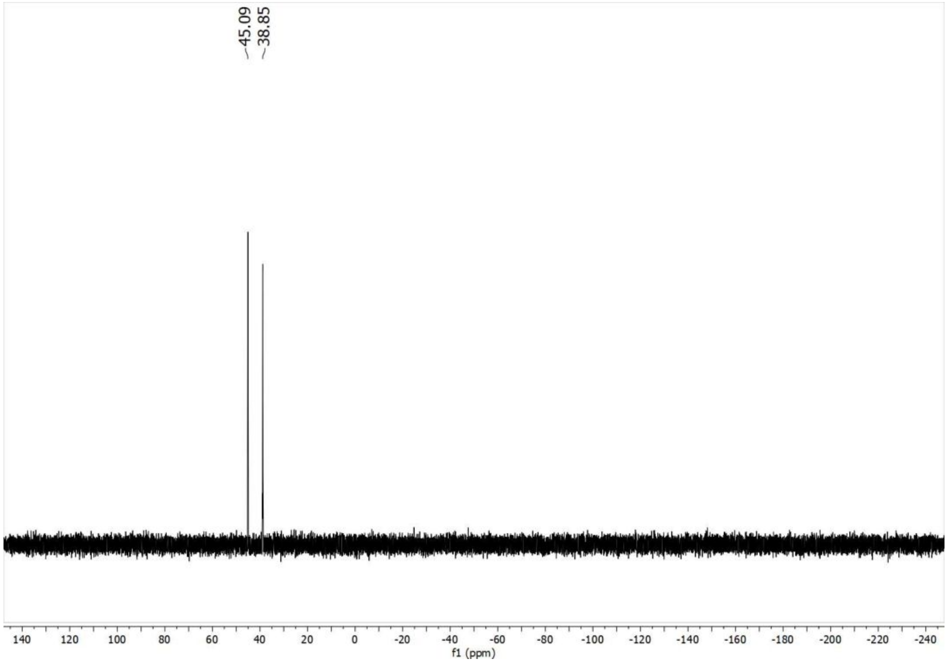

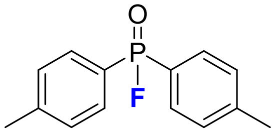

Bis(*para*-tolyl)phosphinic fluoride (2c).

^{1}H NMR: 400 MHz in $CDCl_3$

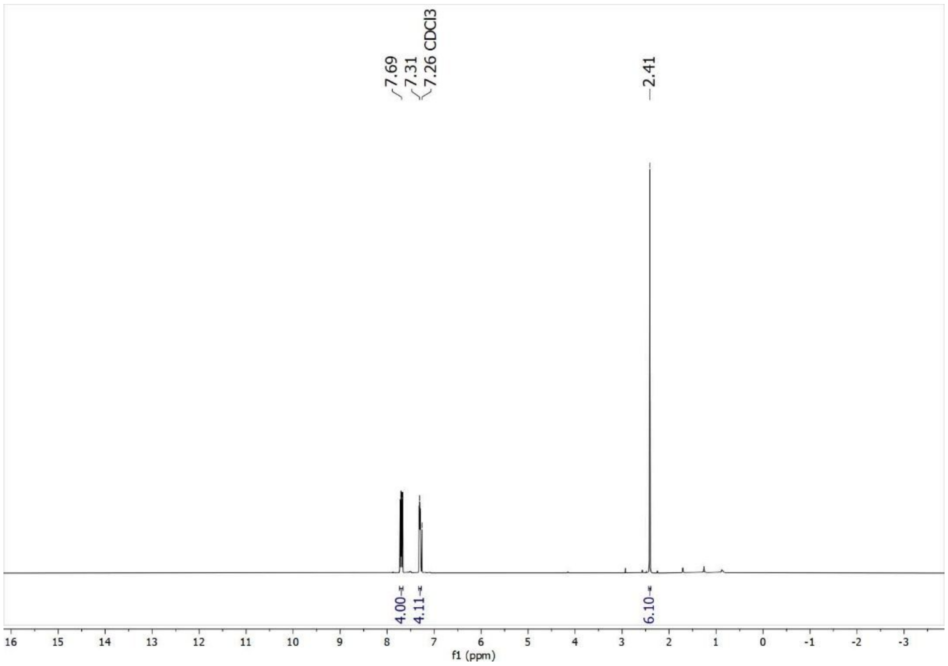

^{13}C NMR: 101 MHz in $CDCl_3$

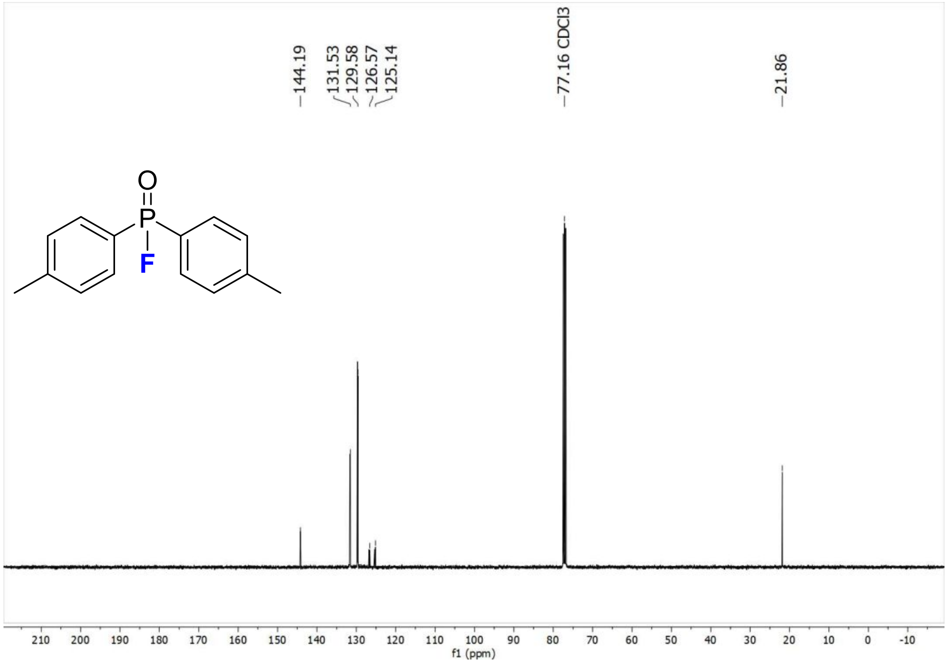

^{19}F NMR: 376 MHz in $CDCl_3$

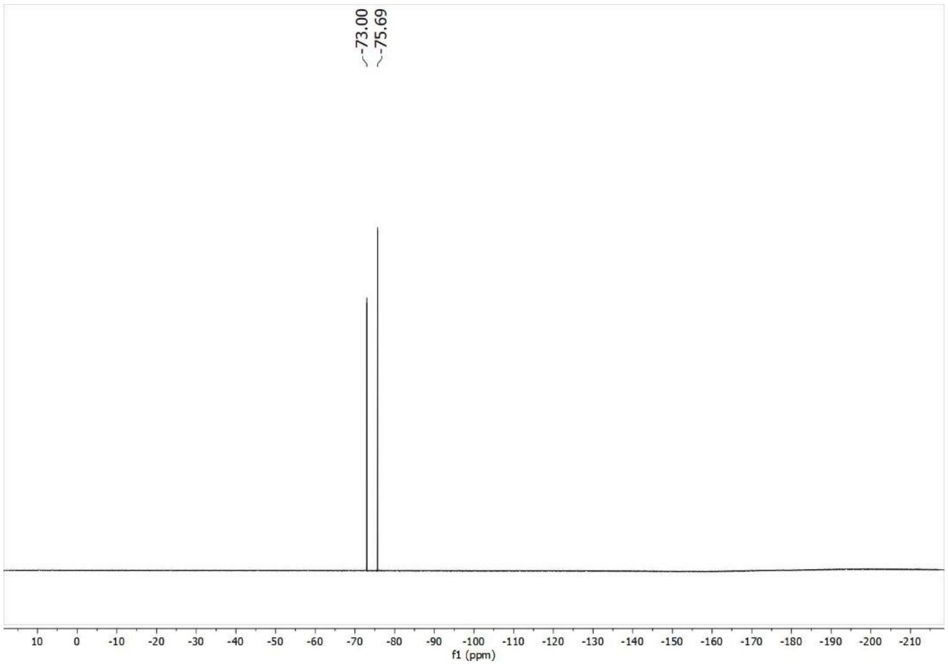

^{31}P NMR: 161 MHz in $CDCl_3$

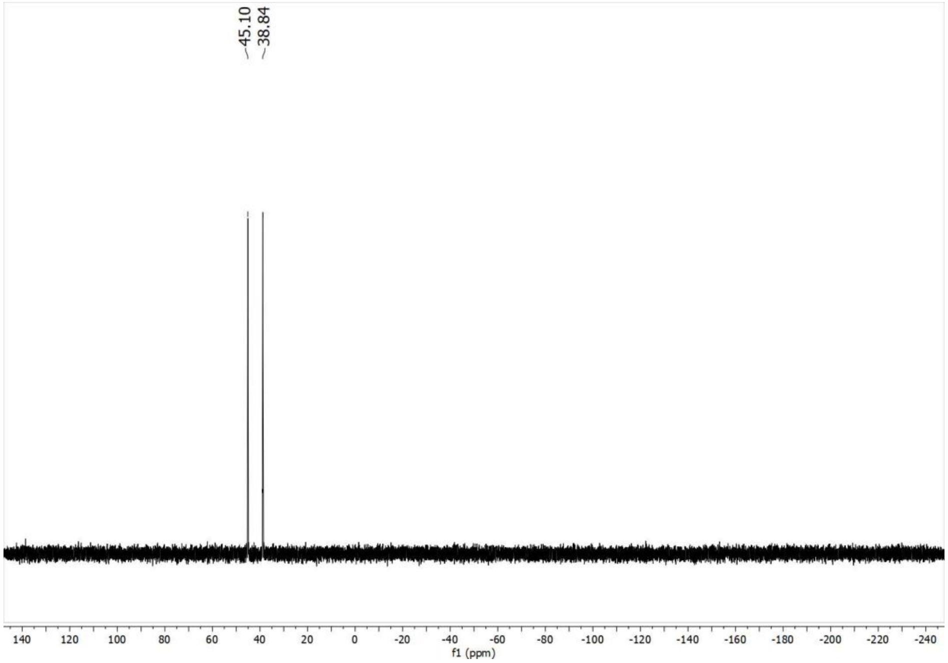

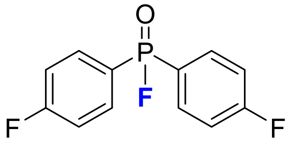

Bis(4-fluorophenyl)phosphinic fluoride (2d).

^{1}H NMR: 400 MHz in $CDCl_3$

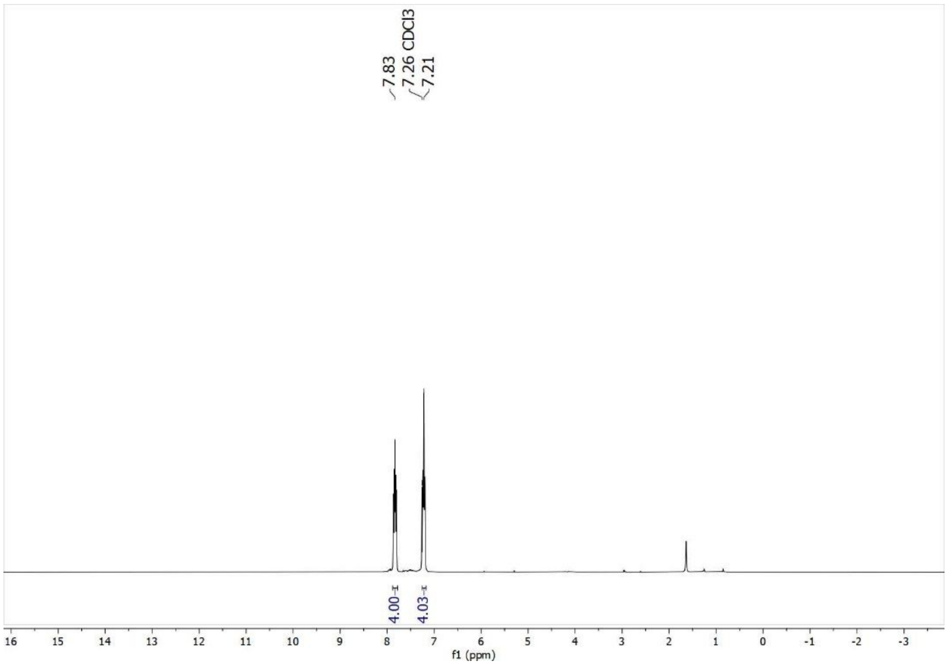

^{13}C NMR: 101 MHz in $CDCl_3$

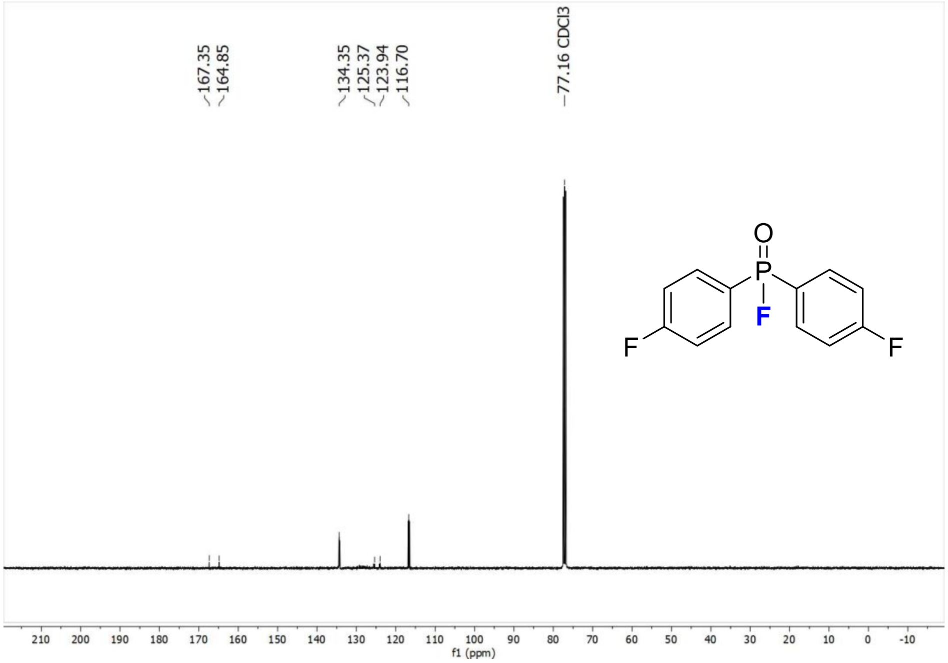

^{19}F NMR: 376 MHz in $CDCl_3$

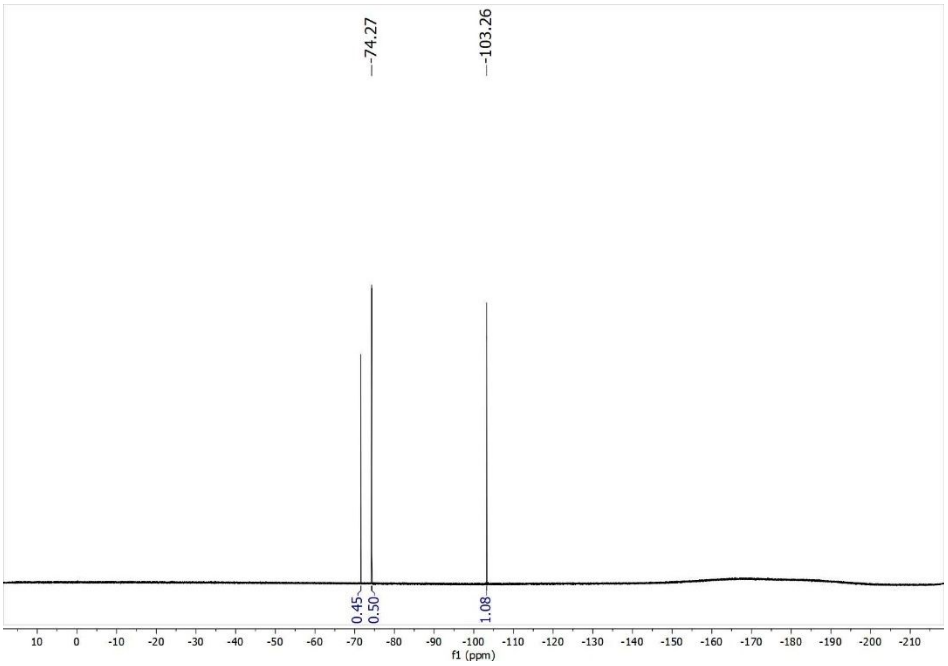

^{31}P NMR: 161 MHz in $CDCl_3$

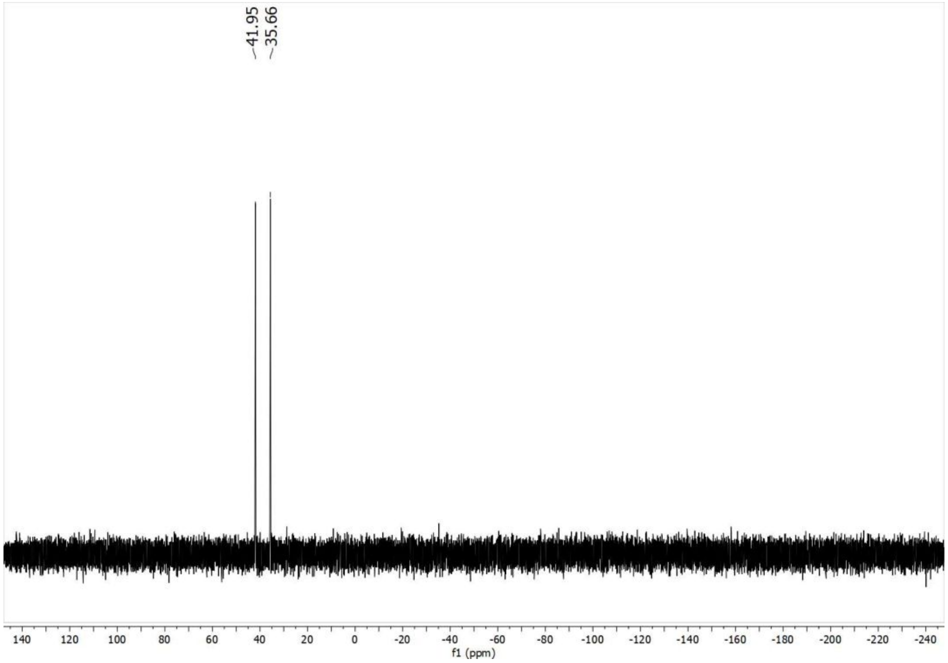

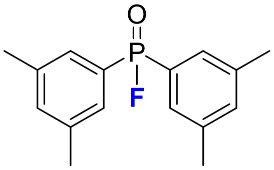

Bis(3,5-dimethylphenyl)phosphinic fluoride (2e).

^{1}H NMR: 400 MHz in $CDCl_3$

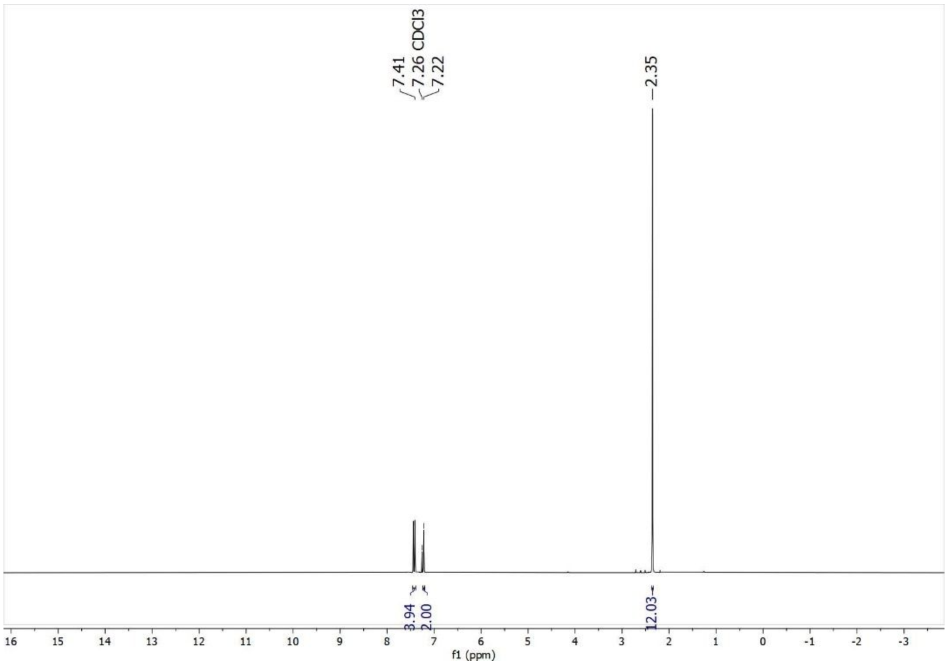

^{13}C NMR: 101 MHz in $CDCl_3$

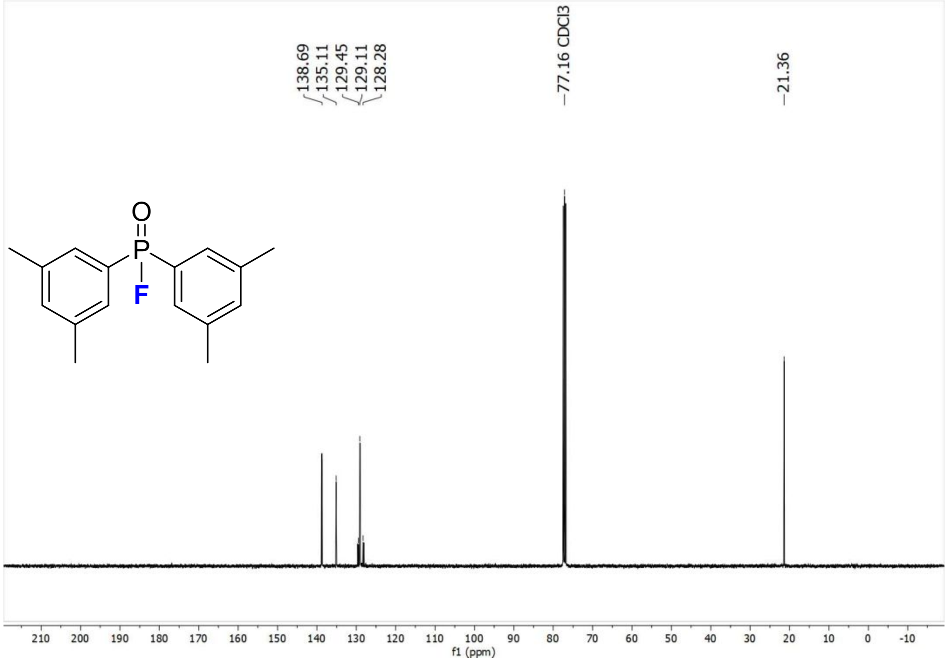

^{19}F NMR: 376 MHz in $CDCl_3$

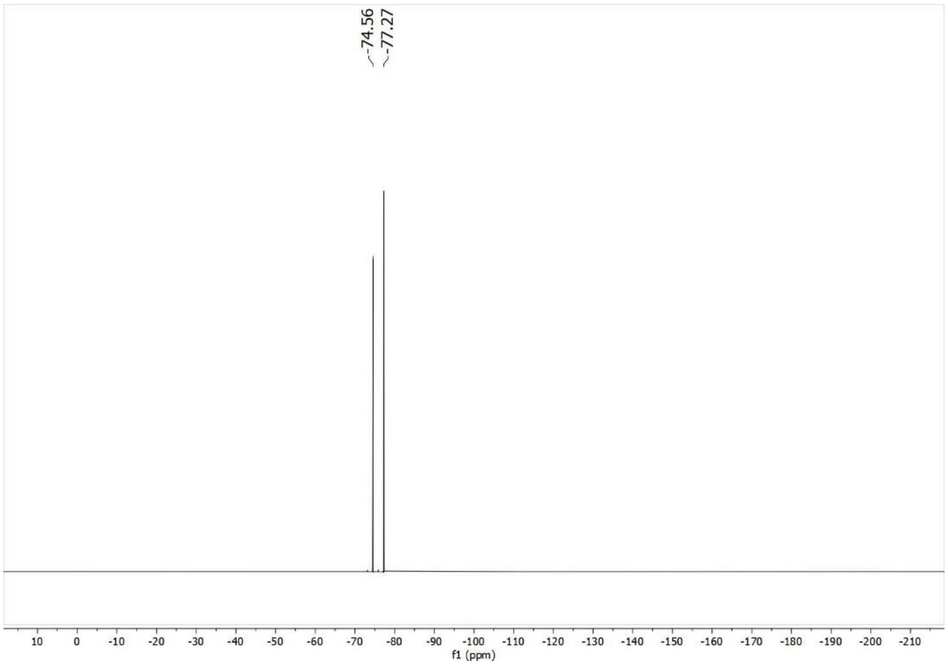

^{31}P NMR: 161 MHz in $CDCl_3$

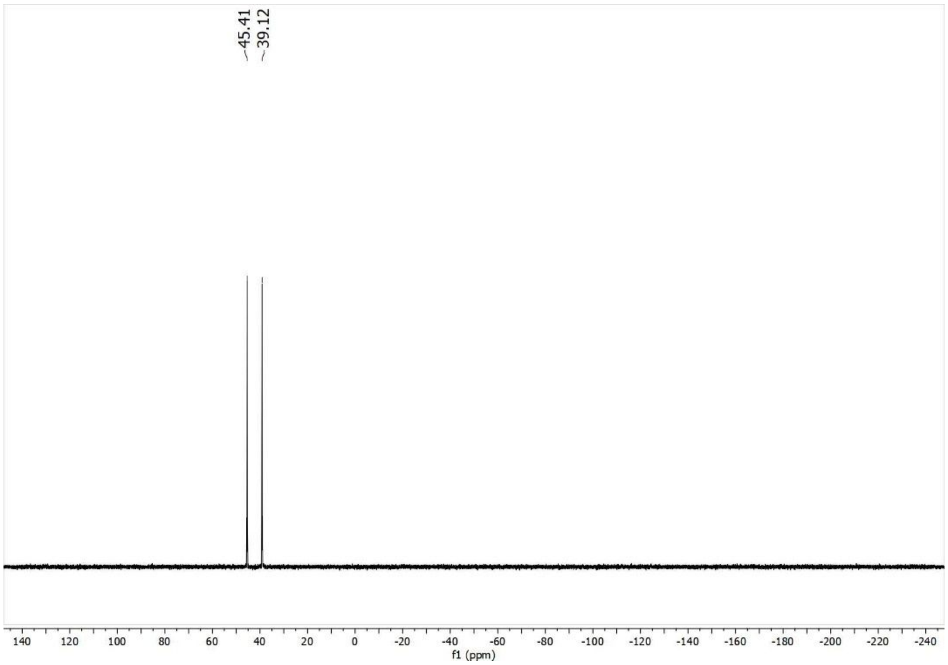

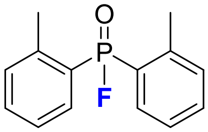

Bis(2-methylphenyl)phosphinic fluoride (2f).

^{1}H NMR: 400 MHz in $CDCl_3$

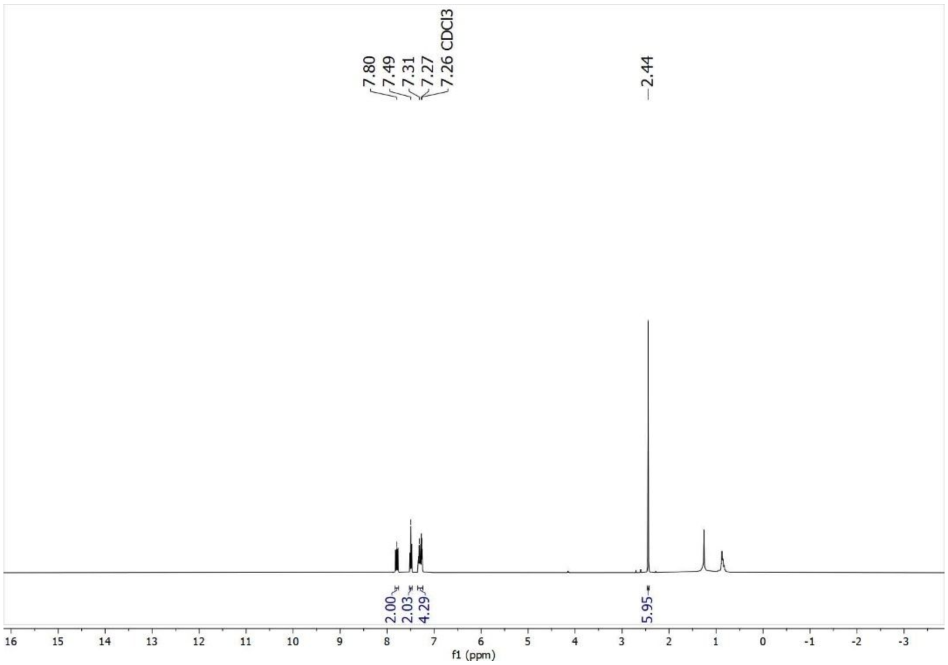

^{13}C NMR: 101 MHz in $CDCl_3$

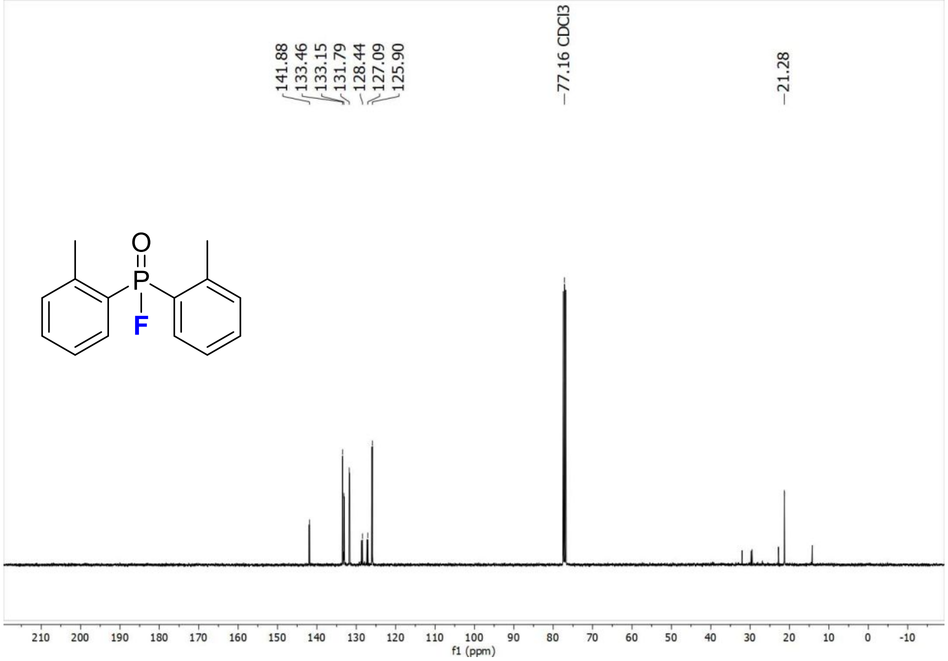

^{19}F NMR: 376 MHz in $CDCl_3$

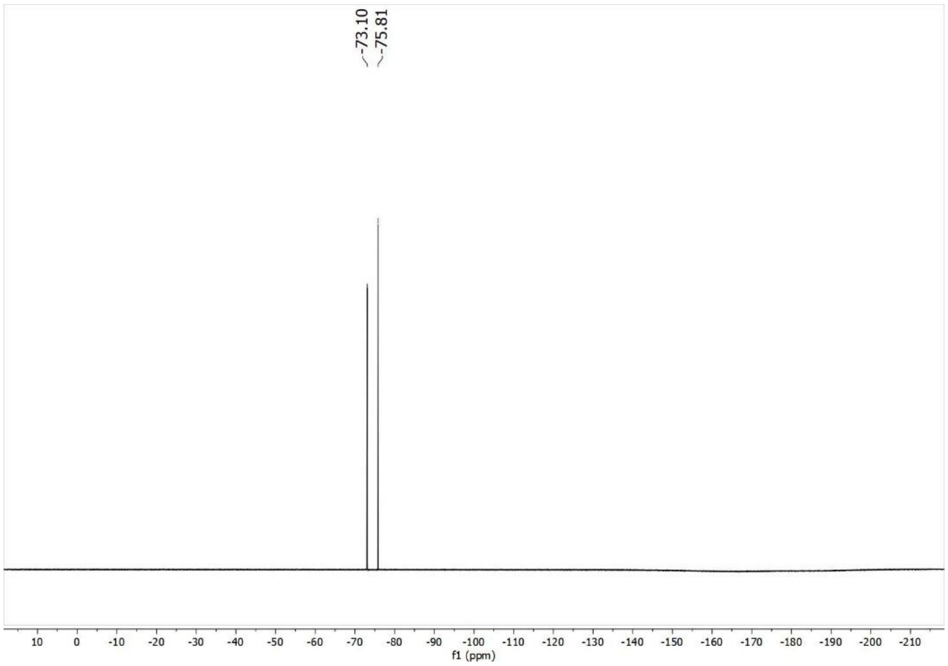

^{31}P NMR: 161 MHz in $CDCl_3$

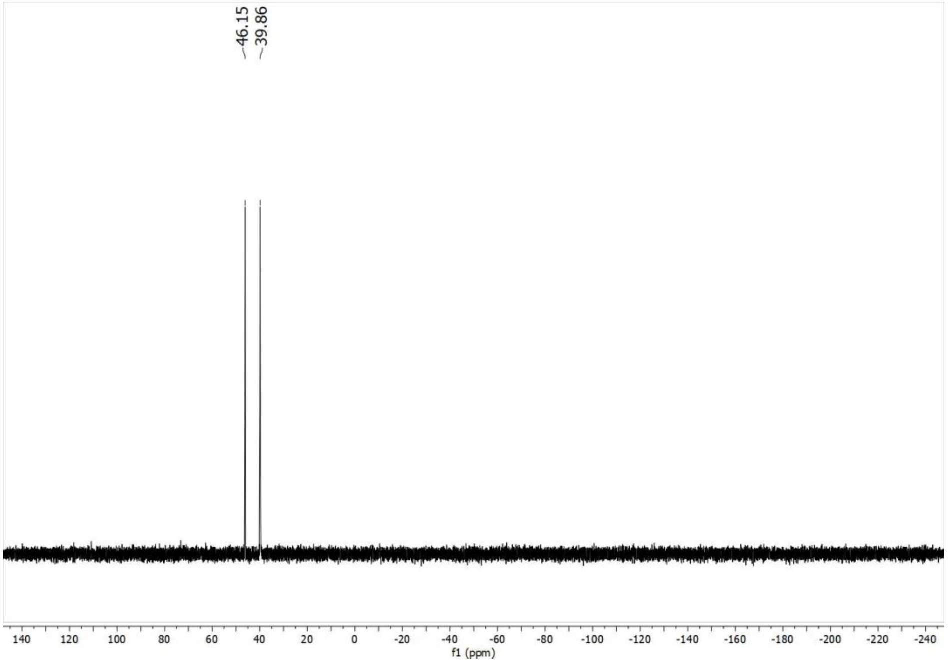

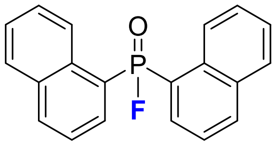

Bis(2-naphthyl)phosphinic fluoride (2g).

^{1}H NMR: 400 MHz in $CDCl_3$

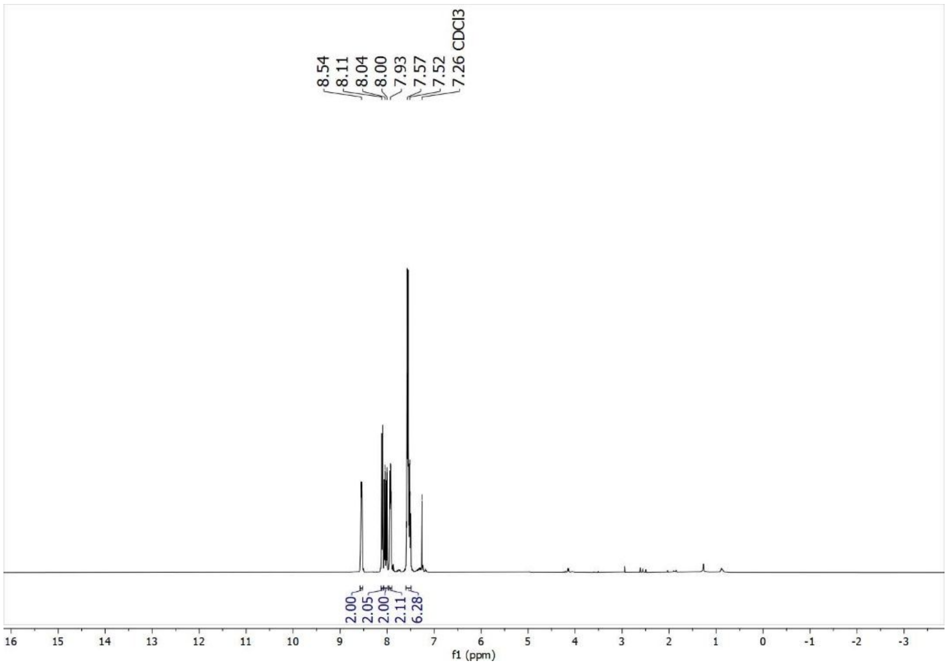

^{13}C NMR: 101 MHz in $CDCl_3$

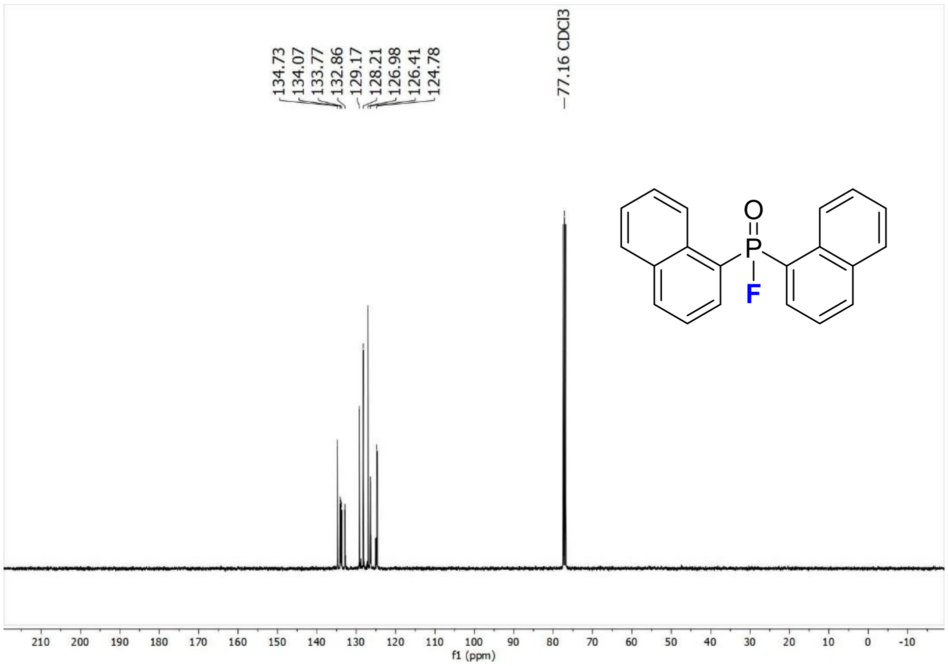

^{19}F NMR: 376 MHz in $CDCl_3$

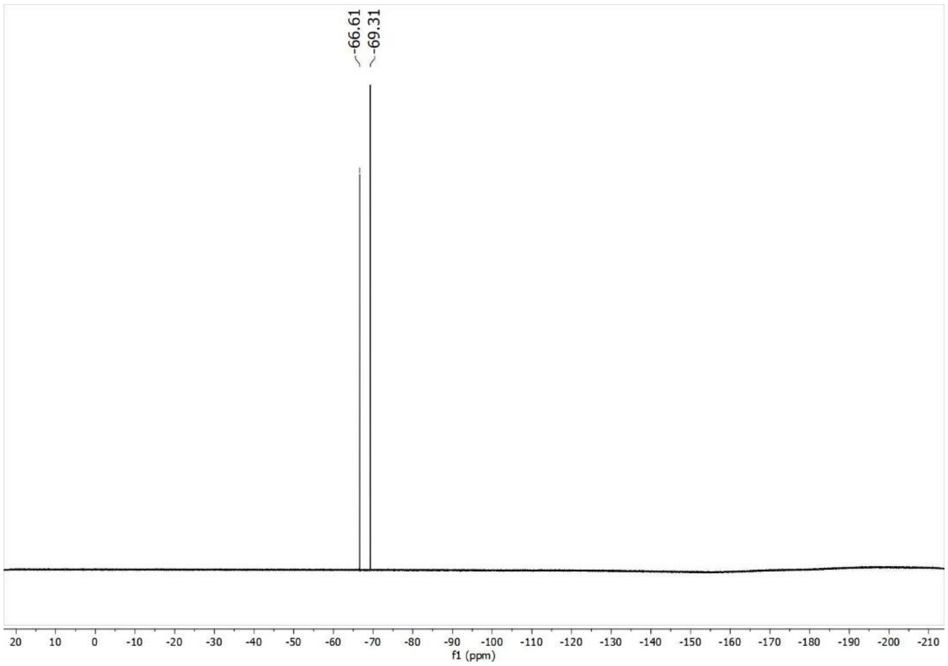

^{31}P NMR: 161 MHz in $CDCl_3$

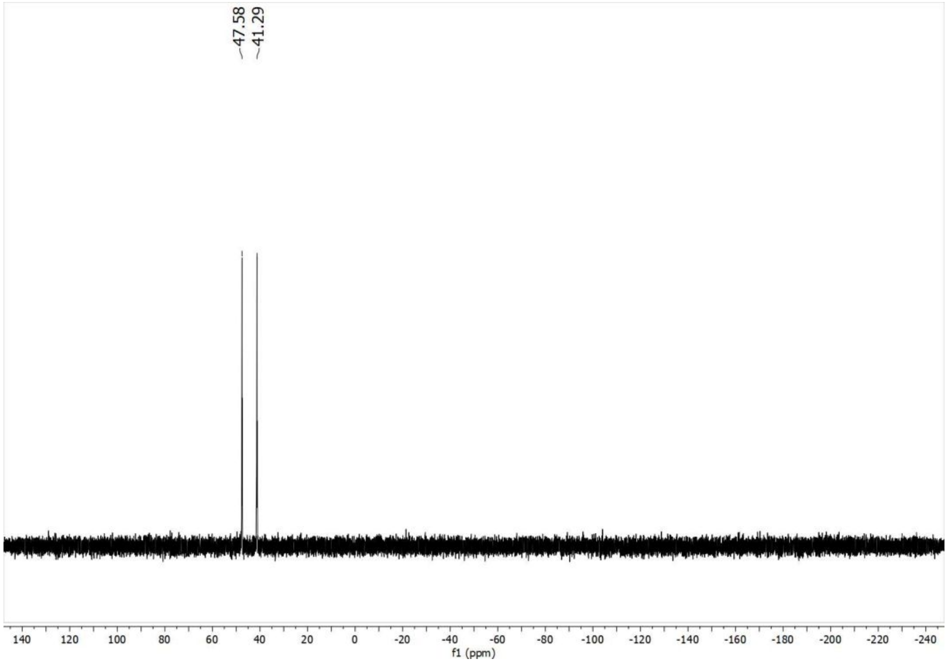

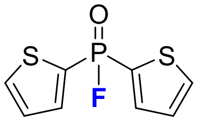

Bis(2-thienyl)phosphinic fluoride (2h).

Crude ^{19}F spectrum with 1.0 equivalents of 4-fluoroanisole as internal standard (93% yield): 376 MHz in CH_3CN

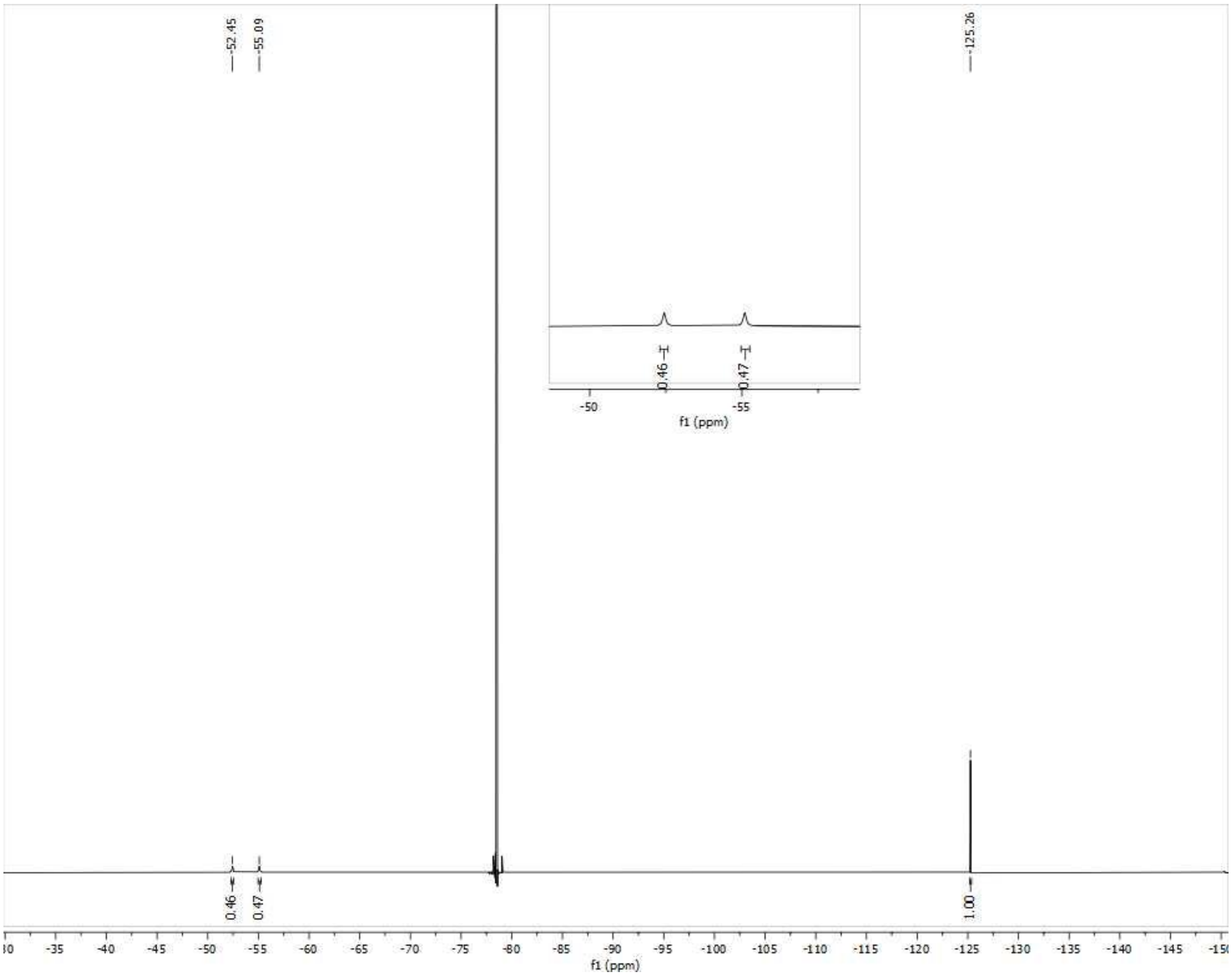

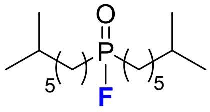

Bis(isooctyl)phosphinic fluoride (2i).

Crude ^{19}F spectrum with 1.0 equivalents of 4-fluoroanisole as internal standard (94% yield): 376 MHz in CH_3CN

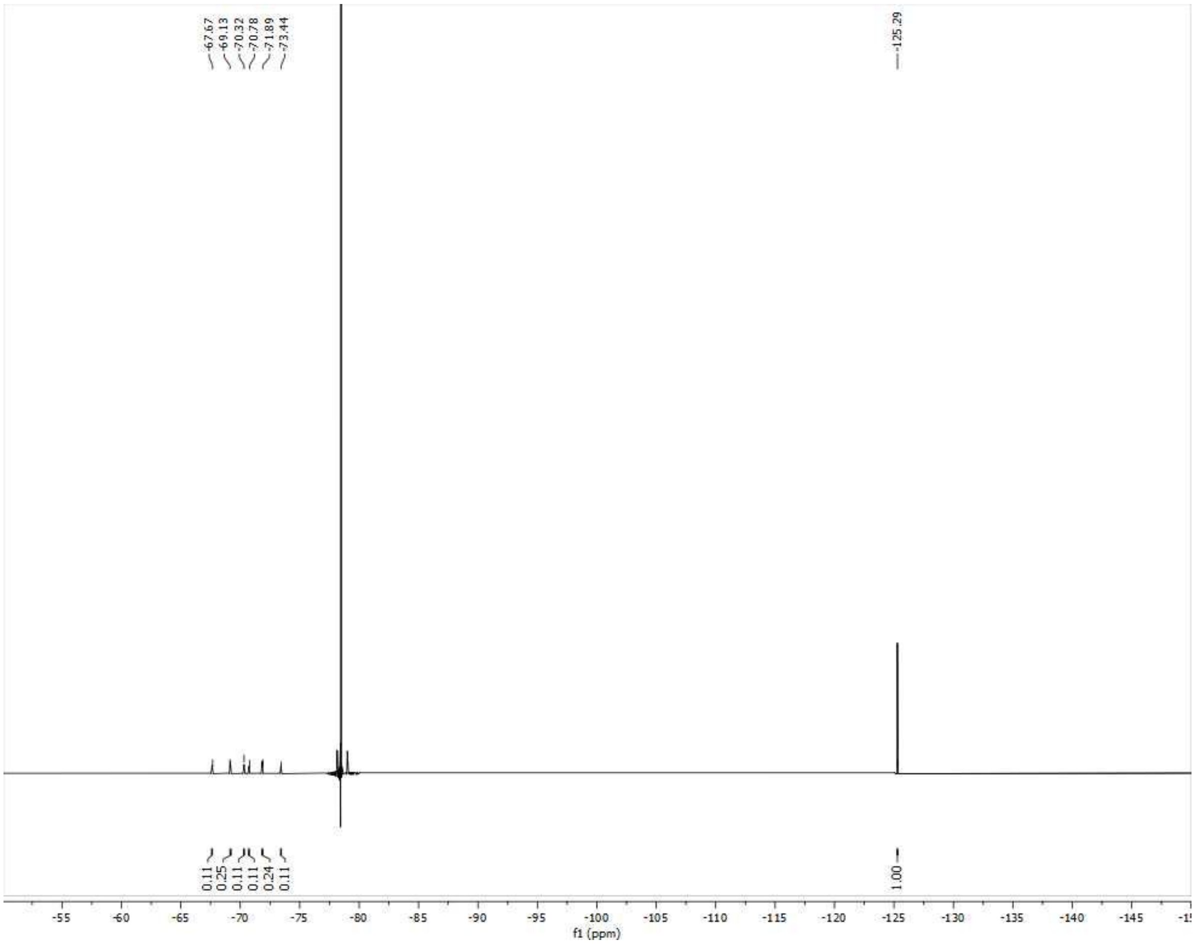

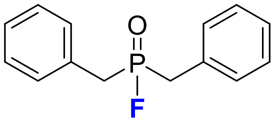

Bis(benzyl)phosphinic fluoride (2j).

^{1}H NMR: 400 MHz in $CDCl_3$

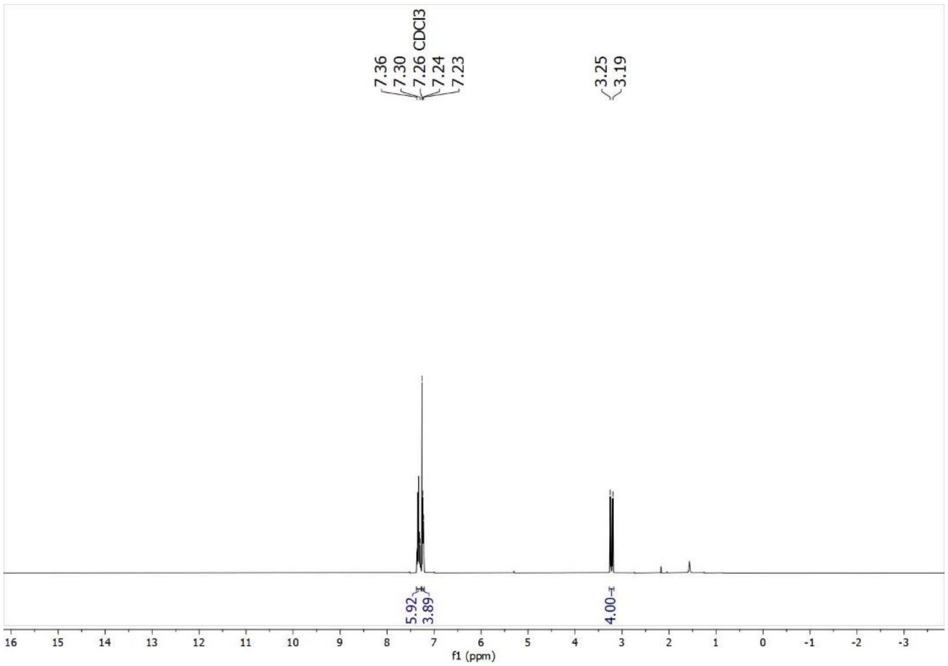

^{13}C NMR: 101 MHz in $CDCl_3$

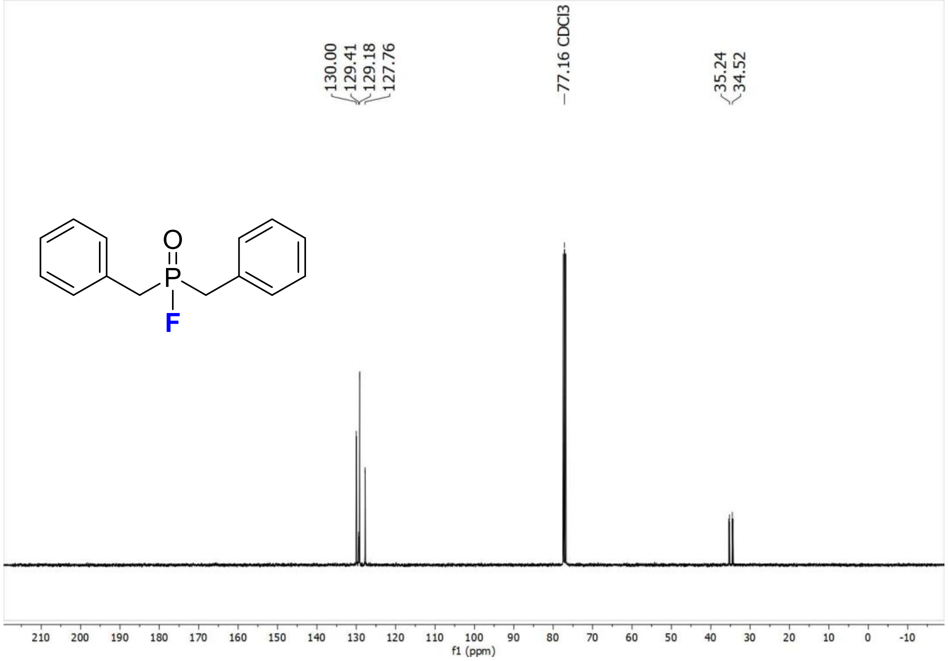

^{19}F NMR: 376 MHz in $CDCl_3$

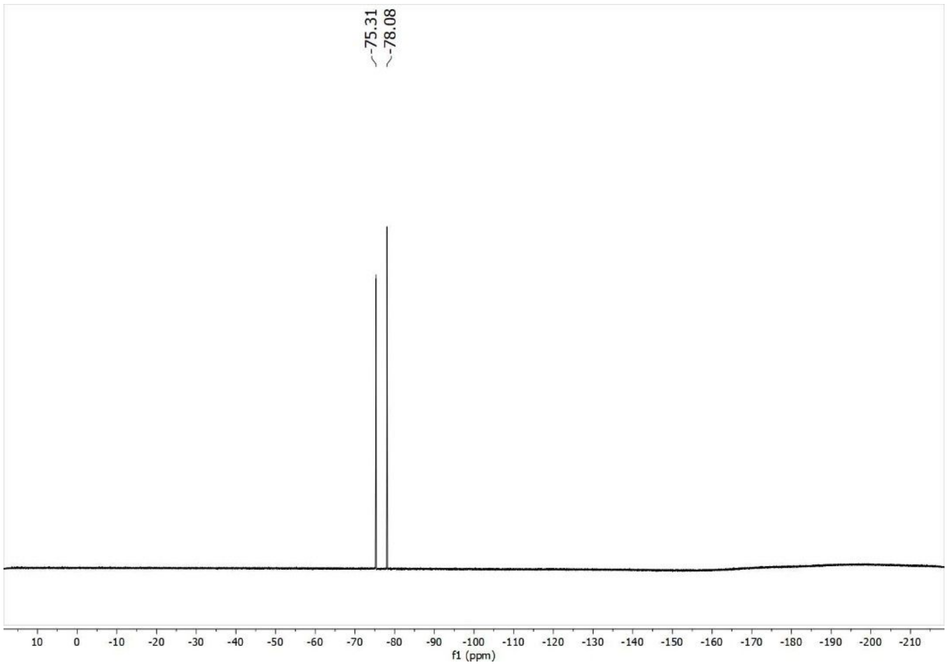

^{31}P NMR: 161 MHz in $CDCl_3$

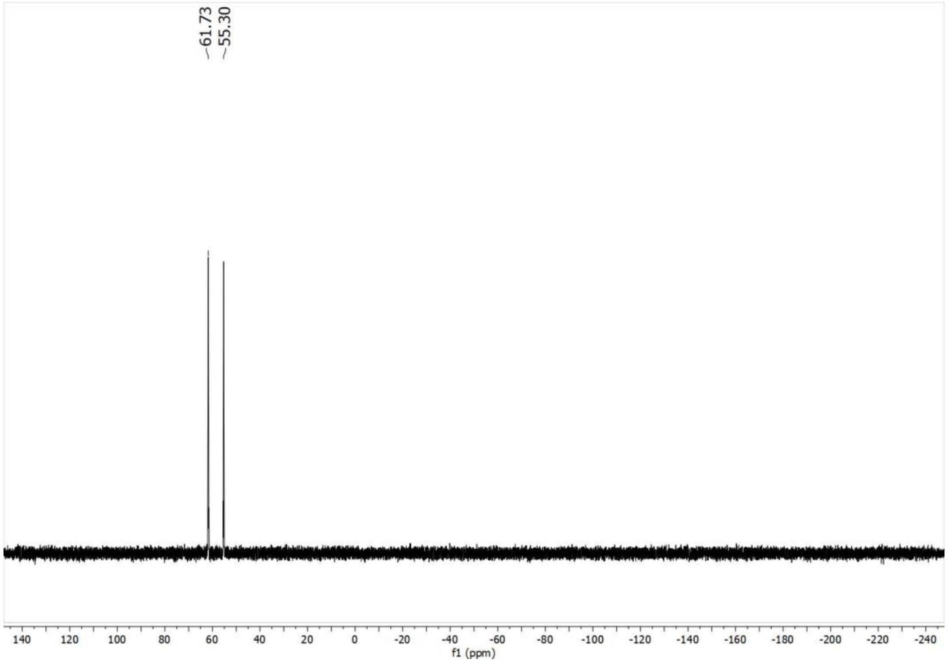

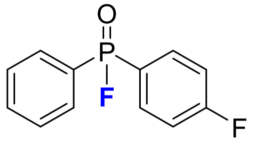

(4-fluorophenyl)(phenyl)phosphinic fluoride (2k).

^{1}H NMR: 400 MHz in $CDCl_3$

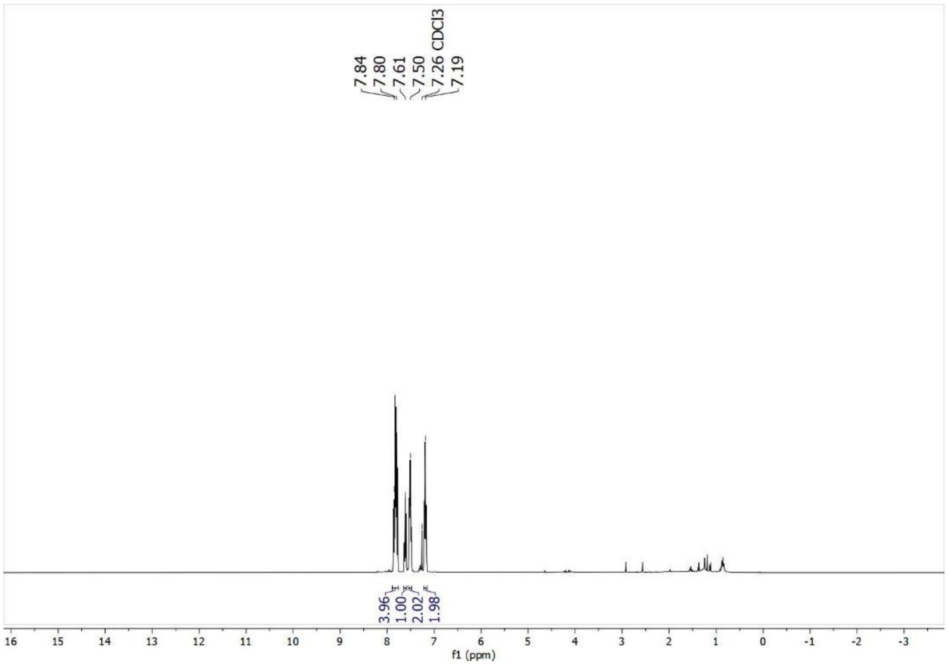

^{13}C NMR: 101 MHz in $CDCl_3$

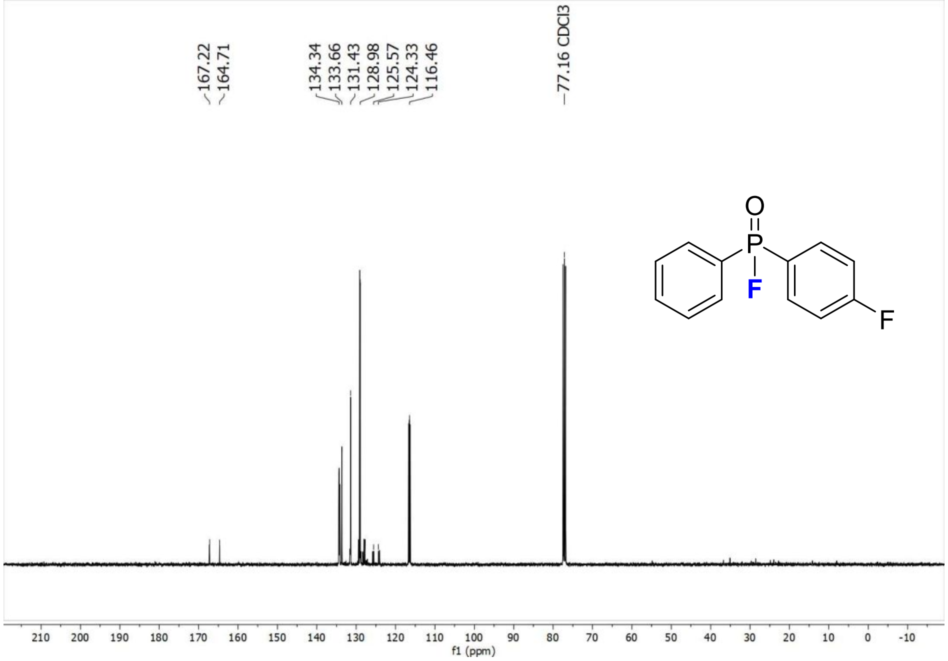

^{19}F NMR: 376 MHz in $CDCl_3$

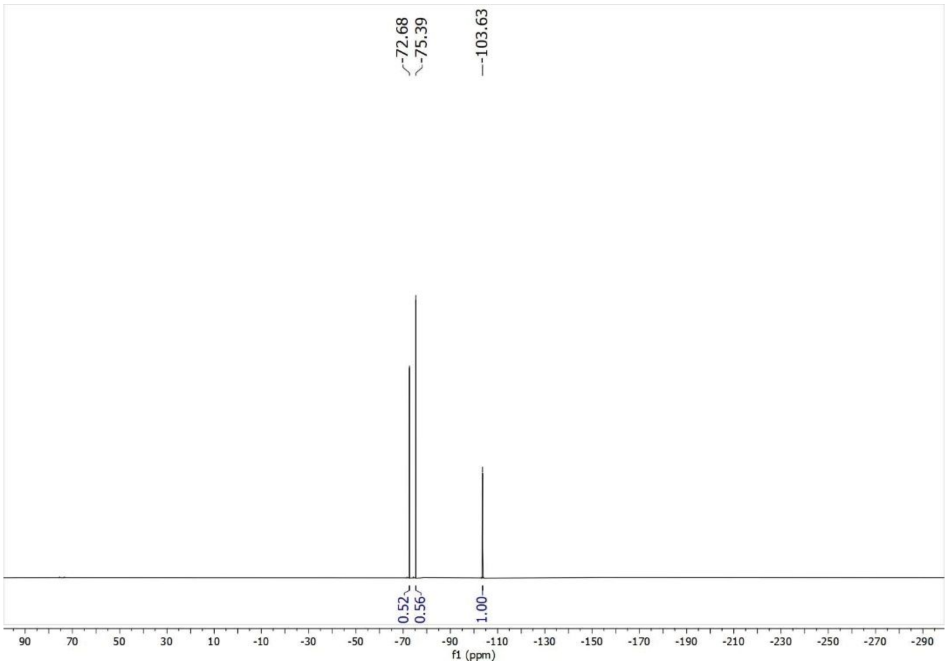

^{31}P NMR: 161 MHz in $CDCl_3$

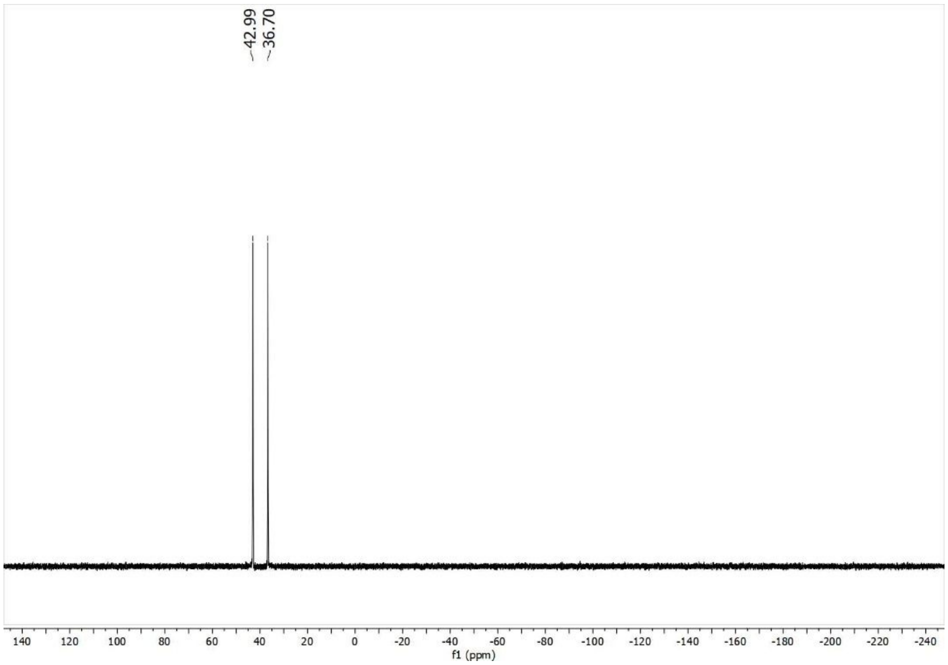

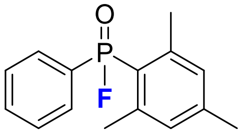

(2,4,6-trimethylphenyl)(phenyl)phosphinic fluoride (2l).

^{1}H NMR: 400 MHz in $CDCl_3$

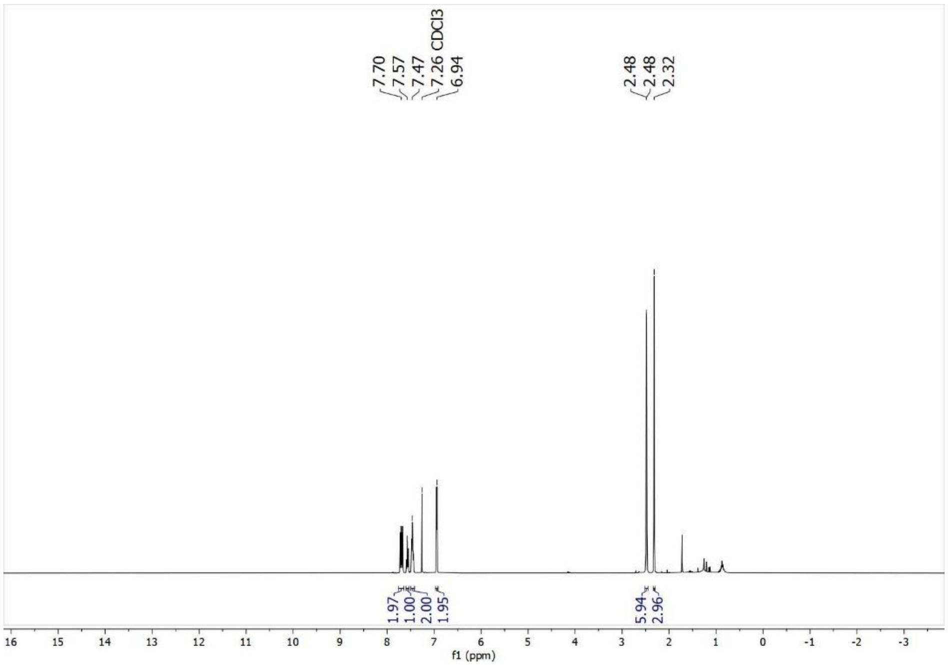

^{13}C NMR: 101 MHz in $CDCl_3$

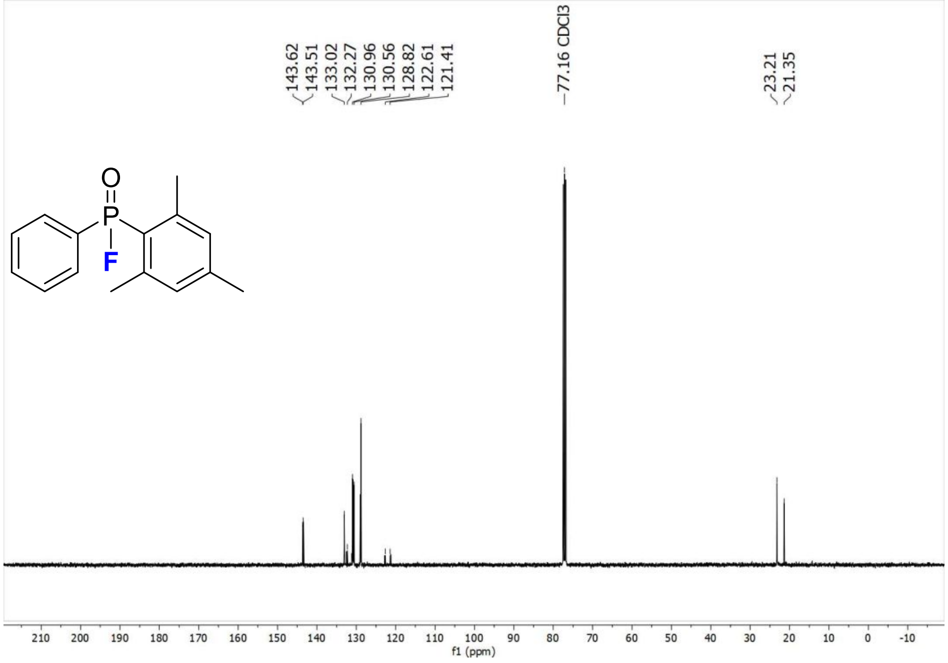

^{19}F NMR: 376 MHz in $CDCl_3$

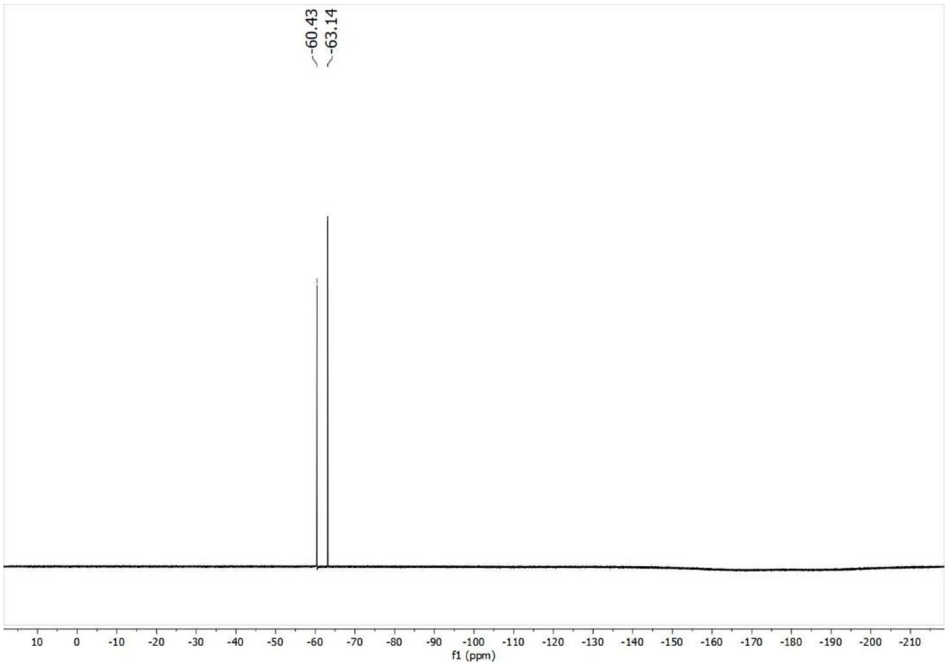

^{31}P NMR: 161 MHz in $CDCl_3$

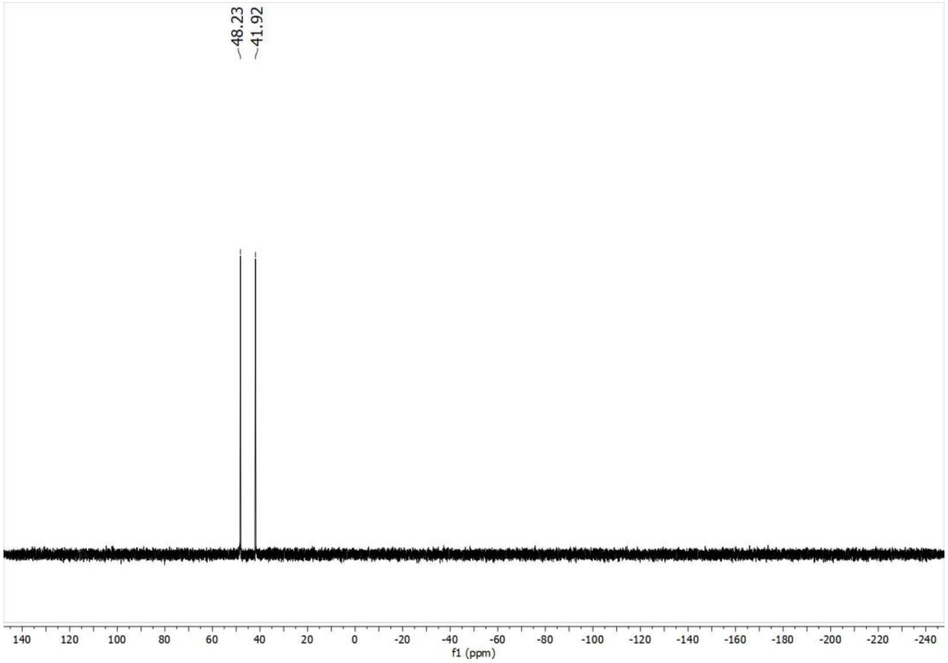

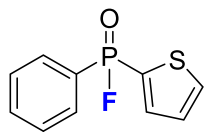

(2-thienyl)(phenyl)phosphinic fluoride (2m).

Crude ^{19}F spectrum with 1.0 equivalents of 4-fluoroanisole as internal standard (89% yield): 376 MHz in CH_3CN

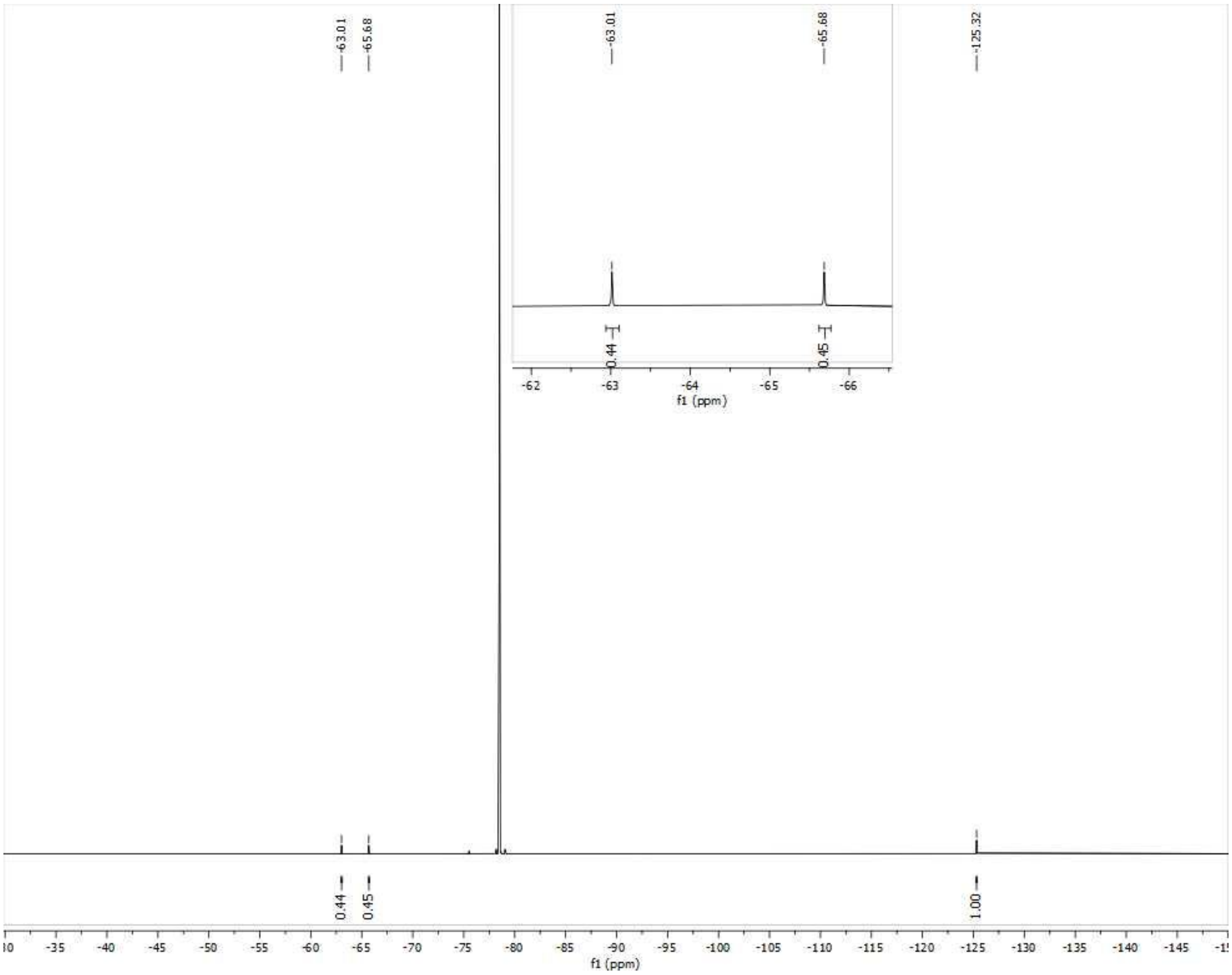

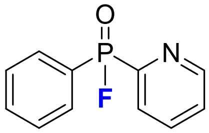

(2-pyridyl)(phenyl)phosphinic fluoride (2n).

^{1}H NMR: 400 MHz in $CDCl_3$ (minimal amount of hexanes solvent could not be removed)

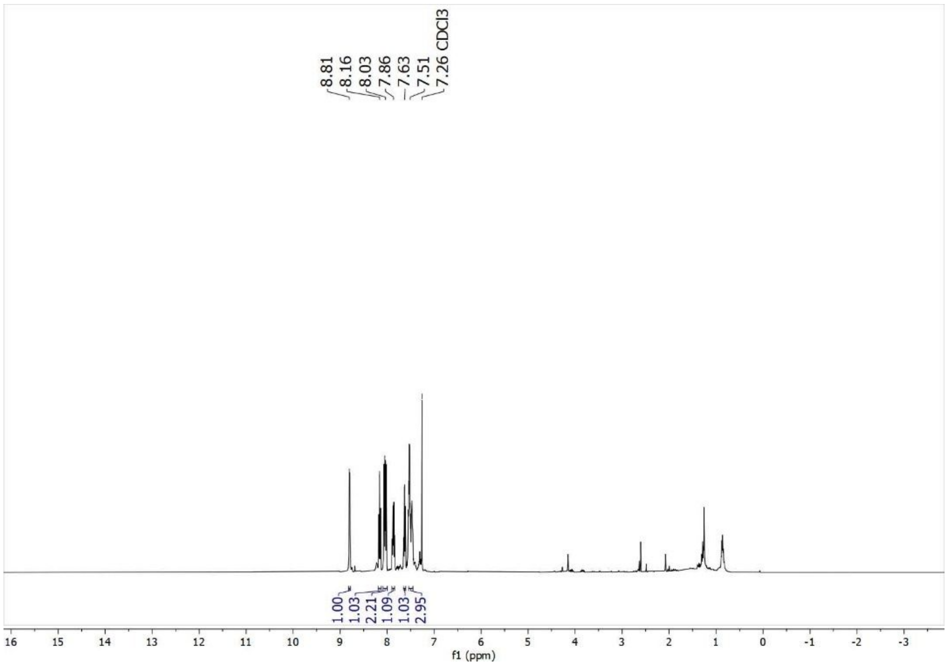

^{13}C NMR: 101 MHz in $CDCl_3$

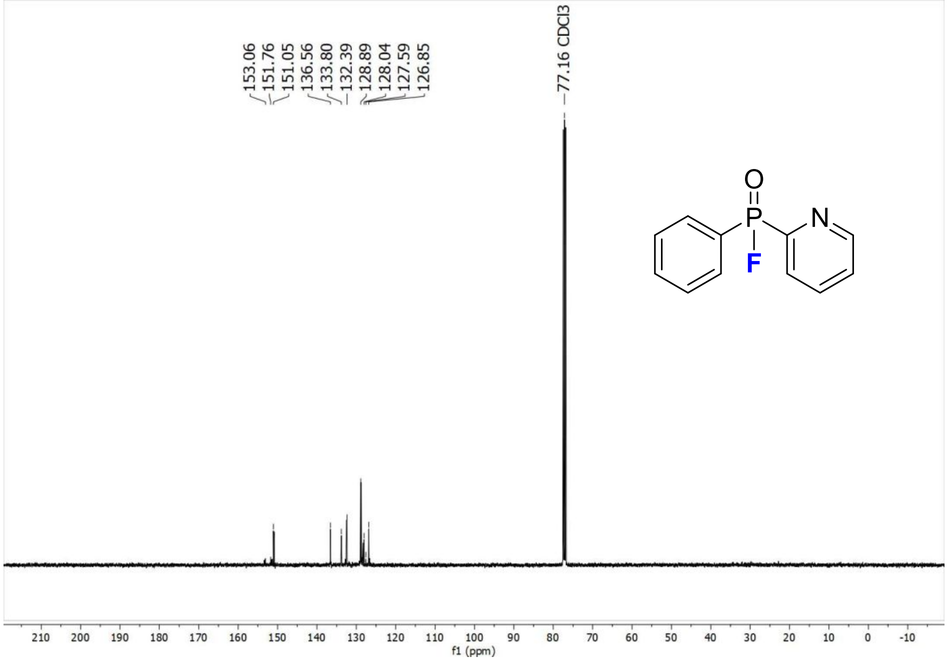

^{19}F NMR: 376 MHz in $CDCl_3$

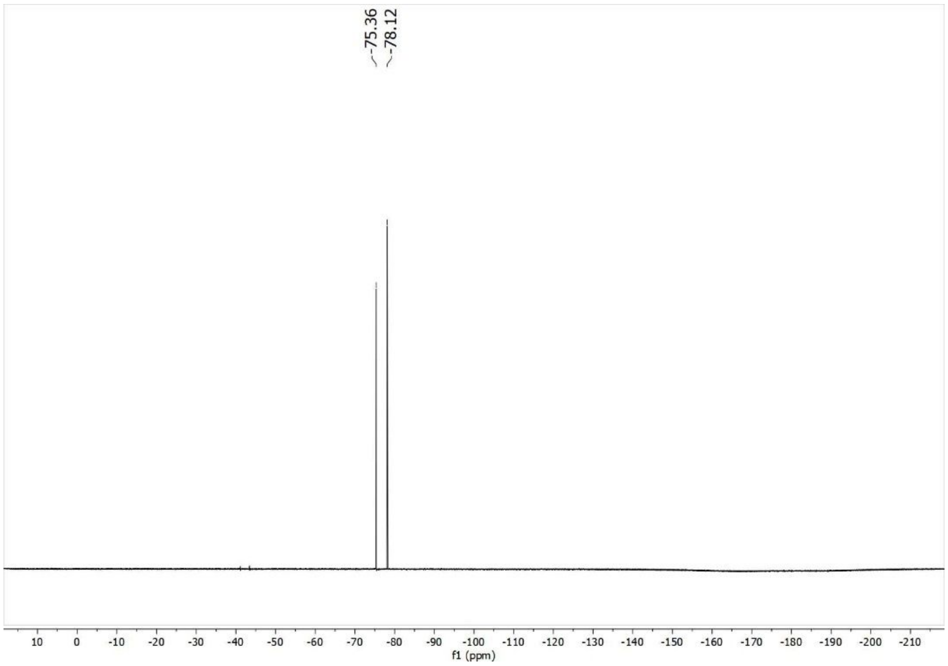

^{31}P NMR: 161 MHz in $CDCl_3$

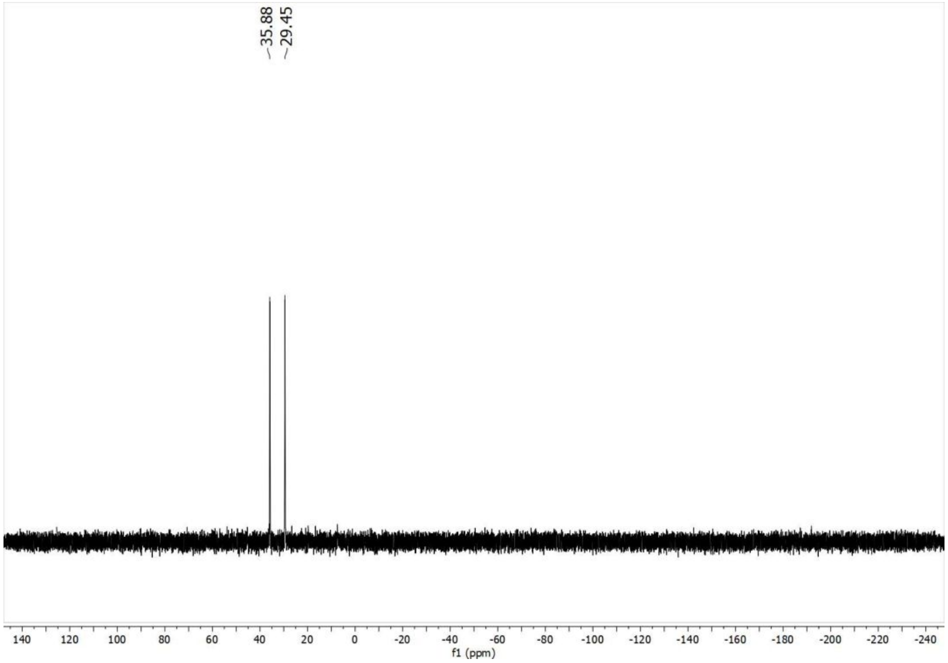

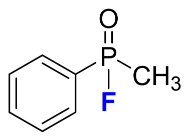

(methyl)(phenyl)phosphinic fluoride (2o).

^{1}H NMR: 400 MHz in $CDCl_3$

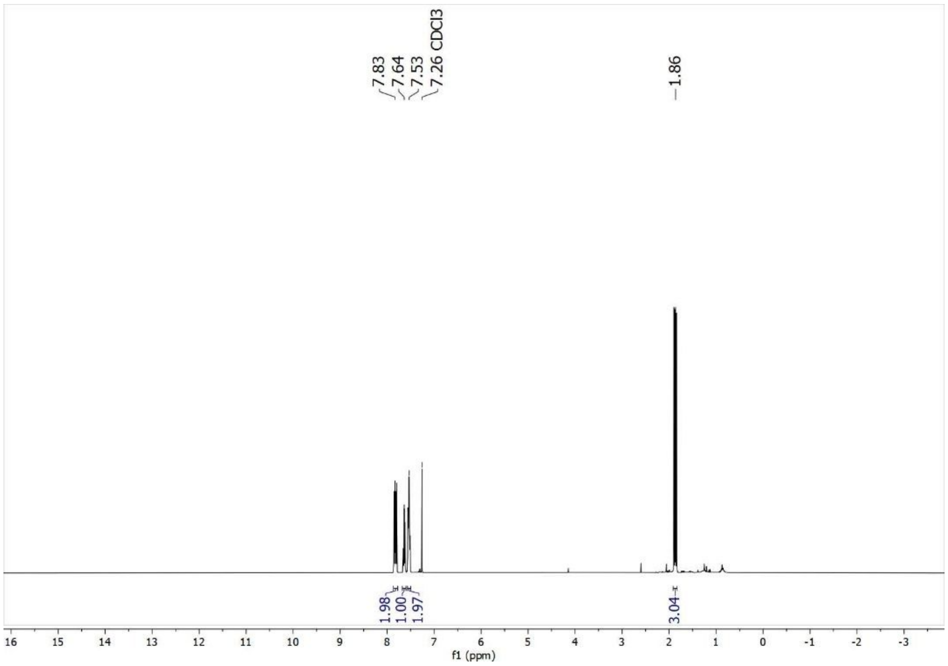

^{13}C NMR: 101 MHz in $CDCl_3$

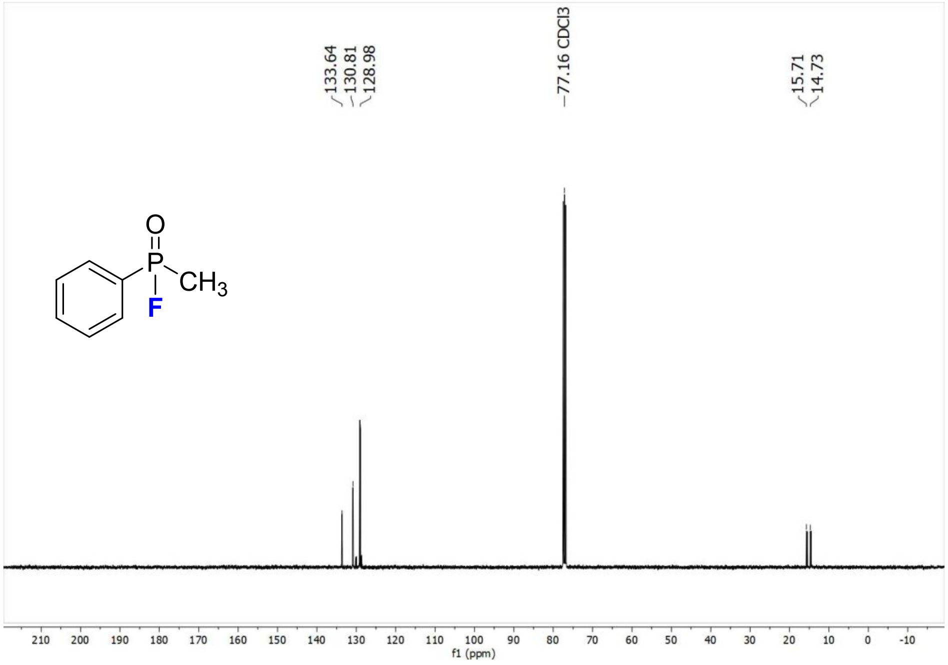

^{19}F NMR: 376 MHz in $CDCl_3$

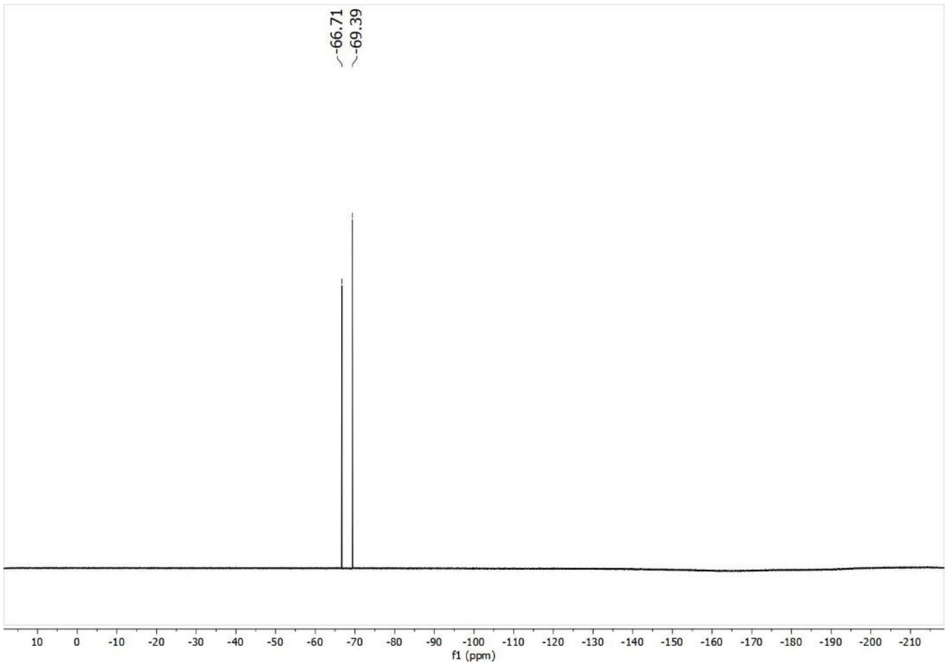

^{31}P NMR: 161 MHz in $CDCl_3$

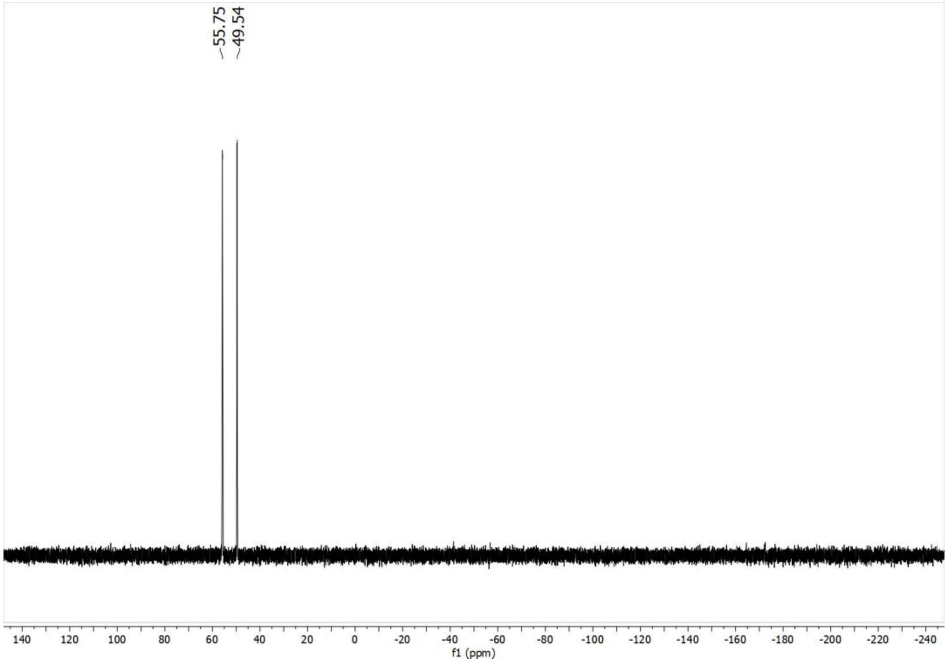

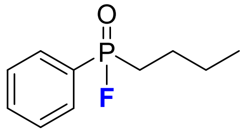

(*n*-butyl)(phenyl)phosphinic fluoride (2p).

^{1}H NMR: 400 MHz in $CDCl_3$

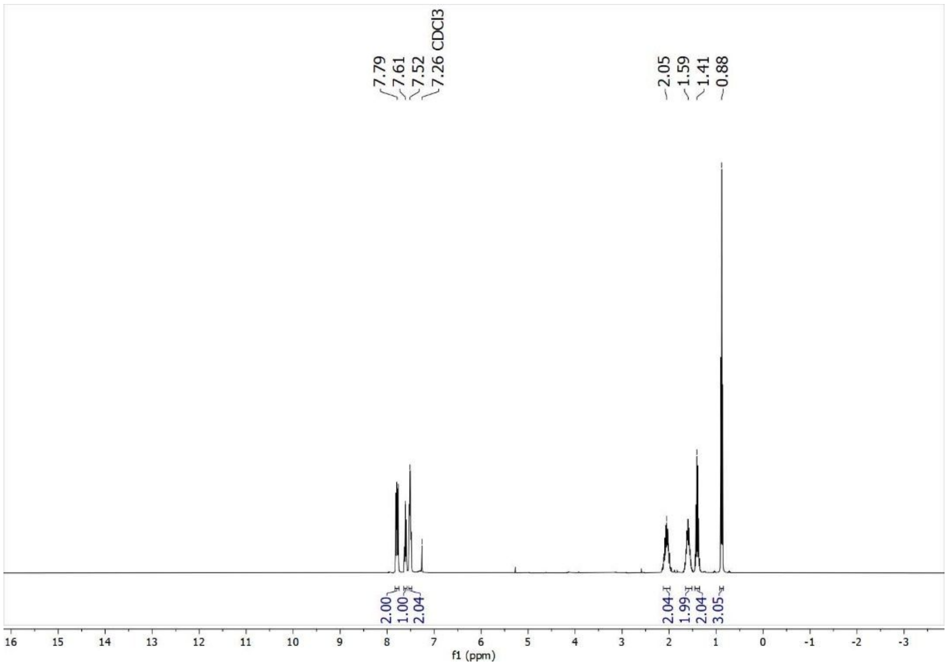

^{13}C NMR: 101 MHz in $CDCl_3$

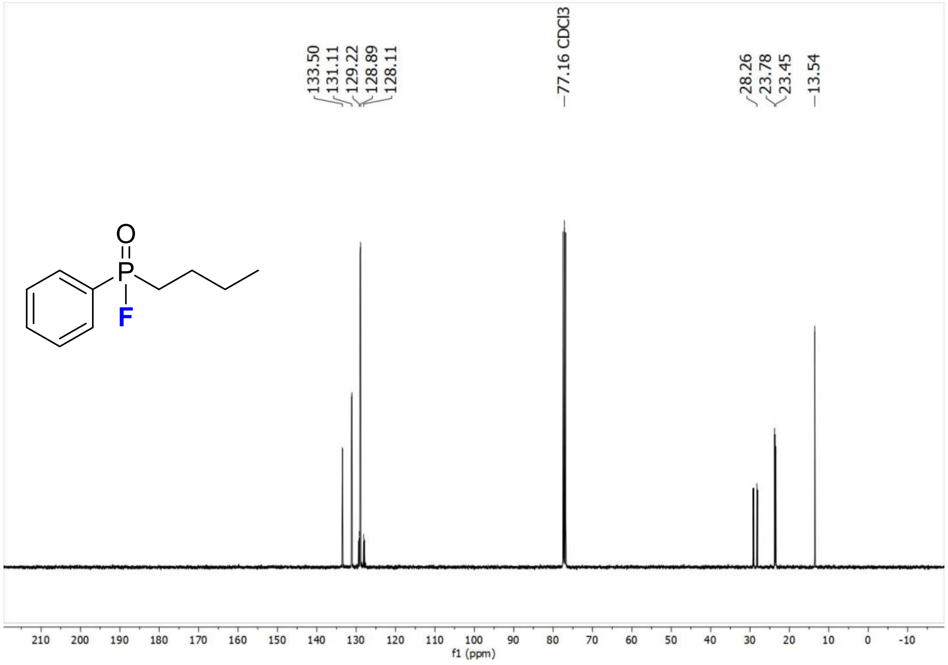

^{19}F NMR: 376 MHz in $CDCl_3$

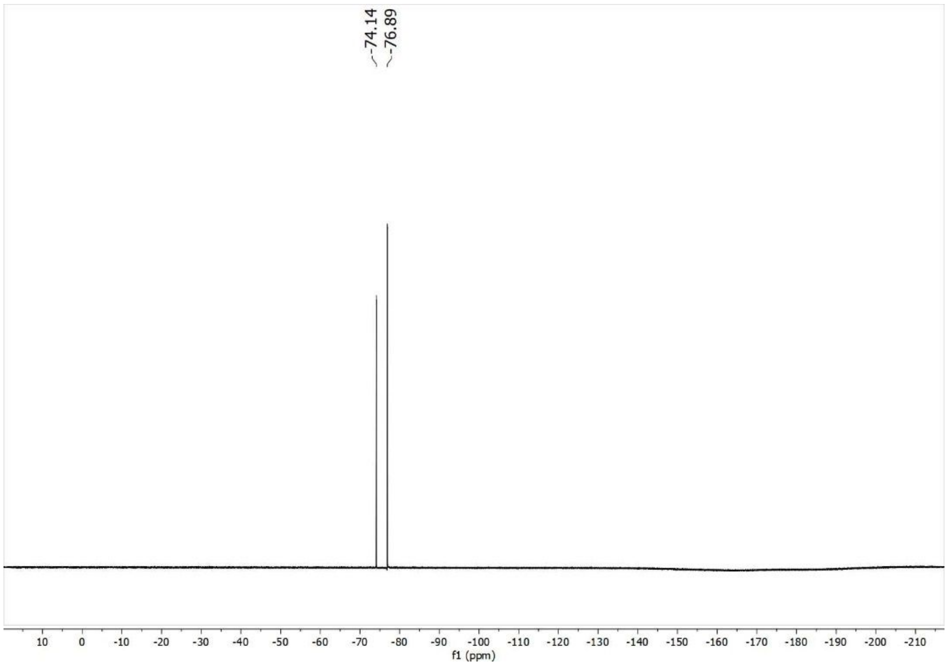

^{31}P NMR: 161 MHz in $CDCl_3$

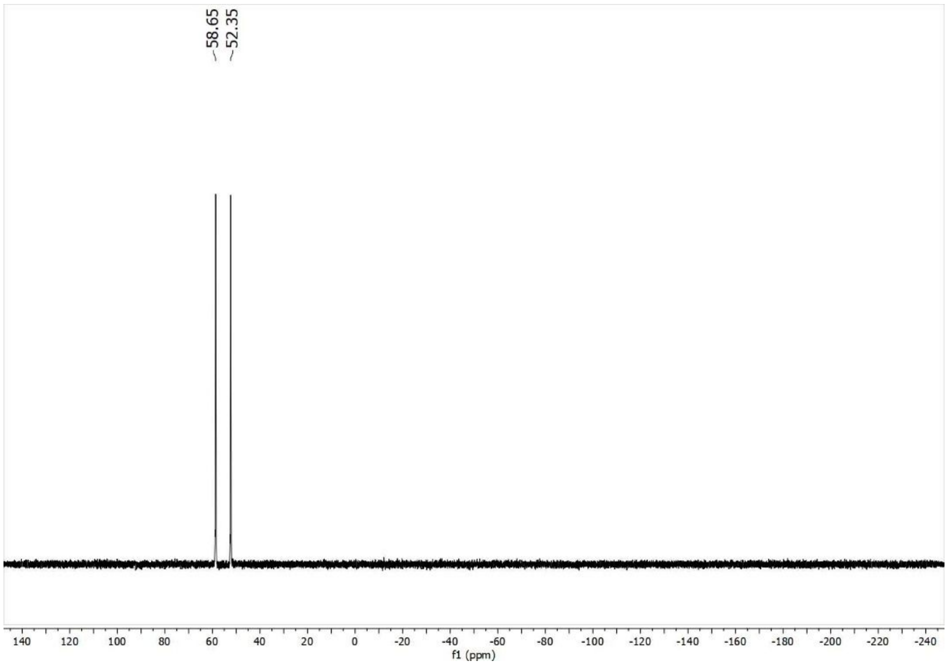

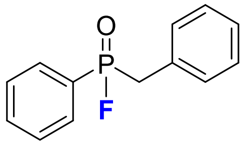

(benzyl)(phenyl)phosphinic fluoride (2q).

^{1}H NMR: 400 MHz in $CDCl_3$

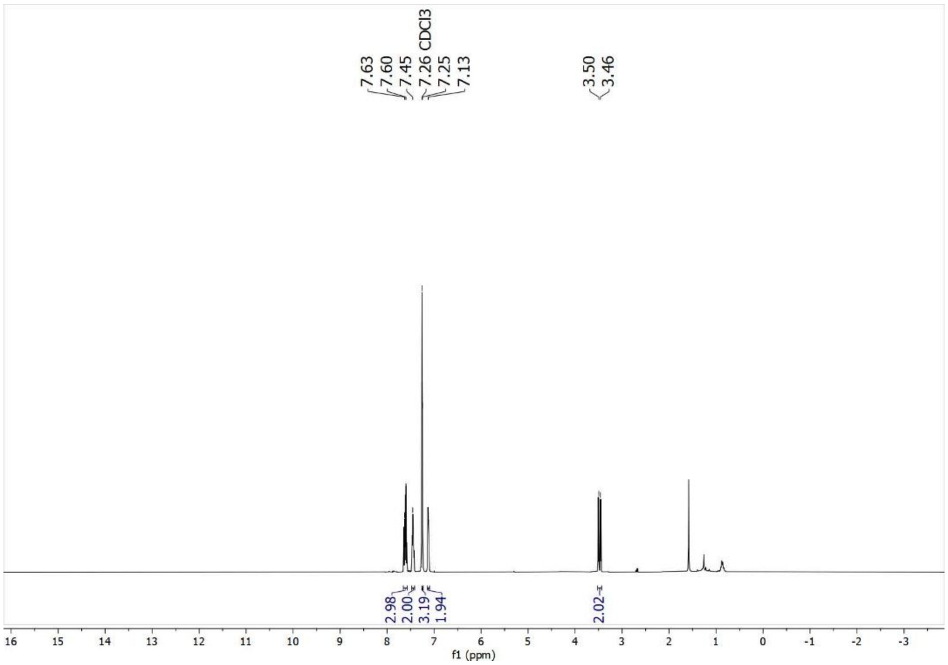

^{13}C NMR: 101 MHz in $CDCl_3$

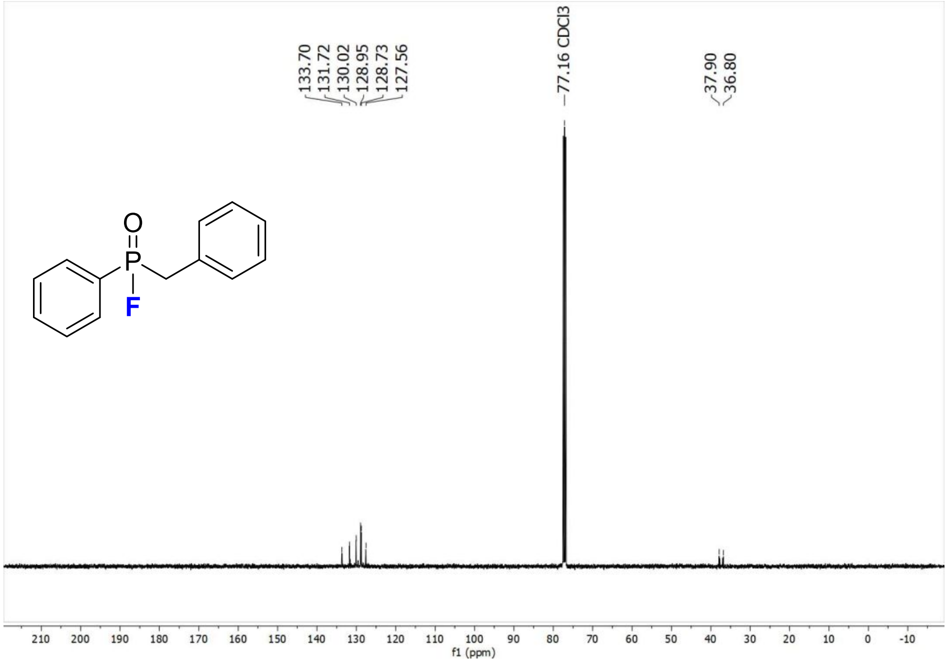

^{19}F NMR: 376 MHz in $CDCl_3$

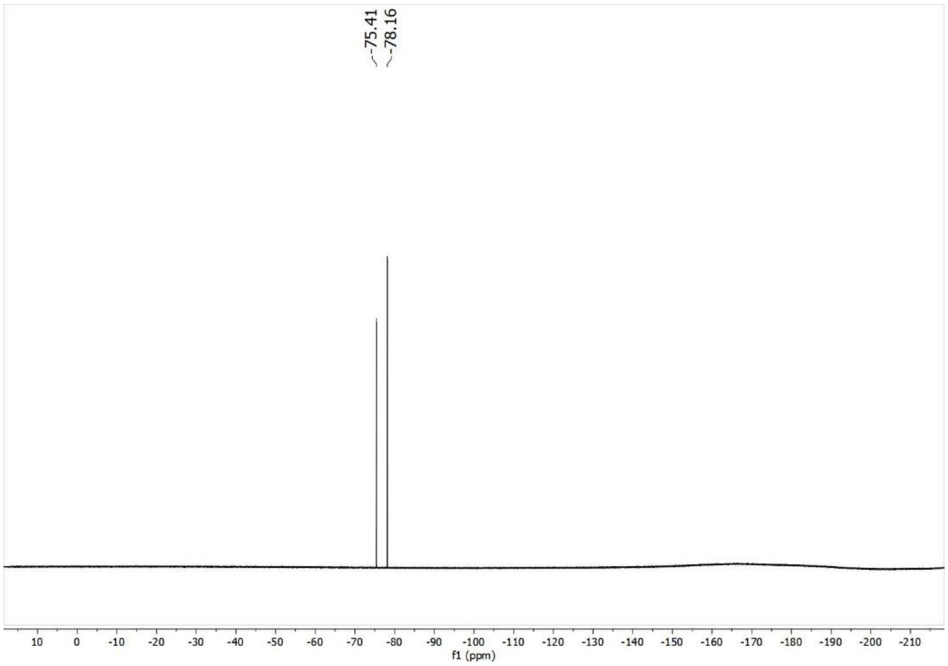

^{31}P NMR: 161 MHz in $CDCl_3$

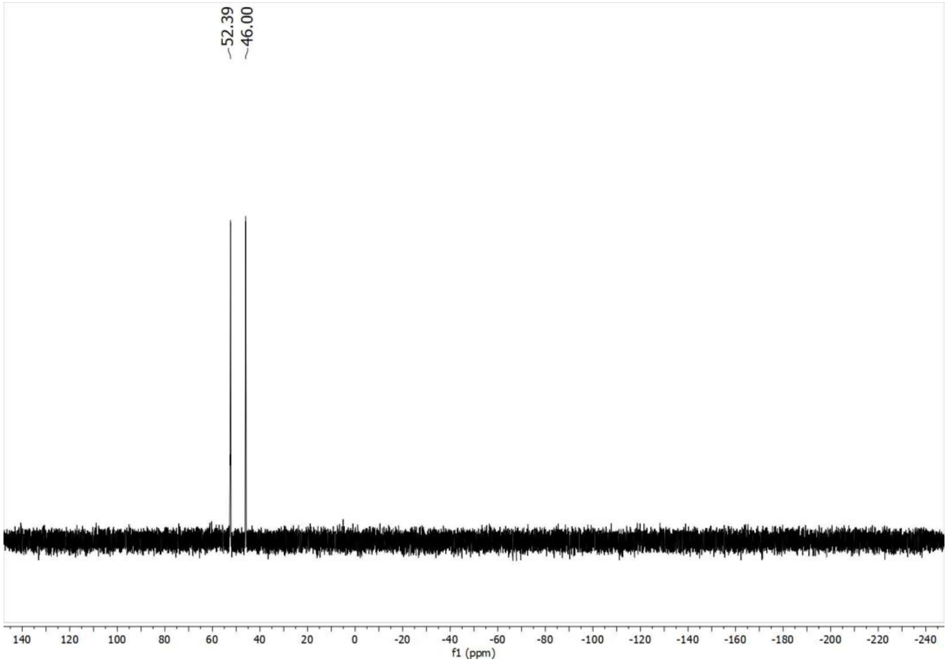

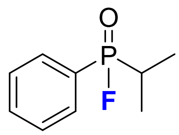

(*iso*-propyl)(phenyl)phosphinic fluoride (2r).

^{1}H NMR: 400 MHz in $CDCl_3$

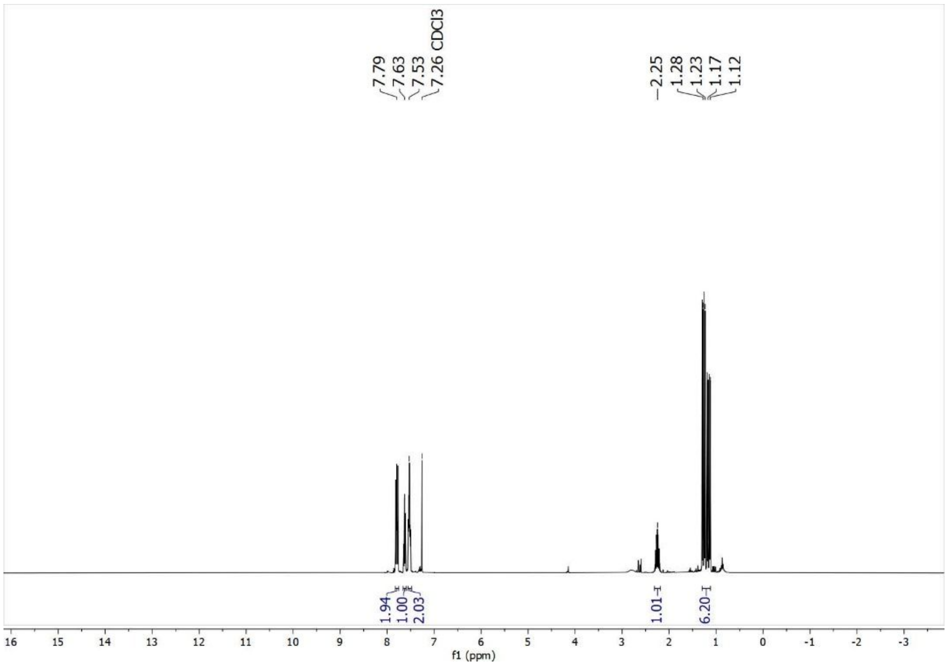

^{13}C NMR: 101 MHz in $CDCl_3$

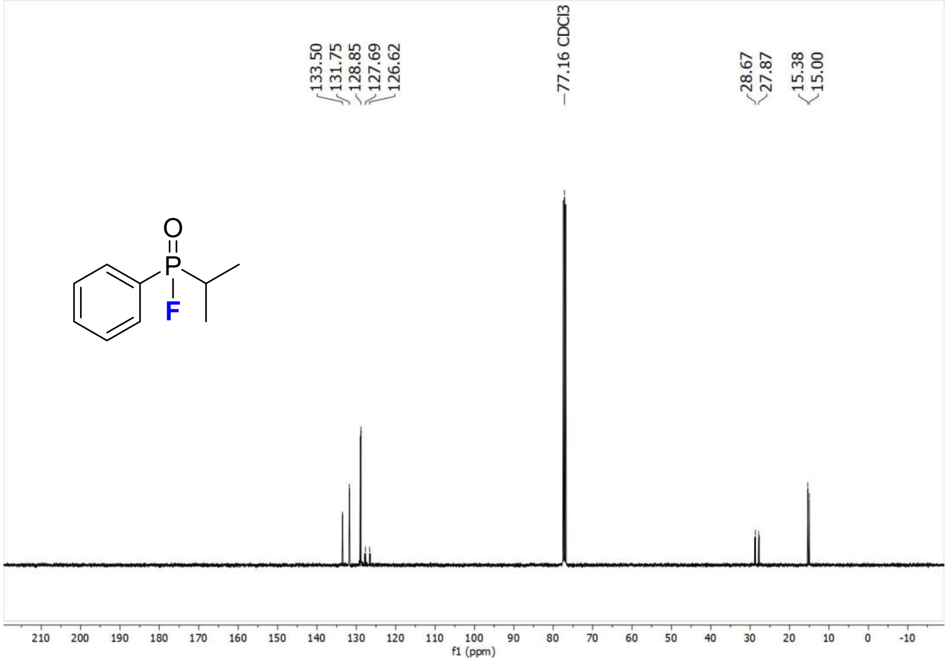

^{19}F NMR: 376 MHz in $CDCl_3$

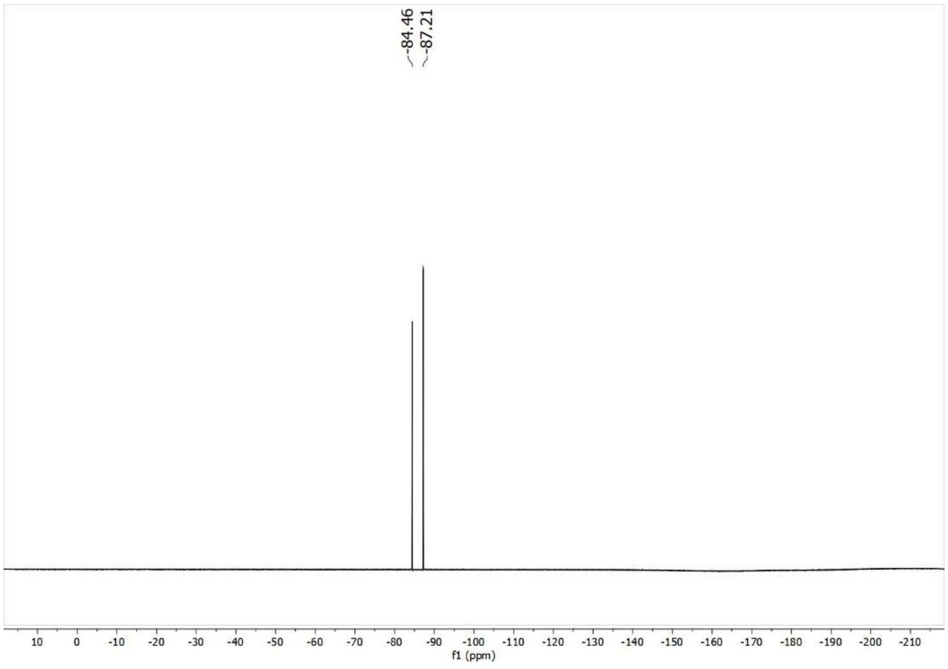

^{31}P NMR: 161 MHz in $CDCl_3$

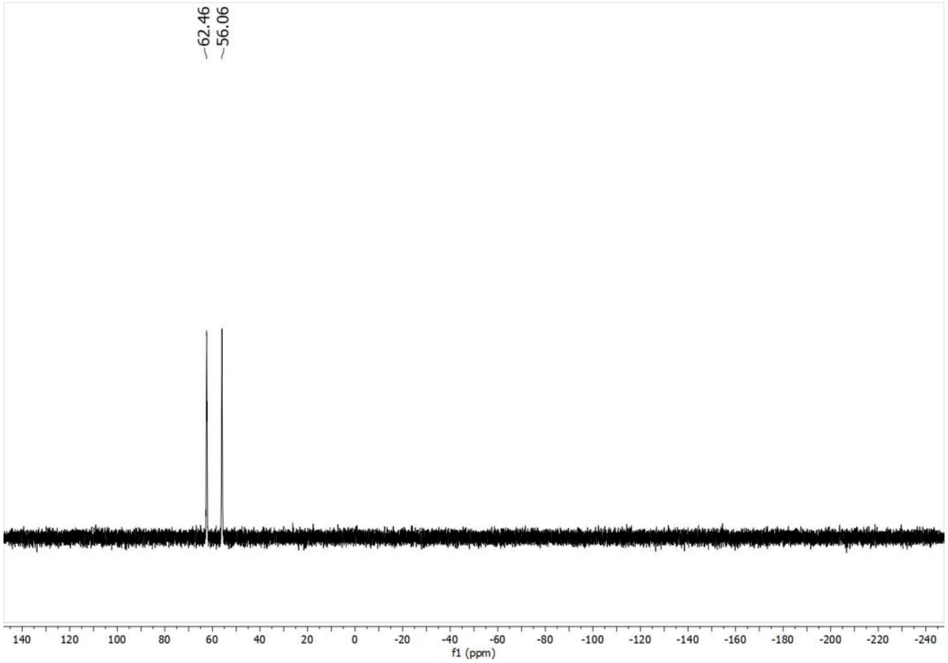

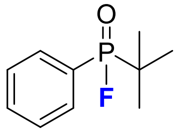

(*tert*-butyl)(phenyl)phosphinic fluoride (2s).

^{1}H NMR: 400 MHz in $CDCl_3$

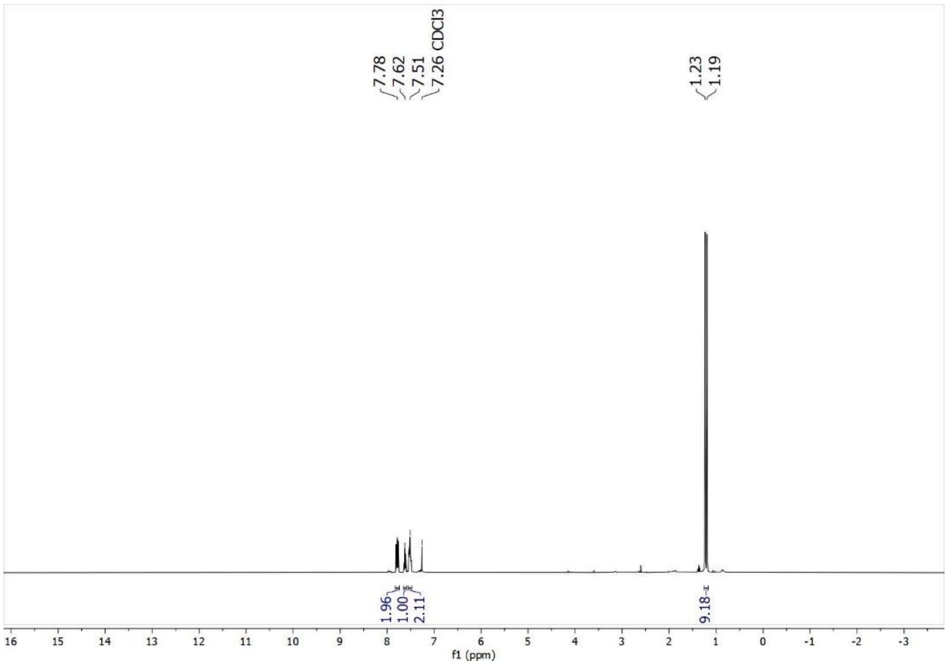

^{13}C NMR: 101 MHz in $CDCl_3$

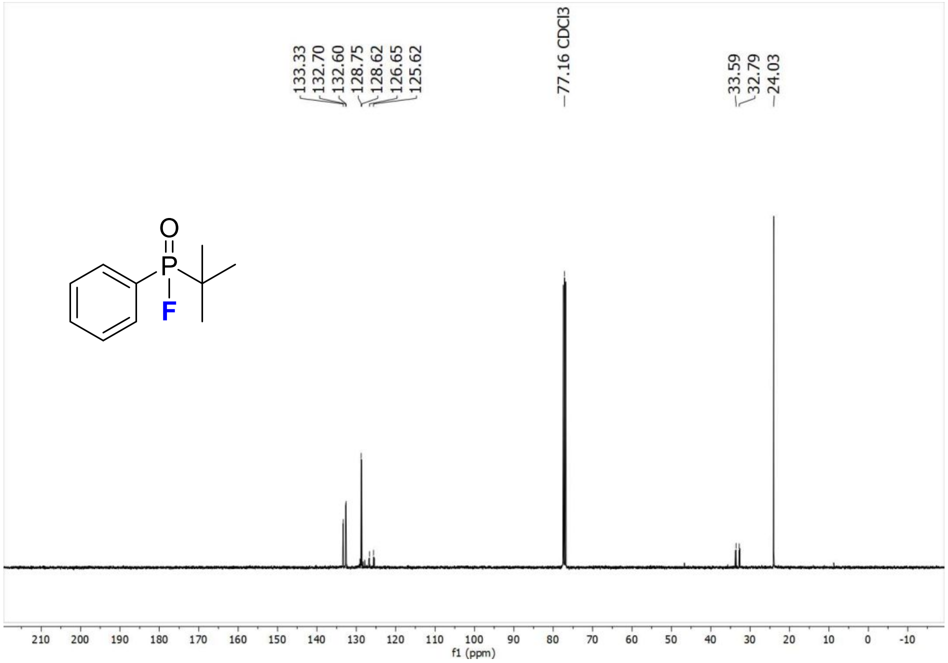

^{19}F NMR: 376 MHz in $CDCl_3$

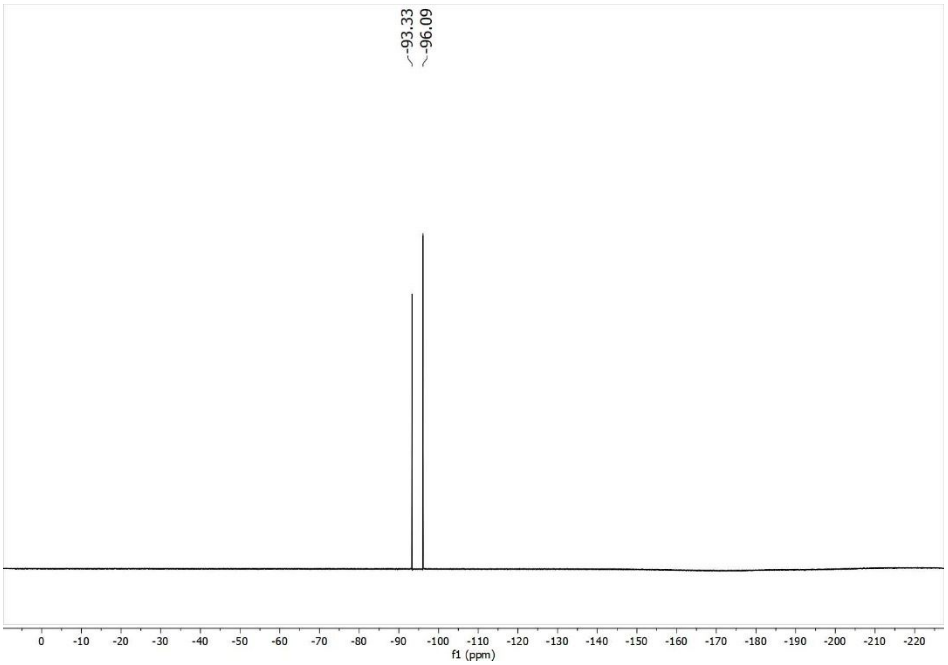

^{31}P NMR: 161 MHz in $CDCl_3$

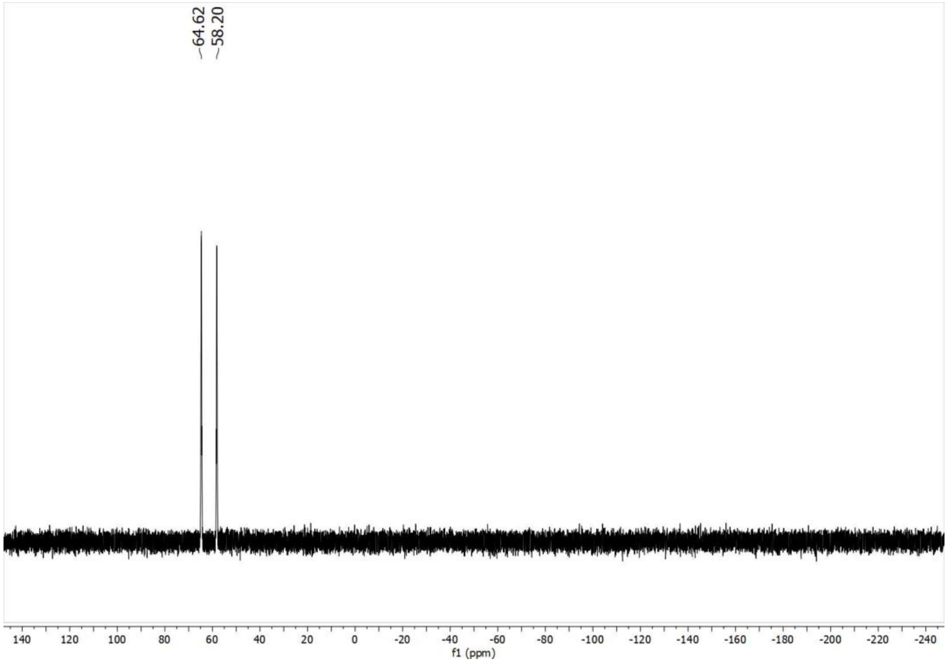

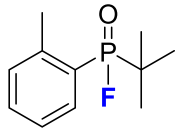

(*tert*-butyl)(2-methylphenyl)phosphinic fluoride (2t).

^{1}H NMR: 400 MHz in $CDCl_3$

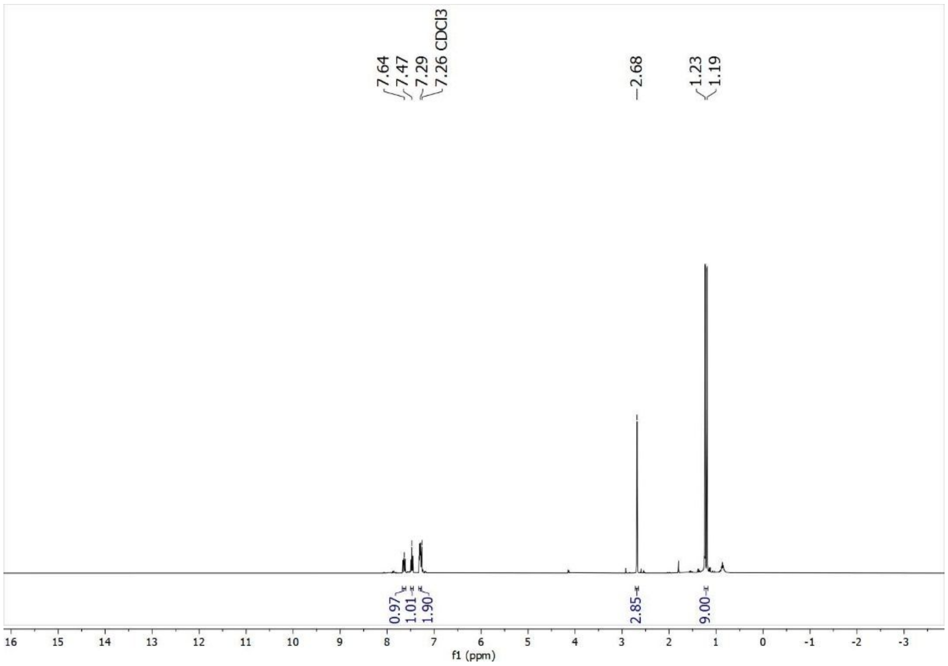

^{13}C NMR: 101 MHz in $CDCl_3$

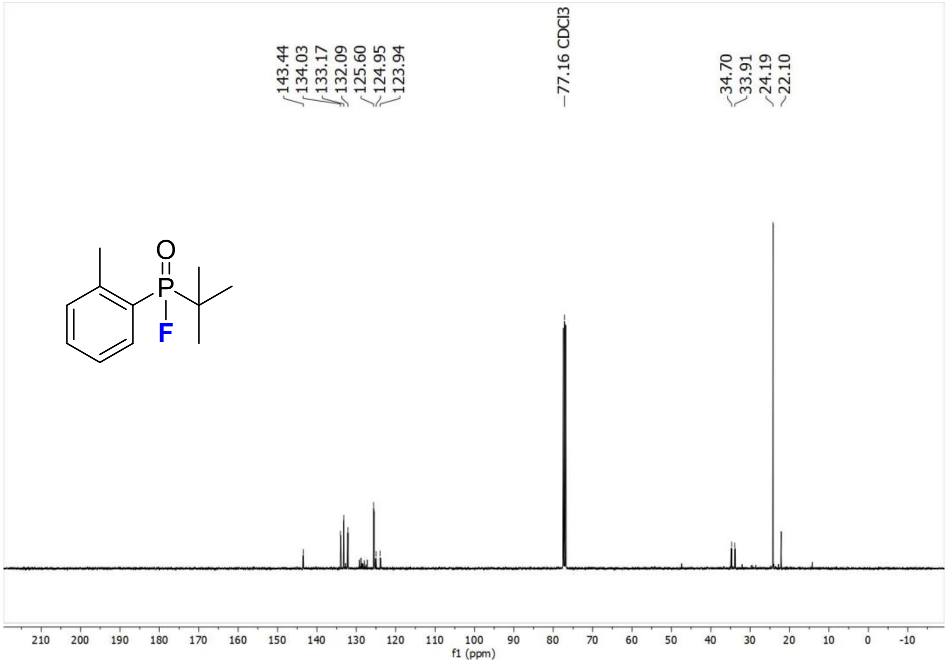

^{19}F NMR: 376 MHz in $CDCl_3$

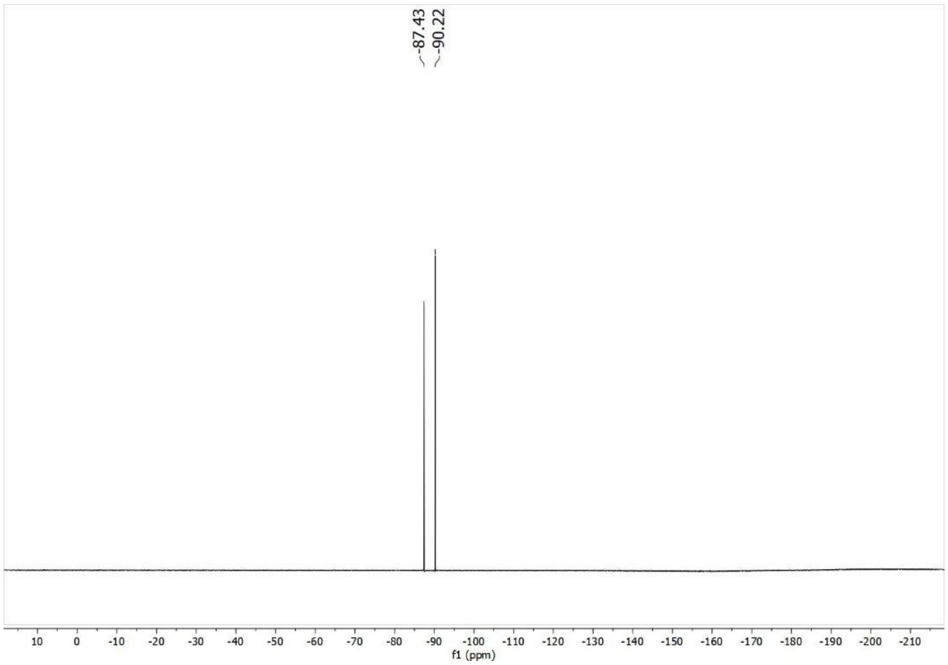

^{31}P NMR: 161 MHz in $CDCl_3$

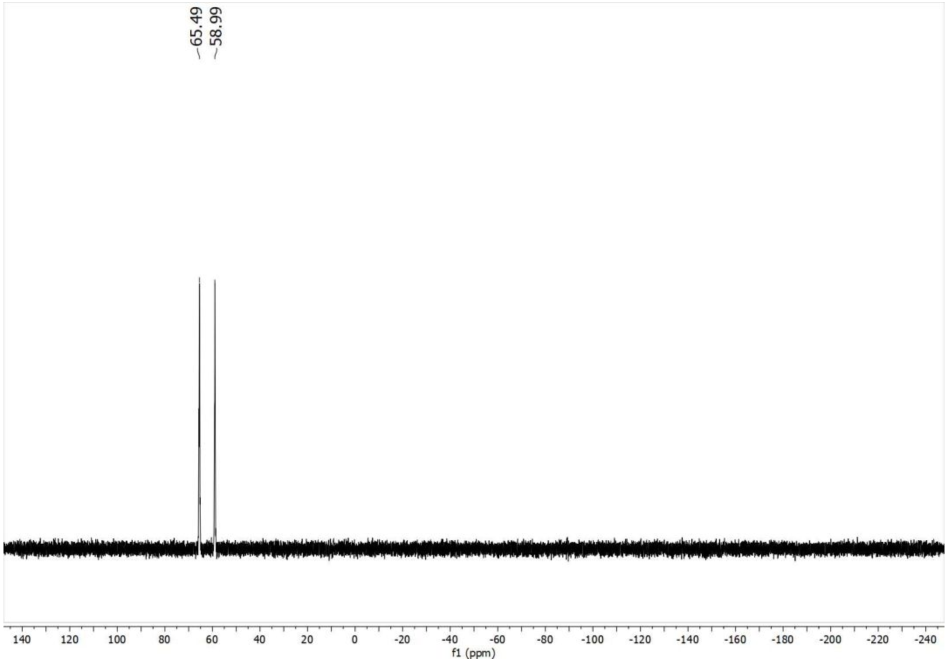

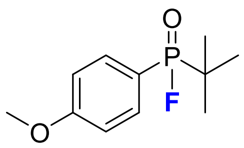

(*tert*-butyl)(4-methoxyphenyl)phosphinic fluoride (2u).

^{1}H NMR: 400 MHz in $CDCl_3$

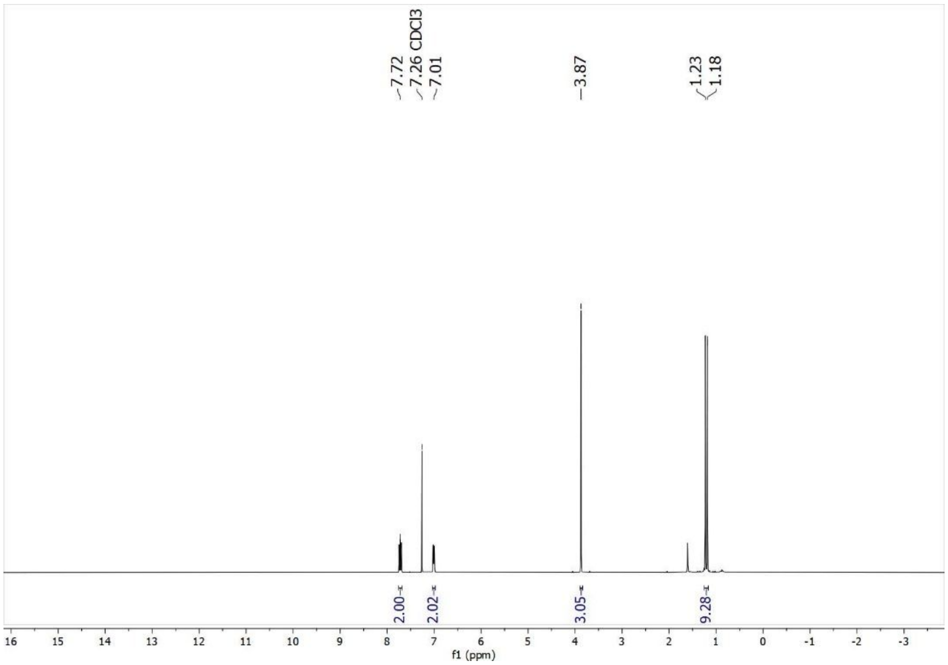

^{13}C NMR: 101 MHz in $CDCl_3$

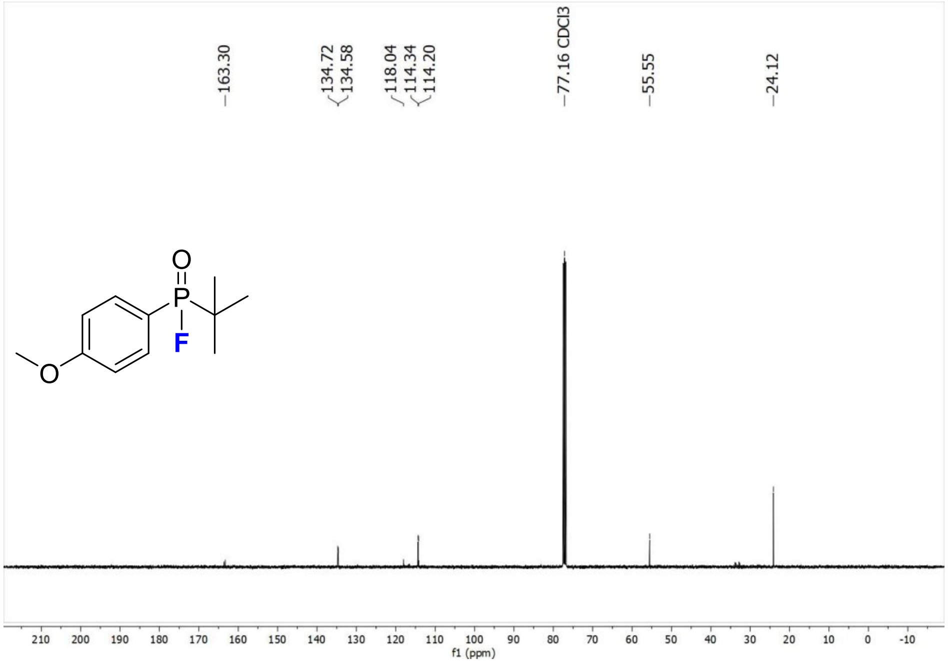

^{19}F NMR: 376 MHz in $CDCl_3$

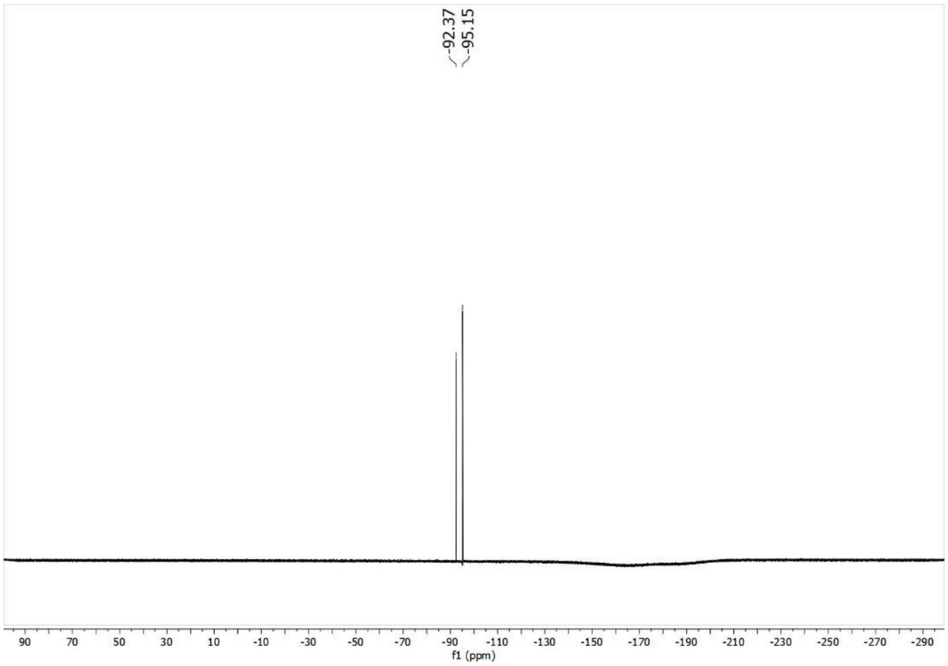

^{31}P NMR: 161 MHz in $CDCl_3$

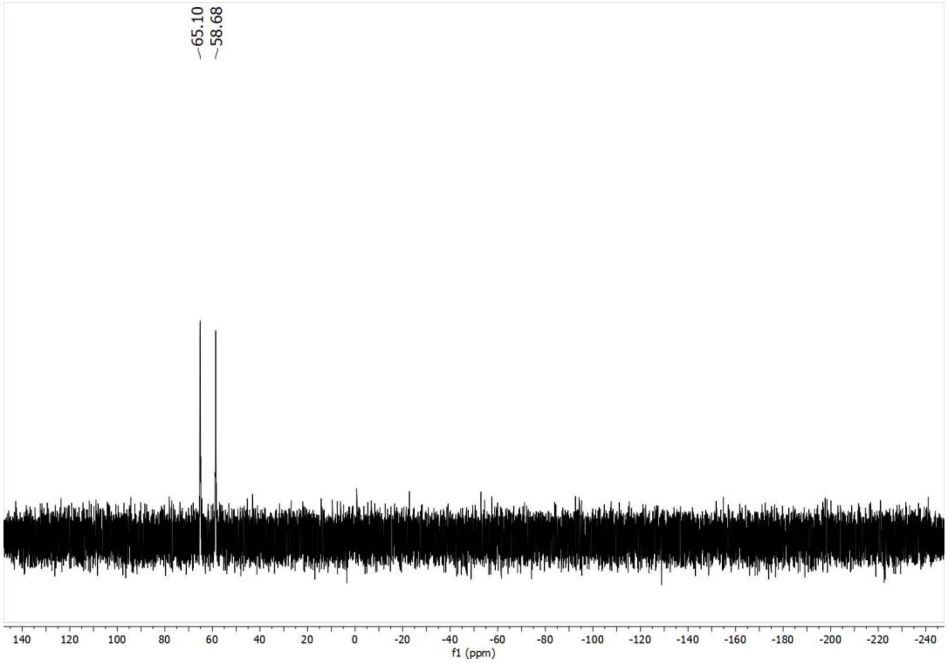

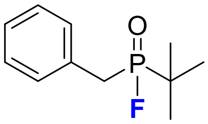

(*tert*-butyl)(benzyl)phosphinic fluoride (2v).

^{1}H NMR: 400 MHz in $CDCl_3$

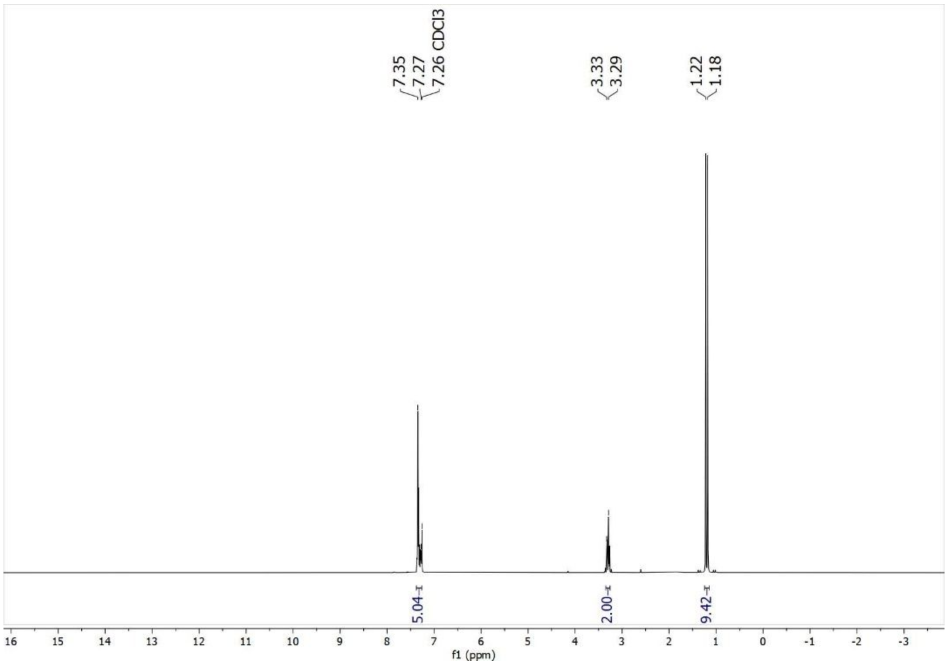

^{13}C NMR: 101 MHz in $CDCl_3$

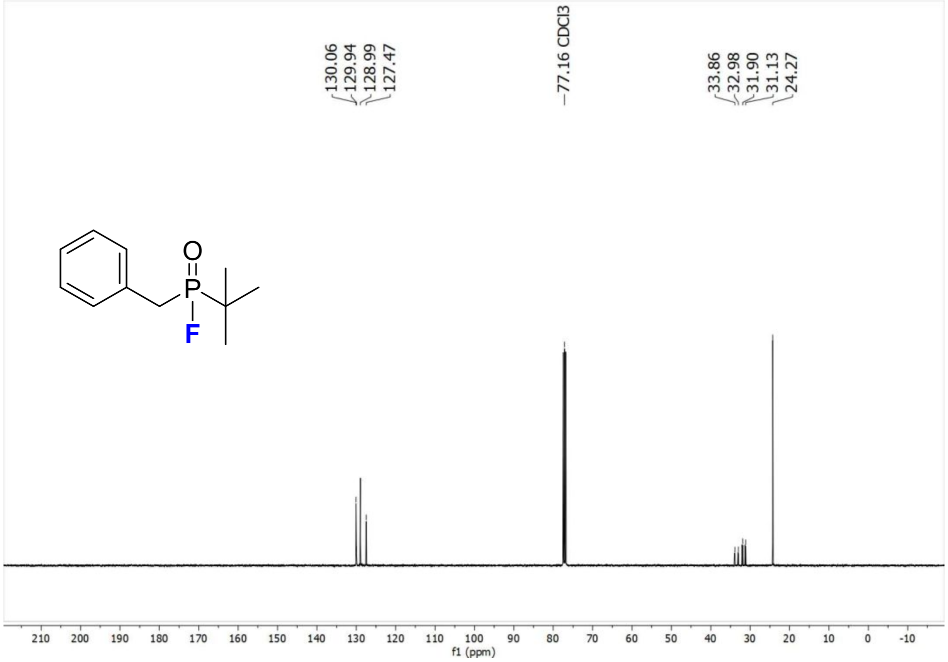

^{19}F NMR: 376 MHz in $CDCl_3$

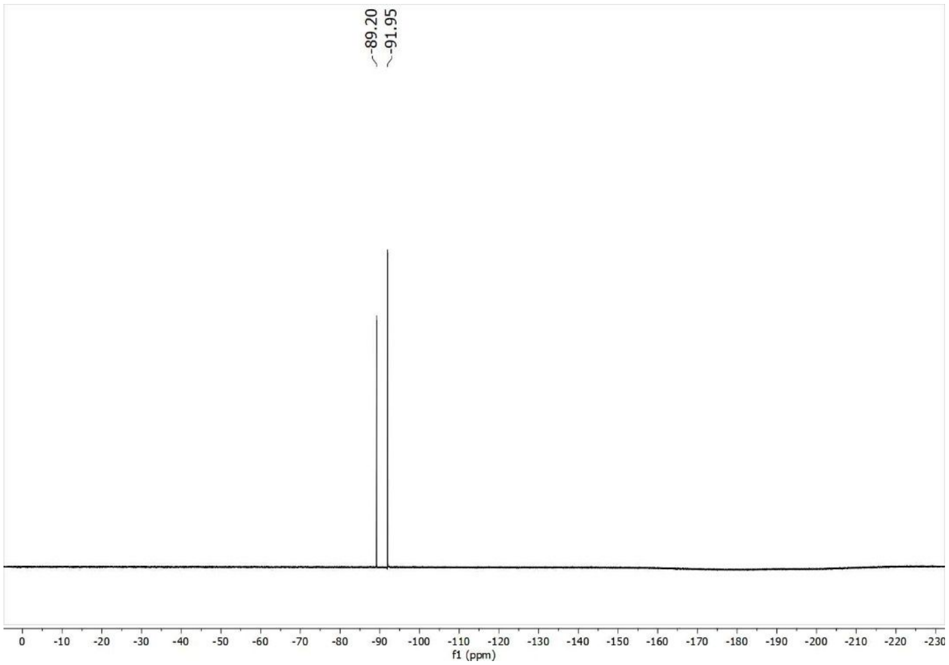

^{31}P NMR: 161 MHz in $CDCl_3$

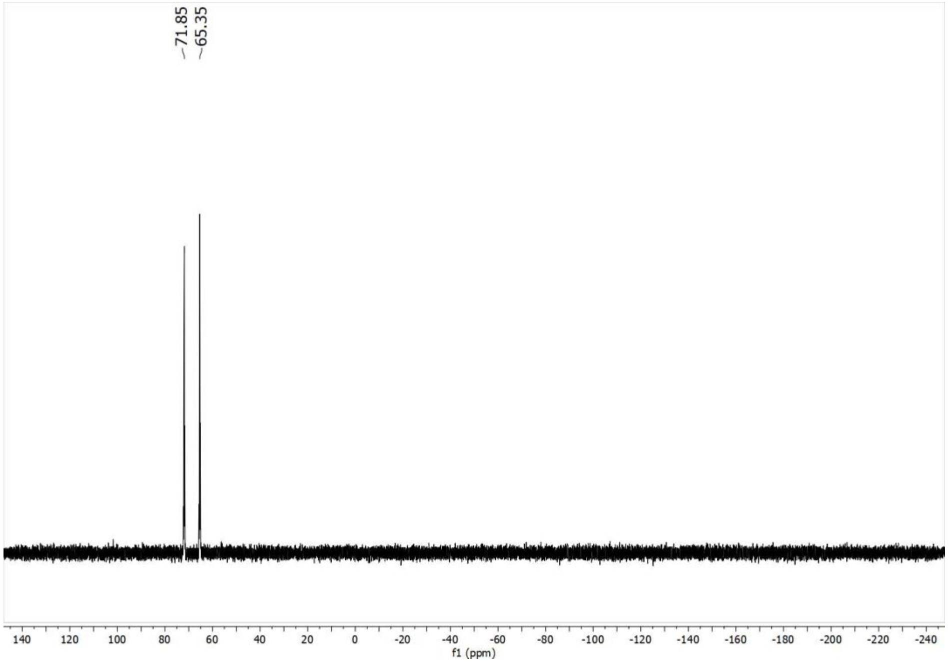

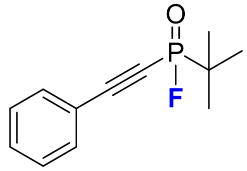

(*tert*-butyl)([2-phenyl]ethenyl)phosphinic fluoride (2w).

^{1}H NMR: 400 MHz in $CDCl_3$

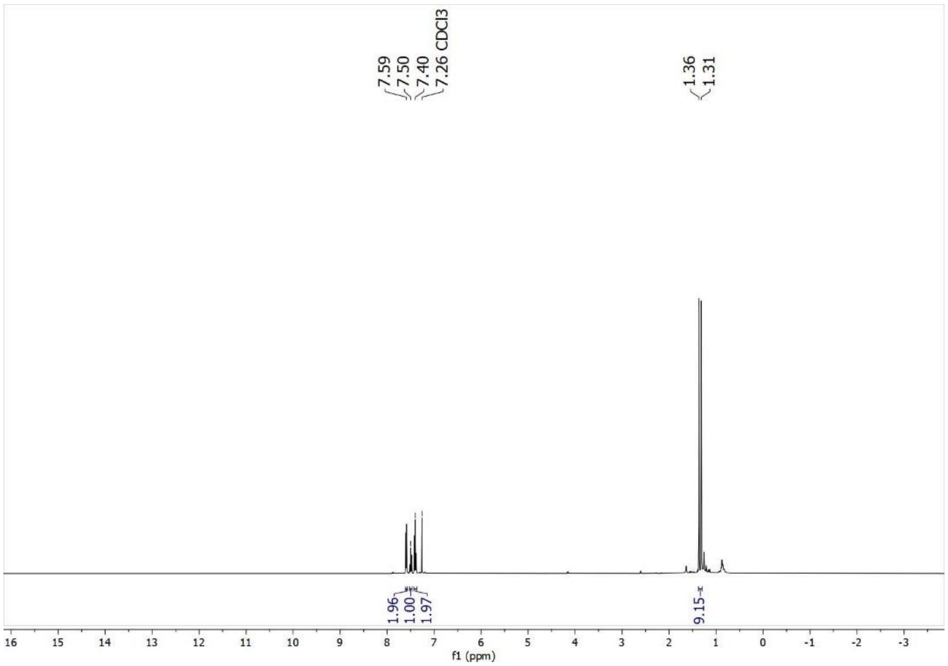

^{13}C NMR: 101 MHz in $CDCl_3$

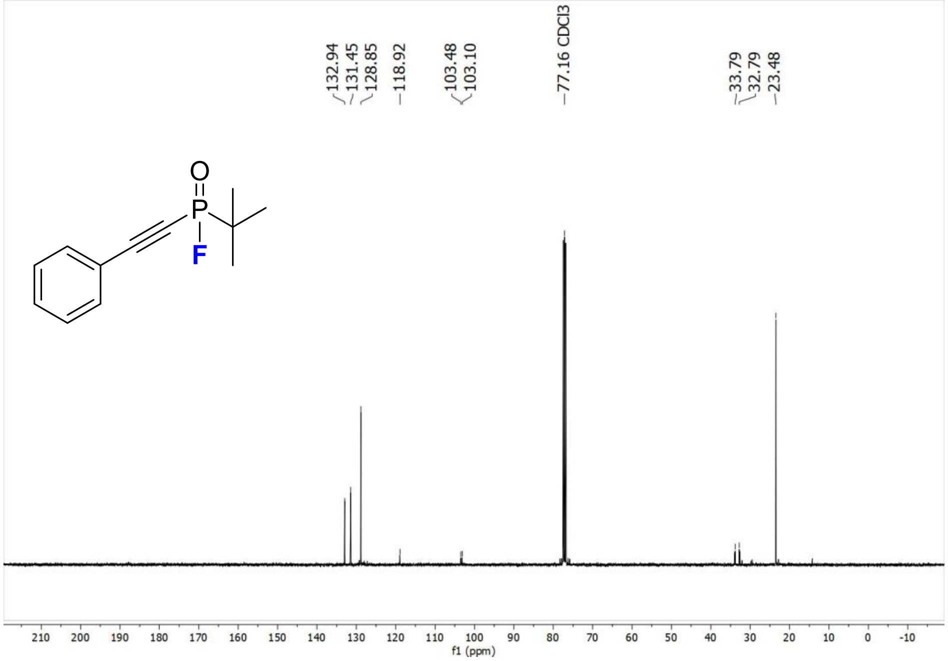

^{19}F NMR: 376 MHz in $CDCl_3$

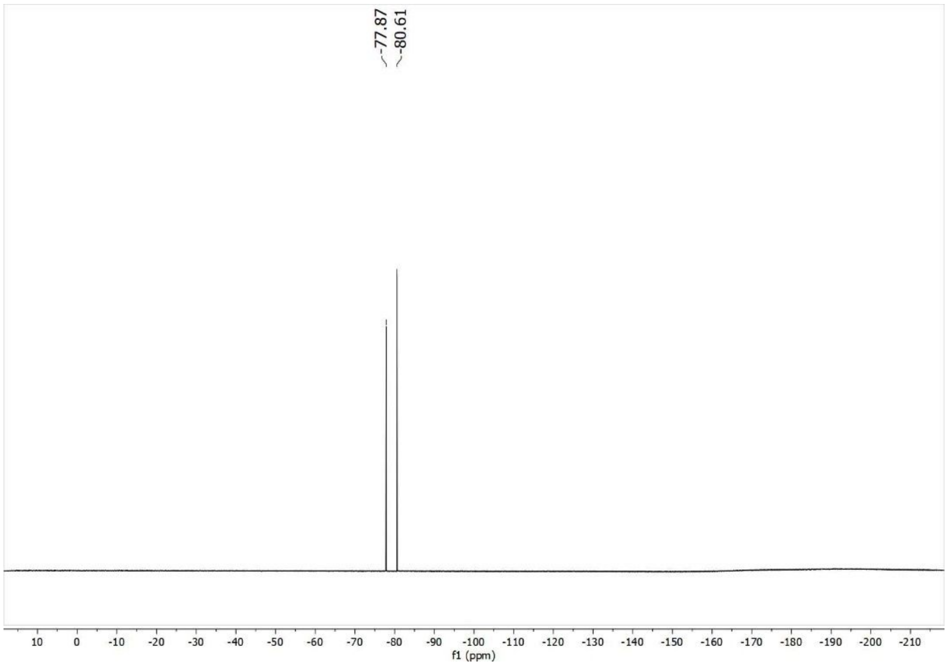

^{31}P NMR: 161 MHz in $CDCl_3$

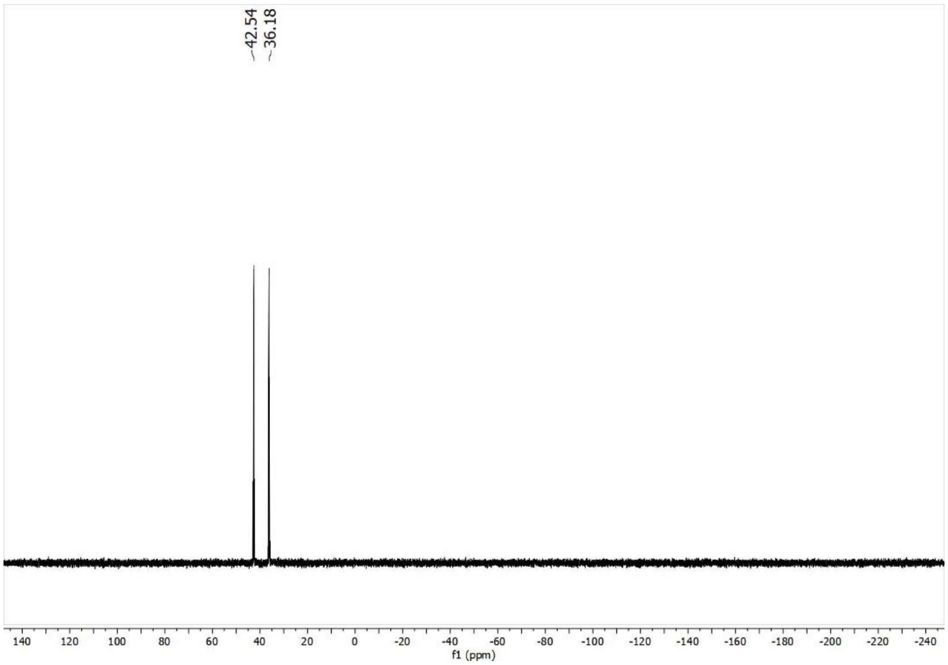

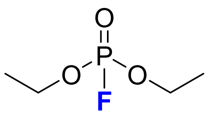

Bis(ethyl)fluorophosphate (2x).

Crude ^{19}F spectrum with 1.0 equivalents of 4-fluoroanisole (-125.29 ppm) as internal standard (60% yield): 376 MHz in CH_3CN

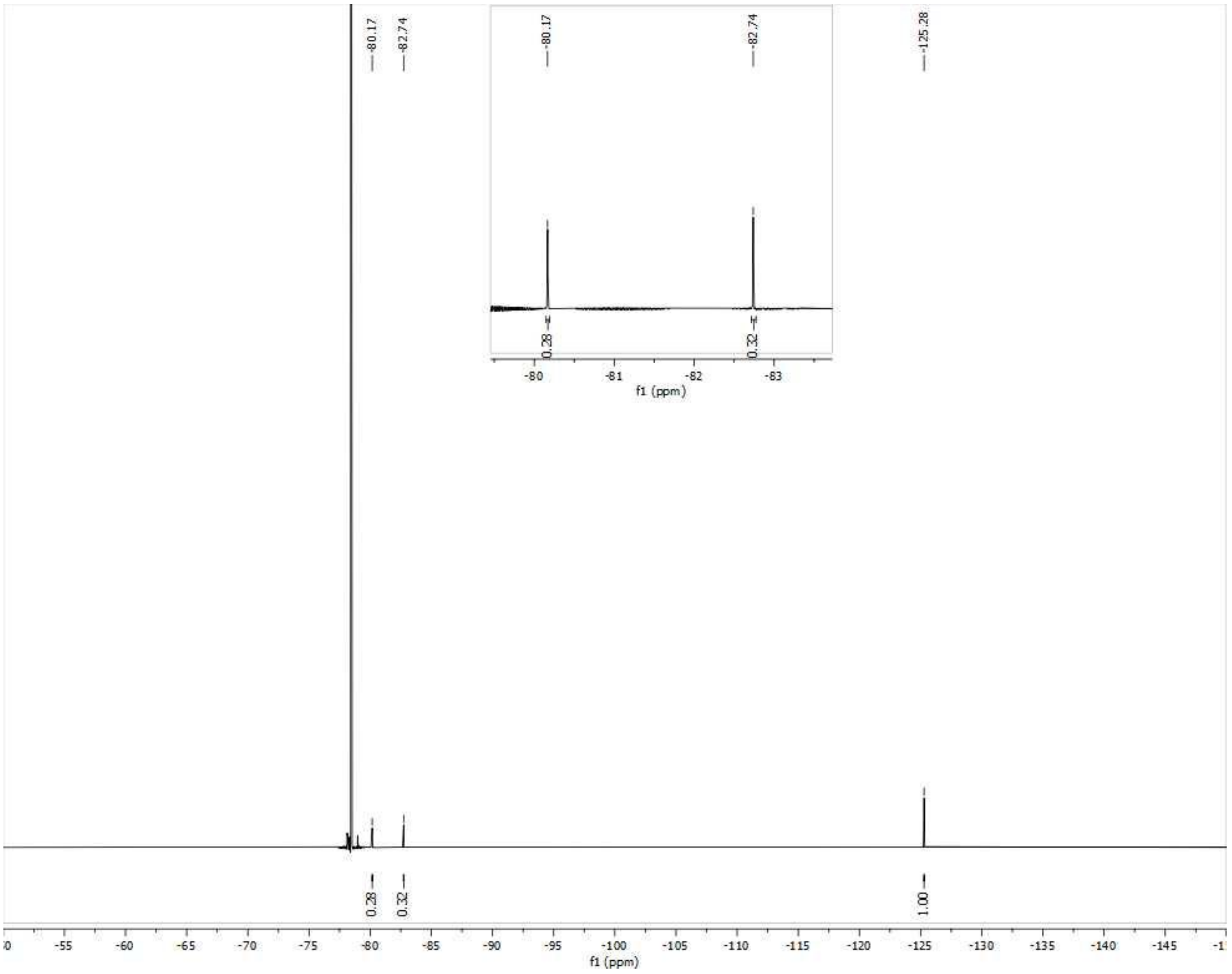

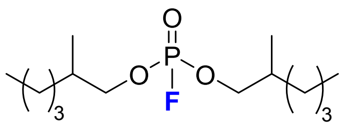

Bis(2-ethylhexyl)fluorophosphate (2y).

Crude ^{19}F spectrum with 1.0 equivalents of 4-fluoroanisole (-125.29 ppm) as internal standard (70% yield): 376 MHz in CH_3CN

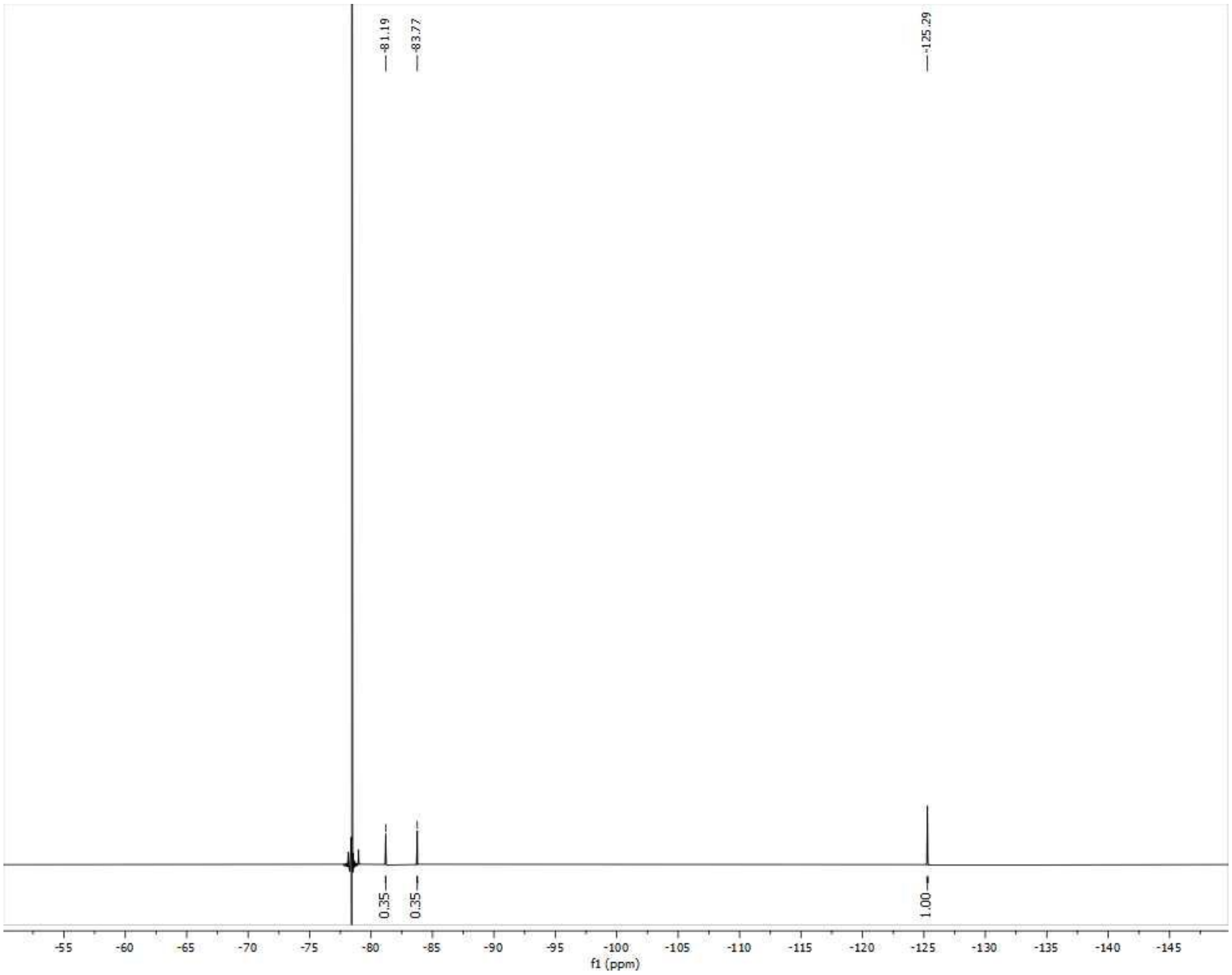

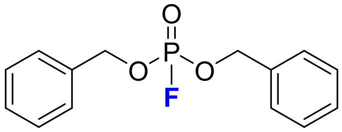

Bis(benzyl)fluorophosphate (2z).

Crude ^{19}F spectrum with 1.0 equivalents of 4-fluoroanisole (-125.29 ppm) as internal standard (48% yield): 376 MHz in CH_3CN

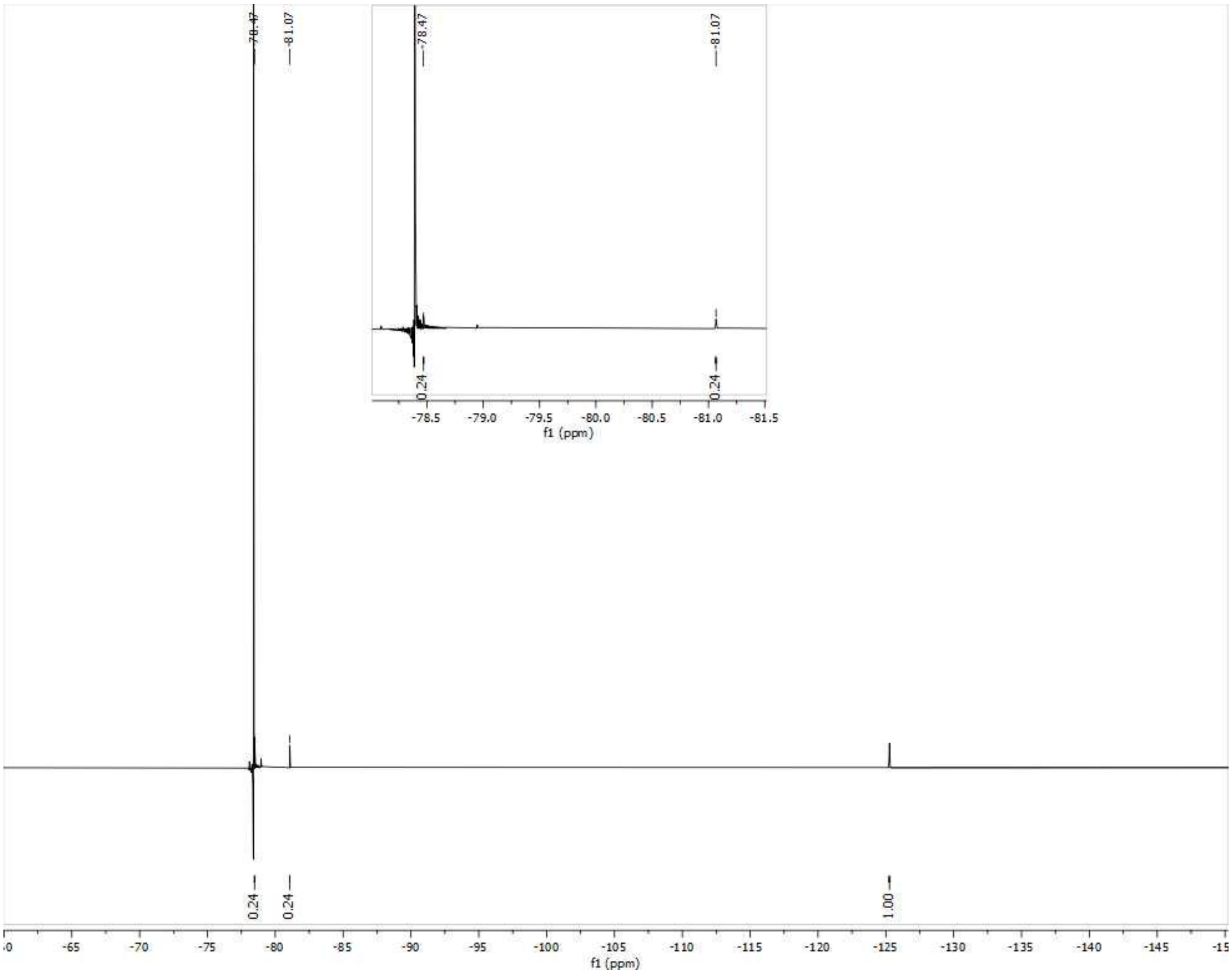

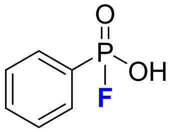

Phenyl phosphonofluoridic acid (2aa).

^{1}H NMR: 400 MHz in $CDCl_3$

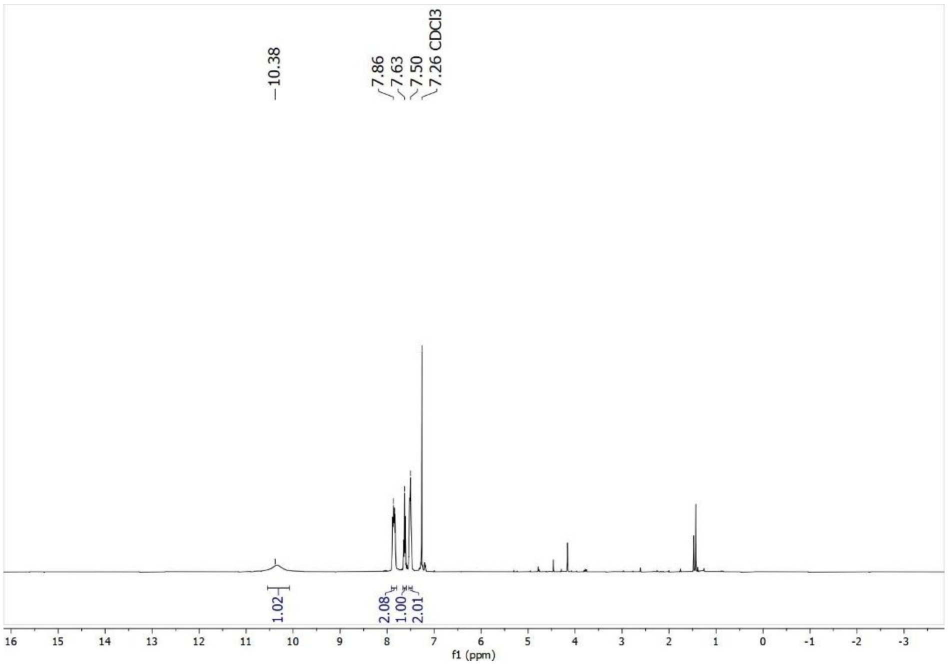

^{13}C NMR: 101 MHz in $CDCl_3$

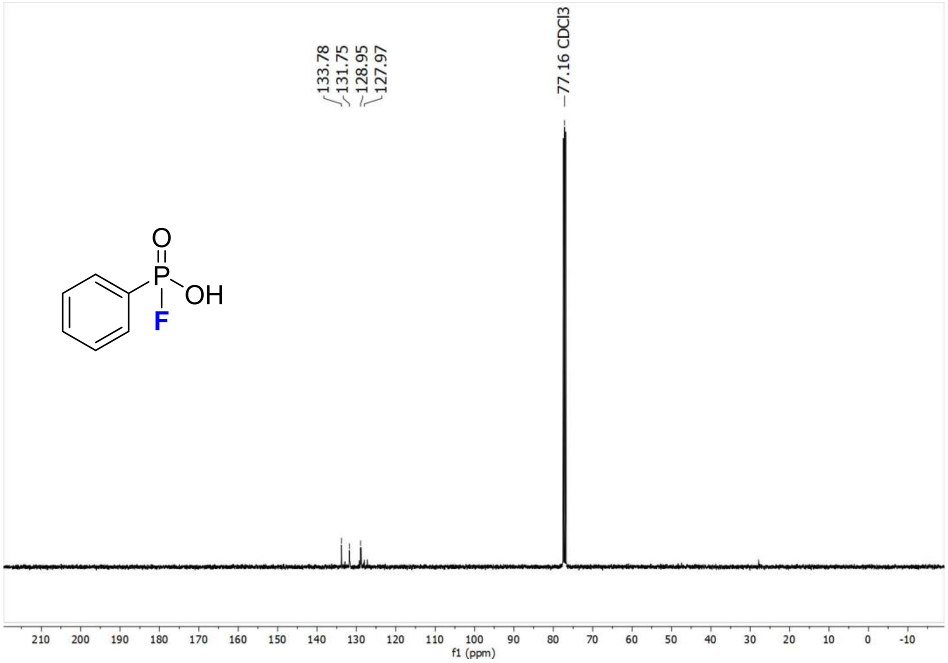

^{19}F NMR: 376 MHz in $CDCl_3$

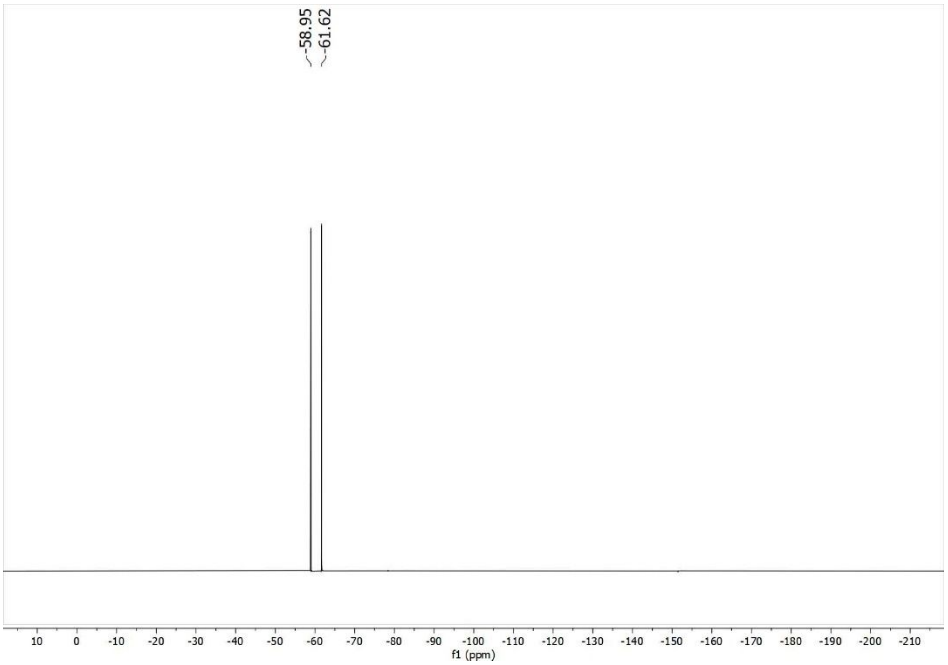

^{31}P NMR: 161 MHz in $CDCl_3$

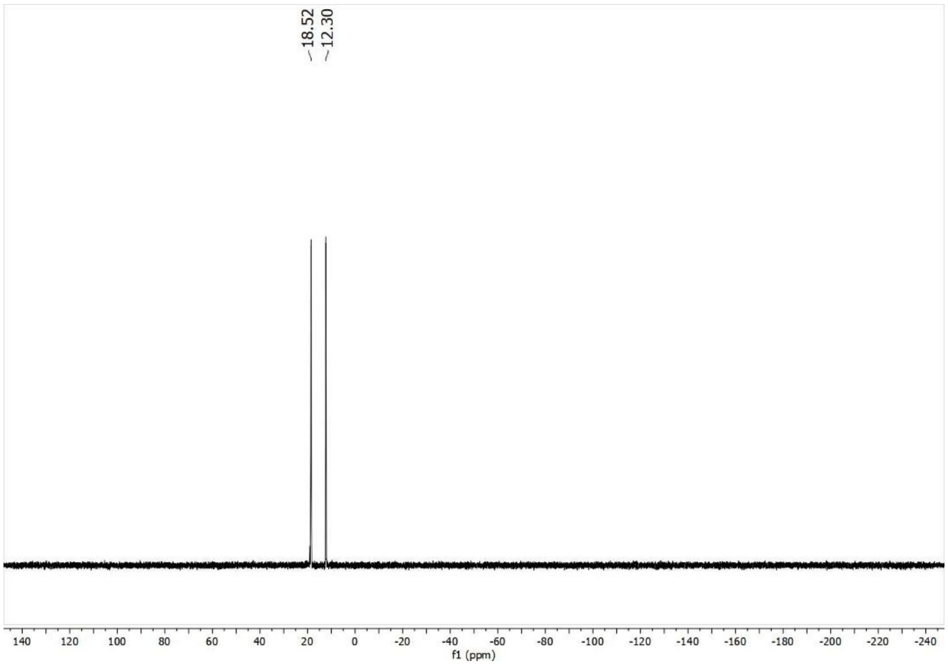

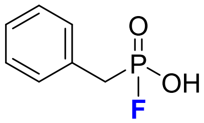

Benzyl phosphonofluoridic acid (2ab).

Crude ^{19}F spectrum with 1.0 equivalents of 4-fluoroanisole (-125.30 ppm) as internal standard (91% yield): 376 MHz in CH_3CN

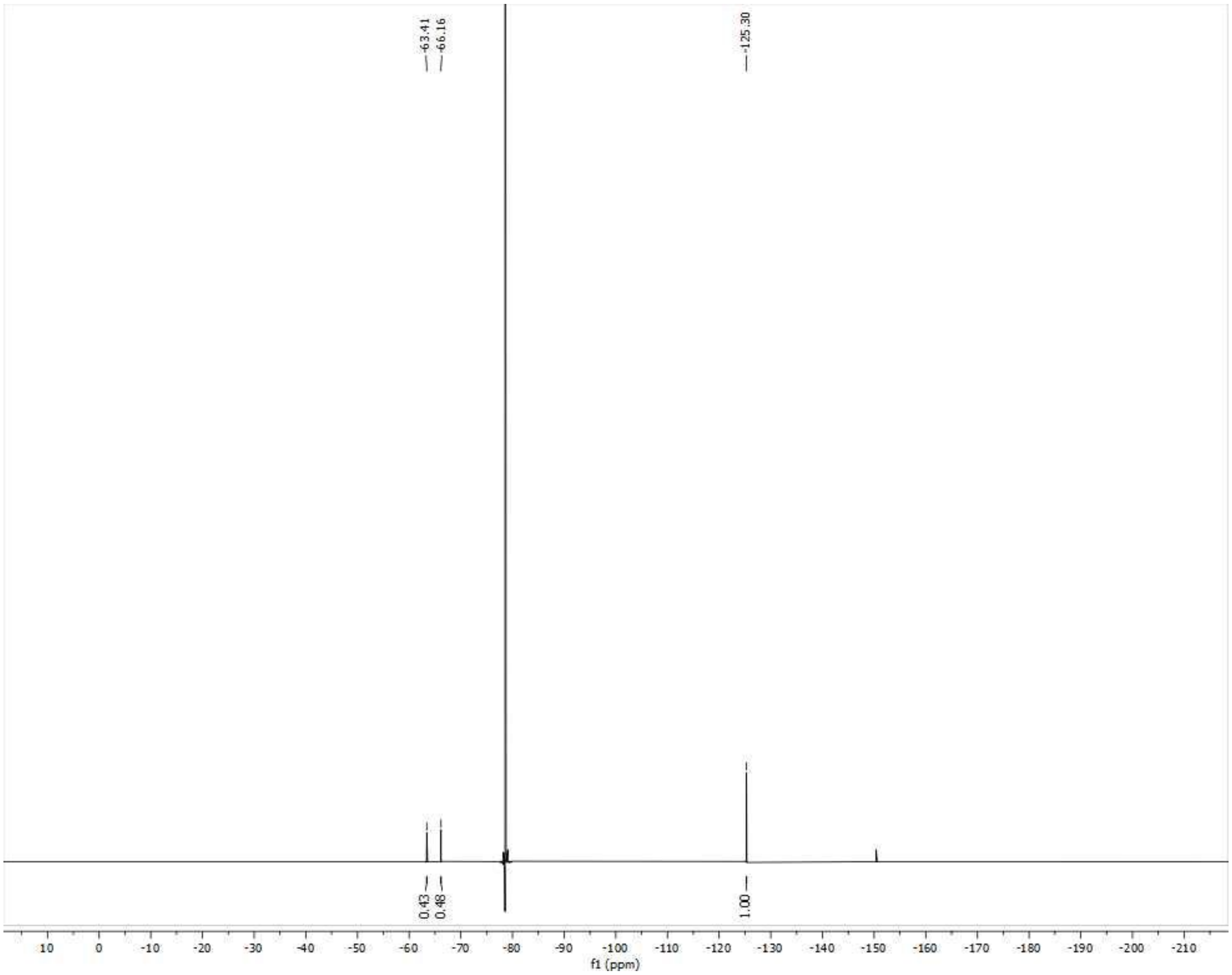

10. References

(1) Vogel, J. A.; Hammami, R.; Ko, A.; Datta, H.; Eiben, Y. N.; Labenne, K. J.; McCarver, E. C.; Yilmaz, E. Z.; Melvin, P. R. Synthesis of Highly Reactive Sulfone Iminium Fluorides and Their Use in Deoxyfluorination and Sulfur Fluoride Exchange Chemistry. *Organic Letters* **2022**, *24* (32), 5962-5966. DOI: 10.1021/acs.orglett.2c02232.

(2) Li, C.-K.; Tao, Z.-K.; Shoberu, A.; Zhang, W.; Zou, J.-P. Copper-Catalyzed Cross-Coupling of Alkyl and Phosphorus Radicals for C(sp3)–P Bond Formation. *Organic Letters* **2022**, *24* (32), 6083-6087. DOI: 10.1021/acs.orglett.2c02454.

(3) Trost, B. M.; Spohr, S. M.; Rolka, A. B.; Kalnmals, C. A. Desymmetrization of Phosphinic Acids via Pd-Catalyzed Asymmetric Allylic Alkylation: Rapid Access to P-Chiral Phosphinates. *Journal of the American Chemical Society* **2019**, *141* (36), 14098-14103. DOI: 10.1021/jacs.9b07340.

(4) Duan, H.; Xu, X.; Leng, K.; Zhang, S.; Han, Y.; Gao, J.; Yu, Q.; Wang, Z. A (4-fluorophenyl)(phenyl)phosphine oxide-modified epoxy resin with improved flame-retardancy, hydrophobicity, and dielectric properties. *Journal of Applied Polymer Science* **2021**, *138* (31), 50792. DOI: https://doi.org/10.1002/app.50792. Gafurov, Z. N.; Musin, L. I.; Sakhapov, I. y. F.; Babaev, V. M.; Musina, E. I.; Karasik, A. A.; Sinyashin, O. G.; Yakhvarov, D. G. The formation of secondary arylphosphines in the reaction of organonickel sigma-complex [NiBr(Mes)(bpy)], where Mes = 2,4,6-trimethylphenyl, bpy = 2,2′-bipyridine, with phenylphosphine. *Phosphorus, Sulfur, and Silicon and the Related Elements* **2016**, *191* (11-12), 1475-1477. DOI: 10.1080/10426507.2016.1212045.

(5) Xu, Q.; Zhao, C.-Q.; Han, L.-B. Stereospecific Nucleophilic Substitution of Optically Pure H-Phosphinates: A General Way for the Preparation of Chiral P-Stereogenic Phosphine Oxides. *Journal of the American Chemical Society* **2008**, *130* (38), 12648-12655. DOI: 10.1021/ja804412k.

(6) Dai, Q.; Liu, L.; Qian, Y.; Li, W.; Zhang, J. Construction of P-Chiral Alkenylphosphine Oxides through Highly Chemo-, Regio-, and Enantioselective Hydrophosphinylation of Alkynes. *Angewandte Chemie International Edition* **2020**, *59* (46), 20645-20650. DOI: https://doi.org/10.1002/anie.202009358.

(7) Chen, Q.; Zeng, J.; Yan, X.; Huang, Y.; Wen, C.; Liu, X.; Zhang, K. Electrophilic Fluorination of Secondary Phosphine Oxides and Its Application to P–O Bond Construction. *The Journal of Organic Chemistry* **2016**, *81* (20), 10043-10048. DOI: 10.1021/acs.joc.6b01932.

(8) Guo, J.; Kuang, C.; Rong, J.; Li, L.; Ni, C.; Hu, J. Rapid Deoxyfluorination of Alcohols with N-Tosyl-4-chlorobenzenesulfonimidoyl Fluoride (SulfoxFluor) at Room Temperature. *Chemistry – A European Journal* **2019**, *25* (30), 7259-7264. DOI: https://doi.org/10.1002/chem.201901176. Nielsen, M. K.; Ugaz, C. R.; Li, W.; Doyle, A. G. PyFluor: A Low-Cost, Stable, and Selective Deoxyfluorination Reagent. *Journal of the American Chemical Society* **2015**, *137* (30), 9571-9574. DOI: 10.1021/jacs.5b06307.

(9) Liu, N.; Mao, L.-L.; Yang, B.; Yang, S.-D. Copper-promoted oxidative-fluorination of arylphosphine under mild conditions. *Chemical Communications* **2014**, *50* (74), 10879-10882, 10.1039/C4CC04830J. DOI: 10.1039/C4CC04830J.

(10) Zhao, S.; Guo, Y.; Su, Z.; Wu, C.; Chen, W.; Chen, Q.-Y. Deoxyfluorination of Carboxylic, Sulfonic, Phosphinic Acids and Phosphine Oxides by Perfluoroalkyl Ether Carboxylic Acids Featuring CF2O Units. *Chinese Journal of Chemistry* **2021**, *39* (5), 1225-1232. DOI: https://doi.org/10.1002/cjoc.202000662.

(11) Li, Q.-W.; Zhang, X.-Y.; Lu, L.; Wu, Z.-Q.; Li, J.; Li, G.-Z.; Sun, K.; Yang, S.-D.; Yang, B. TFAA/DMSO-Promoted Fluorination of P(O)−H and P(O)−OH Compounds: Compatible Access to Fluorophosphonates and Phosphonofluoridates. *Advanced Synthesis & Catalysis* **2022**, *364* (5), 938-946. DOI: https://doi.org/10.1002/adsc.202101254.

(12) Brooks, R. J.; Bunton, C. A. Steric effects in the hydrolysis of methyl- and tert-butylphenylphosphinic chloride and fluoride. *The Journal of Organic Chemistry* **1975**, *40* (14), 2059-2064. DOI: 10.1021/jo00902a011. Hong, H.; Zhang, L.; Xie, F.; Zhuang, R.; Jiang, D.; Liu, H.; Li, J.; Yang, H.; Zhang, X.; Nie, L.; et al. Rapid one-step 18F-radiolabeling of biomolecules in aqueous media by organophosphine fluoride acceptors. *Nature Communications* **2019**, *10* (1), 989. DOI: 10.1038/s41467-019-08953-0.

(13) Wang, C.; Zhang, L.; Mou, Z.; Feng, W.; Li, Z.; Yang, H.; Chen, X.; Lv, S.; Li, Z. Direct 18F-Labeling of Biomolecules via Spontaneous Site-Specific Nucleophilic Substitution by F– on Phosphonate Prostheses. *Organic Letters* **2021**, *23* (11), 4261-4266. DOI: 10.1021/acs.orglett.1c01211.
